# Performance Prediction of Fundamental Transcriptional Programs

**DOI:** 10.1021/acssynbio.2c00593

**Published:** 2023-03-20

**Authors:** Prasaad T. Milner, Ziqiao Zhang, Zachary D. Herde, Namratha R. Vedire, Fumin Zhang, Matthew J. Realff, Corey J. Wilson

**Affiliations:** † School of Chemical & Biomolecular Engineering, Georgia Institute of Technology, Atlanta, Georgia 30332-2000, United States; ‡ School of Electrical and Computer Engineering, Georgia Institute of Technology, Atlanta, Georgia 30332-2000, United States; § School of Computer Science, Georgia Institute of Technology, Atlanta, Georgia 30332-2000, United States; ∥ School of Chemical & Biomolecular Engineering, Georgia Institute of Technology, Atlanta, Georgia 30332-2000, United States

**Keywords:** synthetic gene circuits, transcriptional programming, biological circuit prediction, synthetic transcription factors, antirepressors

## Abstract

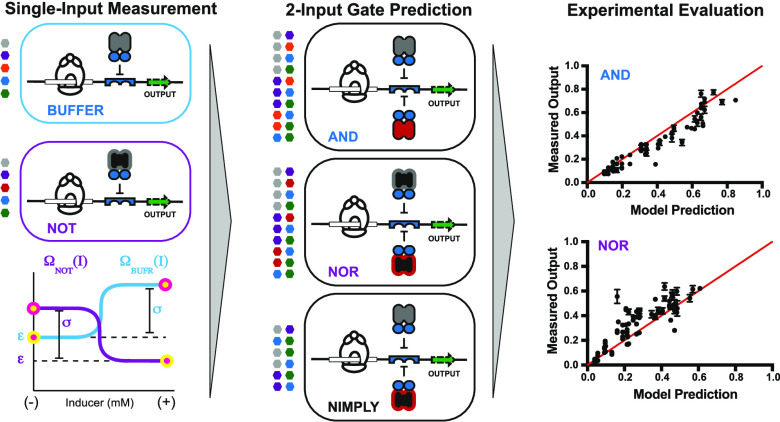

Transcriptional programming leverages systems of engineered transcription factors to impart decision-making (*e.g.*, Boolean logic) in chassis cells. The number of components used to construct said decision-making systems is rapidly increasing, making an exhaustive experimental evaluation of iterations of biological circuits impractical. Accordingly, we posited that a predictive tool is needed to guide and accelerate the design of transcriptional programs. The work described here involves the development and experimental characterization of a large collection of network-capable single-INPUT logical operations—*i.e.*, engineered BUFFER (repressor) and engineered NOT (antirepressor) logical operations. Using this single-INPUT data and developed metrology, we were able to model and predict the performances of all fundamental two-INPUT compressed logical operations (*i.e.*, compressed AND gates and compressed NOR gates). In addition, we were able to model and predict the performance of compressed mixed phenotype logical operations (A NIMPLY B gates and complementary B NIMPLY A gates). These results demonstrate that single-INPUT data is sufficient to accurately predict both the qualitative and quantitative performance of a complex circuit. Accordingly, this work has set the stage for the predictive design of transcriptional programs of greater complexity.

## Introduction

**Figure 1:**
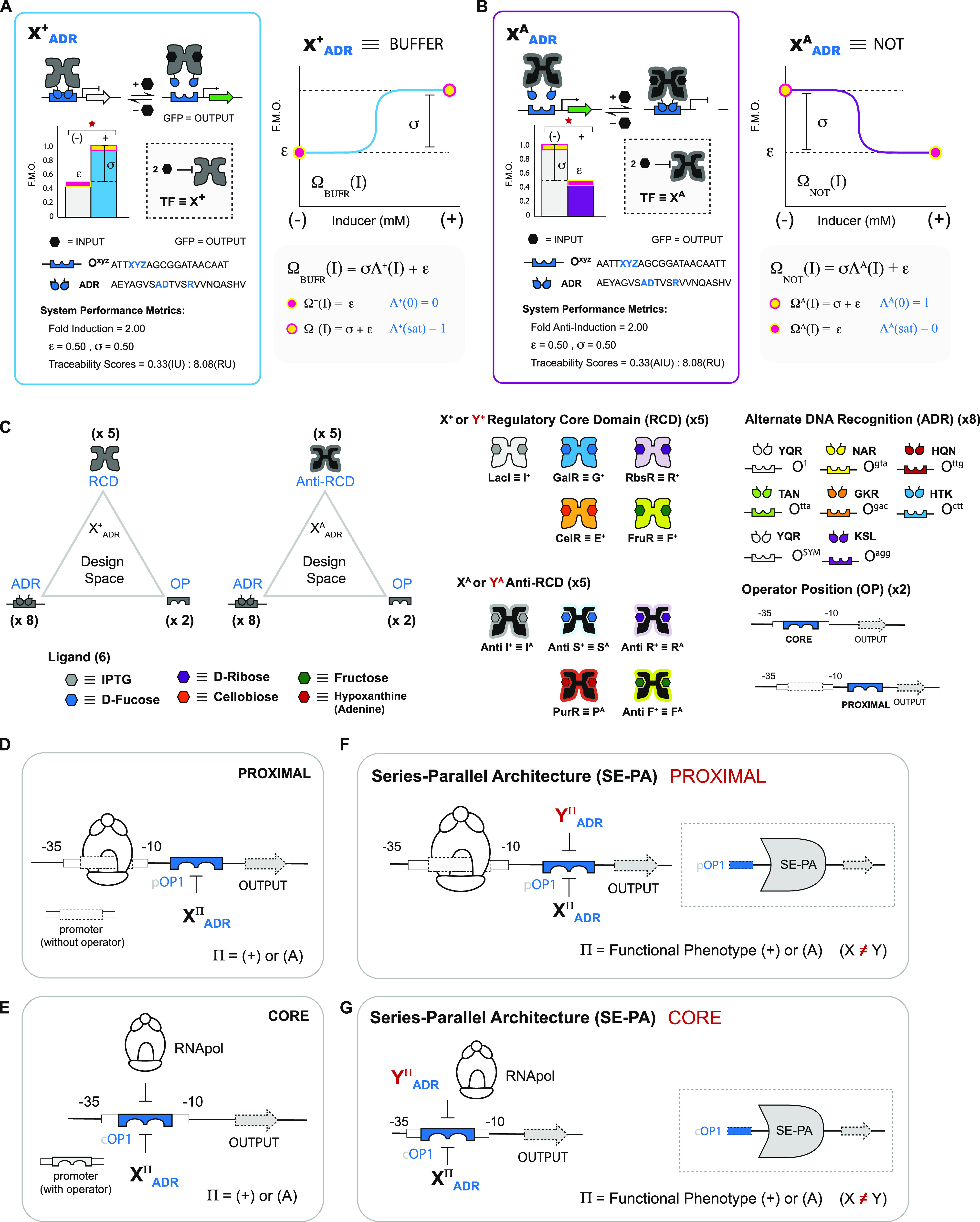
Modular components used in a design space. (A) Performance card of a repressor (X^+^) and abstraction of metrics to a logical BUFFER operation. (B) Performance card of an antirepressor (X^A^) and abstraction of metrics to a logical NOT operation. (C) Design space overview. Each of the 5 X^+^ or X^A^ RCDs can be paired with 1 of 8 ADRs and directed to 1 of 2 operator positions (OPs), resulting in a putative design space of 80 BUFFER and 80 NOT operations. Genetic architectures (D–G). (D) PROXIMAL architecture with an operator position downstream of the promoter. Transcription factor interferes with the RNA polymerase’s ability to transcribe DNA. (E) CORE architecture featuring an operator intercalated between the −35 and −10 hexamers of the synthetic *trc* promoter in *Escherichia coli*. The transcription factor competes with RNA polymerase binding to DNA. (F–G) Two-input architectures. (F) PROXIMAL SE-PA architecture, as shown in panel (D), with two transcription factors directed to the operator. (G) CORE SE-PA architecture, as shown in panel (E), with two transcription factors directed to the DNA operator.

Significant efforts have been devoted to engineering logical (decision-making) responses within a variety of chassis cells as a general proof-of-concept,^1−9^ and for a variety of logic-based applications—*e.g.*, biosensing,^7,10,11^ biological clocks,^12−15^ oscillators,^12,16−19^ controllers,^5,20−22^ and therapeutics.^23−28^ An emerging technology in biotic decision-making is transcriptional programming.^28−30^ Transcriptional programming makes use of fundamental logic principles by assigning an inducer molecule as the INPUT and by assigning a coupled regulated reading frame (coding or noncoding) as the OUTPUT. The operating constraints for said biotic programs are predicated on digitizing the INPUT to 0 or 1, where an INPUT 1 is achieved *via* the maintenance of saturating concentrations of the cognate inducer molecule—typically 10 mM. Digitizing the INPUT facilitates a constant level of OUTPUT—*e.g.*, the amount of green fluorescent protein (GFP) is present at a steady state. The fundamental 1-INPUT logical operations in transcriptional programming are (i) BUFFER gates regulated *via* engineered repressors (see Figure 1A) and (ii) NOT gates regulated *via* engineered antirepressors (see Figure 1B). Notably, antirepressors are an important and unique feature of transcriptional programming in that said transcription factors enable circuit compression—*i.e.*, the antirepressor eliminates the need for the inversion of a repressor function to achieve a NOT logical operation (see Supporting Note 1). Another important feature of transcriptional programing is the ability to direct two or more engineered transcription factors to a single DNA operator element—enabling the systematic construction of 2-INPUT logical operations, see Figure 1F,G and Supporting Note 2. The engineered transcription factors used in transcriptional programming were developed *via* modular design (see Figure 1C and Supporting Note 3). Briefly, the design template is based on the lactose repressor (LacI) topology, which can be decomposed into two functional regions: (i) a regulatory core domain (RCD) and (ii) a DNA binding domain (DBD). Given that LacI belongs to a large family of homologous transcription factors with a similar topology that can process different INPUT ligands and bind to different DNA operators,^31,32^ a putative design space can be gleaned. Accordingly, several groups have demonstrated that functional chimeras can be constructed based on the given engineering principles.^29,33−37^

Wilson et al., in a collection of studies, posited and demonstrated that modular design could be applied to engineered domains—*i.e.*, alternate (engineered) DNA binding functions,^29,35^ alternate (engineered) allosteric communication,^30,35,37,38^ and alternate (engineered) ligand binding functions^36,37^—to create a system of transcription factors (repressors and antirepressors) that are network capable. To date, using this collection of engineered repressors and antirepressors, transcriptional programs are designed and constructed intuitively—though with an apparent rule set (see Supporting Note 4). As a result, transcriptional programs often require iterative tuning and redesign. While intuitive program design and construction have proven to be effective, the said approach is time-consuming and expensive. What is needed now is a means to predictively design transcriptional programs—*i.e.*, in terms of qualitative outcomes and quantitative performances. To accomplish the aforesaid, in this study, we leverage and build upon the model introduced by Zong et al.^39^ to predict multiple-INPUT single-OUTPUT (MISO) logical operations from single-INPUT single-OUTPUT (SISO) data—without requiring parameter fitting of the model from MISO experiments. Namely, we have systematically designed, built, and tested a large collection of BUFFER SISO and NOT SISO with corresponding metrology for the given fundamental logical operations. In turn, we have leveraged our standardized SISO data to design, build, and test the corresponding set of MISO logical operations (*via* transcriptional programming) allowed at a single operator–promoter position—*i.e.*, forming AND, NOR, A NIMPLY B, and B NIMPLY A operations. Finally, we show that using simple (coarse-grained) models, we can qualitatively design and quantitatively predict the fundamental performances of MISO logical operations from SISO data only—establishing the foundation for the predictive design of transcriptional programs.

## Results

### Design, Metrology, and Modeling for Single-INPUT Single-OUTPUT (SISO) Logical Operations

In previous studies, we established metrology for SISO X_ADR_^+^ BUFFER^29^ and SISO X_ADR_^A^ NOT^30^ gate performance—which we extend and further develop in this study. For a given engineered transcription, factor X (or Y) defines the regulatory core domain (RCD), the superscript “+” defines the repressor phenotype (Figure 1A), the superscript “A” defines the antirepressor phenotype (Figure 1B), and the subscript defines the alternate (engineered) DNA recognition (ADR) function. The putative design space for said engineered transcription factors is given in Figure 1C.

Briefly, given a transcription factor and cognate operator DNA element regulating a green fluorescent protein (GFP) OUTPUT, the performance metrics of a BUFFER gate can be given by the (i) fold induction, (ii) repression strength, and (iii) two-part traceability score—*i.e.*, induction units (IU) and repression units (RU)—relative to a reference system (see Figure 1A, and Supporting Note 5). Likewise, for a NOT gate, the performance can be reported by similar metrics; however, (i) fold anti-induction replaces fold induction to reflect the change in the phenotype, and the two-part traceability score is modified accordingly—*i.e.*, reporting anti-induction units (AIU)—relative to the same reference system (see Figure 1B, and Supporting Note 5).

In addition to reporting the metrology for a given SISO operation, we can model the induction profile for an experimentally verified SISO BUFFER operation *via* a coarse-grained binding function defined as
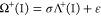
1where σ is a constant representing the maximum fluorescence—relative to the basal expression of the OFF state, Λ^+^(I) is the coarse-grained Hill function that can assume a value of 0 or 1, and ε represents fluorescence in the absence of an inducer—*i.e.*, the OFF state (see Figure 1A). Given that the transition region cannot maintain a setpoint, we excluded intermediate INPUT concentrations, analogous to the naïve Hill model reported by Zong et al.^39^—as we are only interested in the steady-state (binary) performance of a given open-loop operation.

Likewise, to model the performance of a given SISO NOT gate, we used an analogous coarse-grained binding function— though for antirepression—defined as
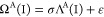
2where σ is a constant representing the maximum fluorescence, minus the ligand—relative to the basal expression of the OFF state, Λ^A^(I) is the coarse-grained antithetical Hill function for antirepression where 0-INPUT corresponds to the ON state and 1-INPUT corresponds to the OFF state, and ε represents fluorescence in the presence of an inducer—*i.e.*, the OFF state (see Figure 1B).

In this study, we designed, built, and tested 80 BUFFER operations and 80 NOT operations congruent with the design space given in Figure 1C—*i.e.*, 40 systems at the PROXIMAL position (see Figure 1D and Supporting Figure S1) and 40 systems at the CORE position (see Figure 1E and Supporting Figure S2) for each putative logical operation. In addition, we performed metrological analysis on and modeling of said transcription factors (see Supporting Note 5 and Supporting Data Set 1). For the BUFFER operations, the design space consisted of 5 nonsynonymous regulatory core domains, 8 alternate DNA binding operations, and 2 operator positions (see Figure 1C). Similarly, the design space for the purported NOT operations was composed of 5 anti-RCDs (4 of which were antithetical to a given X^+^), with complete overlap with respect to the given alternate DNA binding functions and cognate DNA operators. Out of the 40 transcription factors tested at the PROXIMAL position, 35 (∼87%) resulted in objective (qualitative) BUFFER logic gating—*i.e.*, having statistically significant differences between the ON state (with ligand) and OFF state (without ligand) based on a student *t*-test. Whereas 38 (∼95%) out of the 40 transcription factors tested at the CORE position resulted in BUFFER logic (see Supporting Data Set 1). Together, this resulted in 73 (out of 80 – or ∼91%) functional BUFFER SISO control systems. In contrast, 36 (out of 40) PROXIMAL and 40 (out of 40) CORE antirepressor transcription factors resulted in objective NOT logic gate performance—for a total of 76 (∼95%) operational NOT SISO (see Supporting Data Set 1). We posited that any differences observed in performance between the PROXIMAL and CORE operator positions for a given (equivalent) logical operation can be attributed to the variation in binding competition between the two sites (see Figure 1D,E). In general, for a given logical operation, the CORE position had fewer nonoperational gates relative to the PROXIMAL position. Nonoperational gates can be classified by two additional phenotypes: (i) super-repressor (X^S^) or (ii) nonfunctional (X^–^); see Supporting Figure S3. Notably, the majority of the nonoperational SISO gates were classified as nonfunctional (X^–^). Finally, the overlap in DNA binding functions for said BUFFER and NOT operations can facilitate networked cooperation between SISO—*i.e.*, when sets of transcription factors are directed to a single DNA operator element. The aforesaid networking capability can enable the bottom-up construction of multiple-INPUT single-OUTPUT (MISO) logical operations—illustrated in the following sections.

### Developing Design Rules for MISO AND Logical Gate Construction from BUFFER SISO Data

The construction of an AND (MISO) logical gate *via* transcriptional programming can be achieved using either a (i) series (SERI) (Supporting Figure S4) or (ii) series-parallel (SE-PA) (Figure 1F,G) genetic architecture. Here, we focused on the construction of 2-INPUT AND gates using the SE-PA iteration, as this particular design simplifies the accounting of independent transcription factor operator interactions—as both transcription factors are directed to the same DNA element. To identify putative sets of BUFFER logical operations that can be paired (*via* SE-PA DNA operators) to form objective 2-INPUT AND logical gates, we initially used a two-step decision process informed by the SISO data alone. Namely, first, we identified all BUFFER SISO logical operations with measurable dynamic ranges (*i.e.*, statistical differences between the ON and OFF states), and in the second tier of the decision process, we evaluated compatibility between two networked transcription factors. When sufficient inequality (*i.e.*, MISO compatibility) exists between the ON state and OFF state of SE-PA networked BUFFER gates, we posited that an objective 2-INPUT AND logic gate can be constructed—see example in Supporting Figure S5A. In this illustration, SISO data for I_YQR_^+^ was compared to SISO data from R_YQR_^+^—where X ≡ I or R, ADR ≡ YQR, and + ≡ repressor phenotype. Here, the OFF state of I_YQR_^+^ has a lower threshold relative to the ON state of R_YQR_^+^—likewise for the complementary ON and OFF states. Accordingly, we can regard the two BUFFER operations as compatible with respect to forming a 2-INPUT, SE-PA directed AND logic gate—*i.e.*, when directed to a cognate operator at a fixed position. In contrast, given two functional BUFFER gates, a potential incompatibility can arise when a pair of logical operations do not have sufficient inequality (*i.e.*, MISO incompatibility) between the ON state of one transcription factor (*i.e.*, F_YQR_^+^), relative to the OFF state of the complementary (networked) transcription factor (*i.e.*, E_YQR_^+^)—see Supporting Figure S5B.

**Figure 2:**
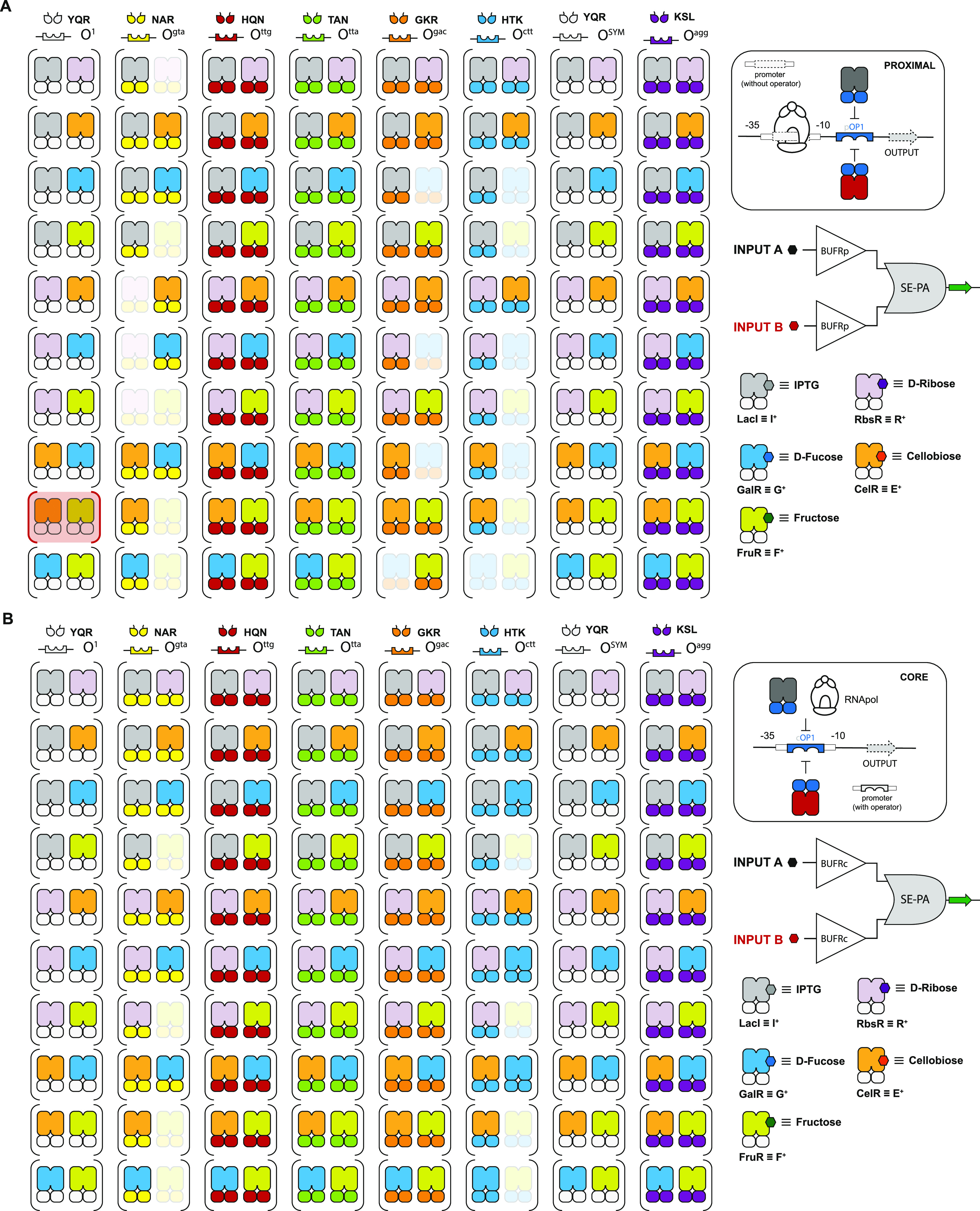
Combinatorial set of SE-PA AND gates. (A) Illustration of nonsynonymous repressor pairs combined with 8 ADRs yields 80 putative PROXIMAL SE-PA AND gates. Repressors classified as nonoperational (see Figure S3) are shown faded and incompatible repressor pairs (see Figure S5) are highlighted in red. Consideration of nonoperational pairs results in a reduced space composed of 61 PROXIMAL SE-PA AND gates. (B) CORE SE-PA architecture AND gates. Elimination of nonoperational and incompatible repressors results in 72 CORE SE-PA AND gates.

Unmitigated, the pairwise (2-INPUT) network space for AND gate construction is represented by 80 operations at the PROXIMAL position and 80 operations at the CORE position. However, with the initial constraints imposed by the number of functional BUFFER SISO (*i.e.*, 35 PROXIMAL, 38 CORE), the putative network space is reduced to 62 PROXIMAL AND gates (see Figure 2A) and 72 CORE AND gates (see Figure 2B)—without factoring in putative incompatibilities. Including said incompatibilities, the putative networked space is further reduced by one—*i.e.*, to 61 PROXIMAL AND gates and 72 CORE AND gates—resulting in a total of 133 2-INPUT logical operations that are purportedly functional (see Figure 2).

### Building, Testing, and Modeling AND Logic Gates

After designing said AND logic gates, we built and tested the complete set of gates with the predicted function—*i.e.*, 61 PROXIMAL AND gates (see Figure 2A) and 72 CORE AND gates (see Figure 2B). Briefly, each AND gate was experimentally tested in the same *E. coli* chassis cell as the SISO systems and regulated the same GFP OUTPUT. All of the AND gates were functional with qualitative (objective) performances congruent with the intended logical operation (see Supporting Data Set 2). To further validate our qualitative predictions, we constructed several systems that were predicted to be nonoperational (see Supporting Data Set 3). In general, both sets of data affirmed our qualitative prediction. Namely, on average, the nonoperational gates did not result in objective logic gating (or had poor quantitative performance—*i.e.*, had dynamic ranges <2) when experimentally tested.

**Figure 3:**
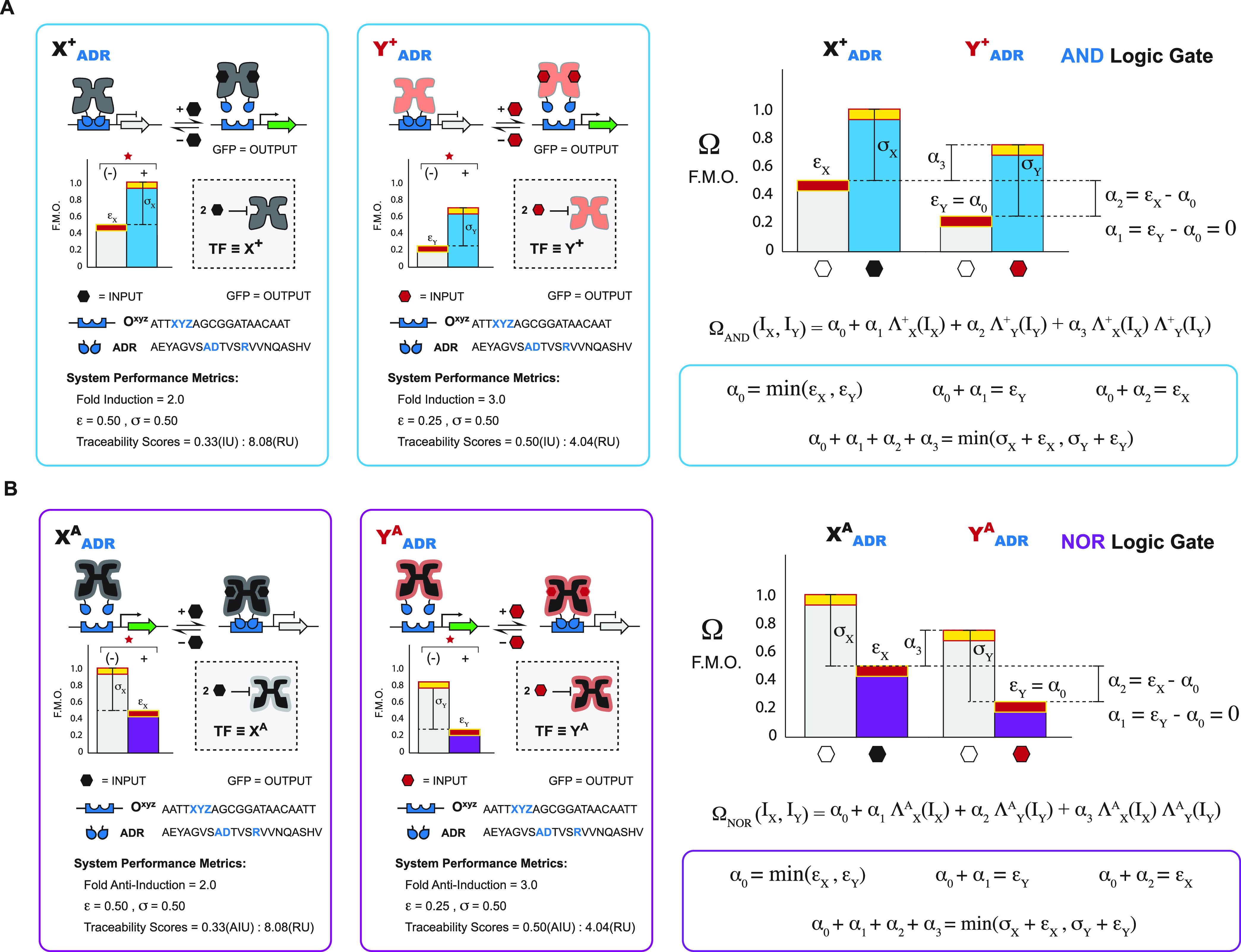
SE-PA AND operation and NOR operation predictive models using BUFFER SISO and NOT SISO parameters. (A) AND gate logic is modeled using a quadratic function of I_X_ and I_Y_, which control the repressor state functions Λ_X_^+^ and Λ_Y_^+^, respectively. Each term has a coefficient α_0_, α_1_, α_2_, or α_3_, which are estimated as functions of BUFFER gate parameters ε_X_, ε_Y_, σ_X_, and σ_Y_ (also see Figure 1A). Functions for parameters α_0_, α_1_, α_2_, and α_3_ are derived using four assumptions corresponding to each INPUT condition. (B) NOR gate logic is modeled analogous to the AND logic, however, with a pair of NOT gates parameterized with antirepressor state functions Λ_X_^A^ and Λ_Y_^A^ (also see Figure 1B). Given that Λ_X_^A^ and Λ_Y_^A^ functions capture the ON–OFF state inversion from the repressor to the antirepressor phenotype, α_0_, α_1_, α_2_, and α_3_ parameters are estimated with the same functions for both AND and NOR models.

To better interpret and predict the quantitative performance of our 2-INPUT AND logic gates, we constructed a coarse-grained model—defined as follows:
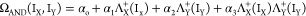
3where Ω_AND_ is the OUTPUT expression, Λ_X_^+^ is the Hill state function of repressor X^+^, Λ_Y_^+^ is the Hill state function of repressor Y^+^, I_X_ is the inducer state of X^+^ (either 0 or 1), I_Y_ is the inducer state of Y^+^ (either 0 or 1), and α_o_, α_1_, α_2_, and α_3_ are parameters determined from the SISO gates by a set of four equations; see Figure 3A. Qualitatively, α_o_ is the minimum OUTPUT of the gate (or overall leakiness, *i.e.*, ε_X_ or ε_Y_), α_1_ is the OUTPUT increase (from the baseline α_o_) in response to I_X_, α_2_ is the OUTPUT increase (from the baseline α_o_) in response to I_Y_, and α_3_ is the OUTPUT increase from the maximum OFF state to the ON state (also see Supporting Note 6).

**Figure 4:**
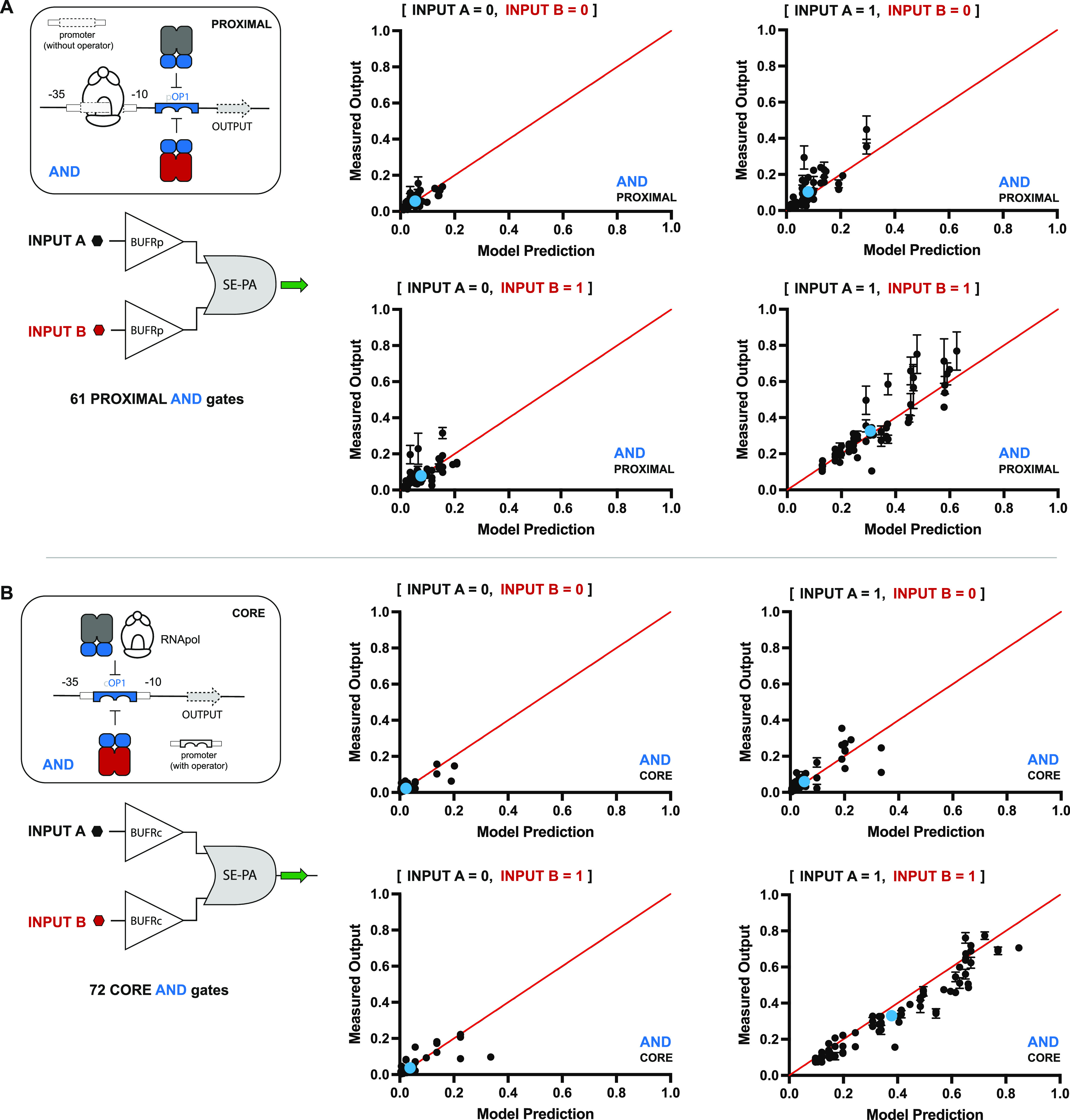
Results showing the correlation between the predicted and measured OUTPUT of 133 SE-PA AND gates. Under-predictions and over-predictions fall above and below the theoretical value of 1 (red line), respectively. (A) Correlation results for 61 PROXIMAL SE-PA AND gates across the 4 INPUT conditions. INPUTs A and B correspond to repressors X^+^ and Y^+^, respectively, and can be inferred from each BUFFER pair depicted in Figure 2A. (B) Correlation between the predicted and measured OUTPUT of 72 CORE SE-PA AND gates. INPUTs A and B correspond to repressors X^+^ and Y^+^ and can be inferred from each BUFFER pair depicted in Figure 2B. Tracer data is given as sets of blue dots, illustrating the mean performance prediction across all systems.

In general, the AND gate model predicted the quantitative performances of experimental outcomes with a high degree of accuracy—with a mean error (measured output/predicted output) of 1.256; see Figure 4 and Supporting Figure S6A. Only ∼12% of the values had a 2-fold or greater difference relative to the predicted value—*i.e.*, the model could accurately predict the qualitative and quantitative performance of measured values in the context of the AND logic in ∼88% of cases. Interestingly, PROXIMAL AND logic gates (Figure 4A) had a greater degree of spread—*i.e.*, for a given data set per individual operation—relative to the CORE AND logic gates (Figure 4B). We attributed this difference to the presence of a variable ‘5-UTR (untranslated region) in the PROXIMAL systems, which can variably affect ribosome binding and thus the apparent level of translation.

### Developing Design Rules for MISO NOR Logical Gate Construction from NOT SISO Data

**Figure 5:**
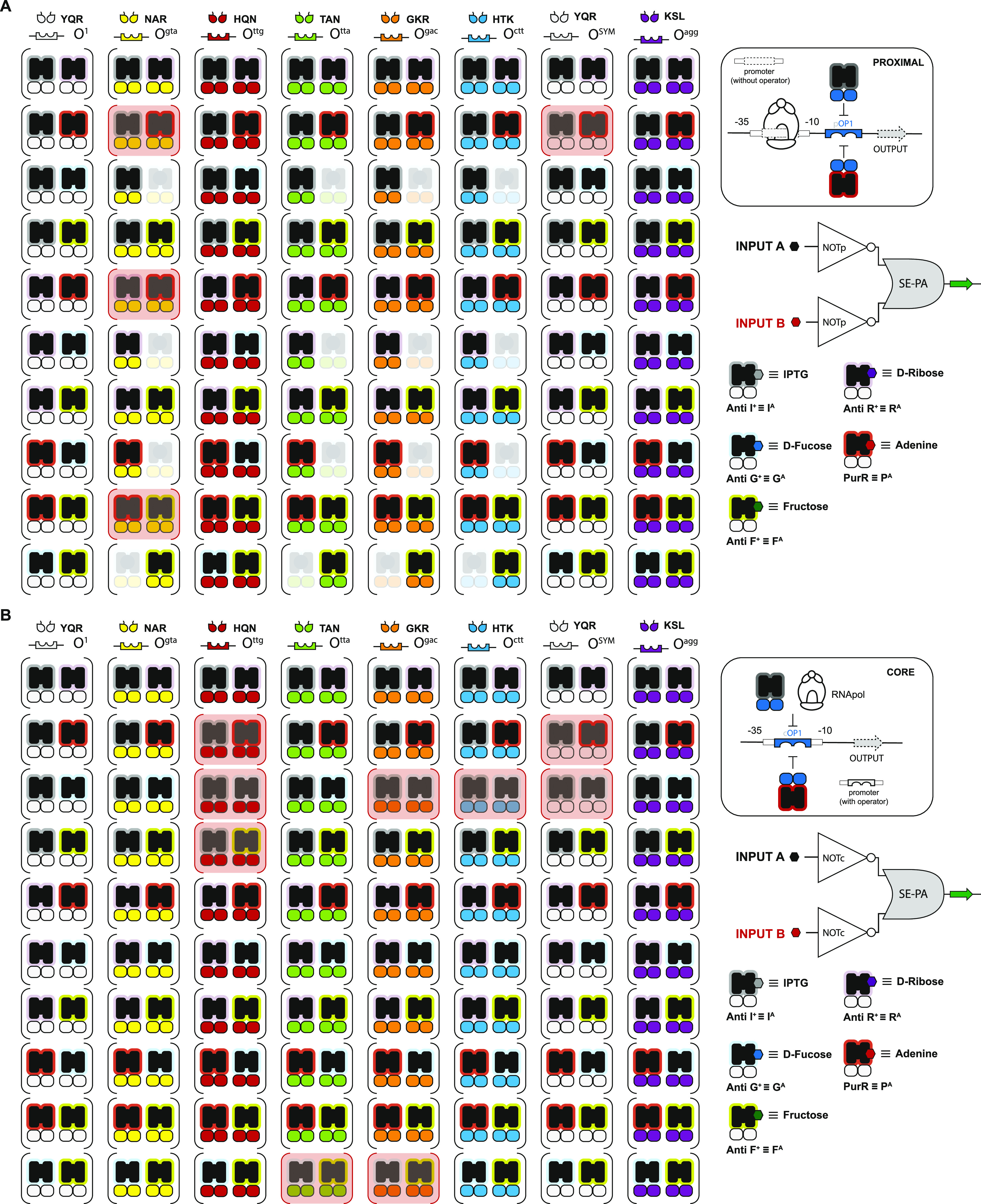
Combinatorial set of 131 SE-PA NOR gates. (A) Illustration of nonsynonymous antirepressor pairs combined with 8 ADRs yields 80 putative PROXIMAL SE-PA NOR gates. Antirepressors classified as nonoperational (see Figure S3) are shown faded and incompatible antirepressor pairs (see Figure 7B) are highlighted in red. These nonoperational pairs result in a reduced space of 60 proximal SE-PA NOR gates. (B) CORE SE-PA architecture NOR gates. Elimination of nonoperational and incompatible antirepressors results in 71 CORE SE-PA NOR gates.

Akin to our workflow for identifying functional AND gates, a similar process can be used to identify putative 2-INPUT NOR logical gates—paired *via* SE-PA operators. Namely, the initial selection (design) criteria required the identification of said NOT SISO operations with statistically significant differences between the ON state (without a ligand) and the OFF state (with a ligand)—*i.e.*, adequate dynamic ranges for a set of network-capable antirepressors. The second hierarchical design criteria required sufficient inequality between complementary ON and OFF states (see Supporting Figure S7A). In other words—with the design goal of forming a 2-INPUT NOR logical operation—incompatibility between two NOT gates occurs when said SISO operations do not have a sufficient distinction between the ON state of one operation (P_YQR_^A^) relative to the OFF state of the complementary operation (I_YQR_^A(9)^); see Supporting Figure S7B. The unrestricted, 2-INPUT network space for NOR gate construction is represented by 80 operations at the PROXIMAL position and 80 operations at the CORE position. However, when accounting for the nonfunctional SISO logic gates at the PROXIMAL position, the network space is reduced to 64 putative NOR gates (Figure 5A). Moreover, including the four incompatible sets, the PROXIMAL network space is reduced to 60 putative NOR gates. In contrast, the CORE position did not contain any nonfunctional NOT gates. However, nine putative incompatible NOR sets were predicted at the CORE position—resulting in 71 putative NOR gates (Figure 5B).

### Building, Testing, and Modeling NOR Logic Gates

Inspired by the workflow developed for the AND logic gates, we built and experimentally tested all putative NOR gates—60 putative PROXIMAL NOR gates and 71 putative CORE NOR gates. In addition, we constructed a model to better interpret and predict the quantitative performance of our 2-INPUT NOR logic gates given the corresponding SISO data as follows:
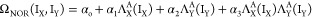
4where Ω_NOR_ is the OUTPUT expression, Λ_X_^A^ is the Hill state function of antirepressor X^A^, Λ_Y_^A^ is the Hill state function of antirepressor Y^A^, I_X_ is the inducer state of X^A^ (either 0 or 1), I_Y_ is the inducer state of Y^A^ (either 0 or 1), and α_o_, α_1_, α_2_, and α_3_ are parameters determined by the set of four equations described previously; also see Figure 3B and Supporting Note 7.

**Figure 6:**
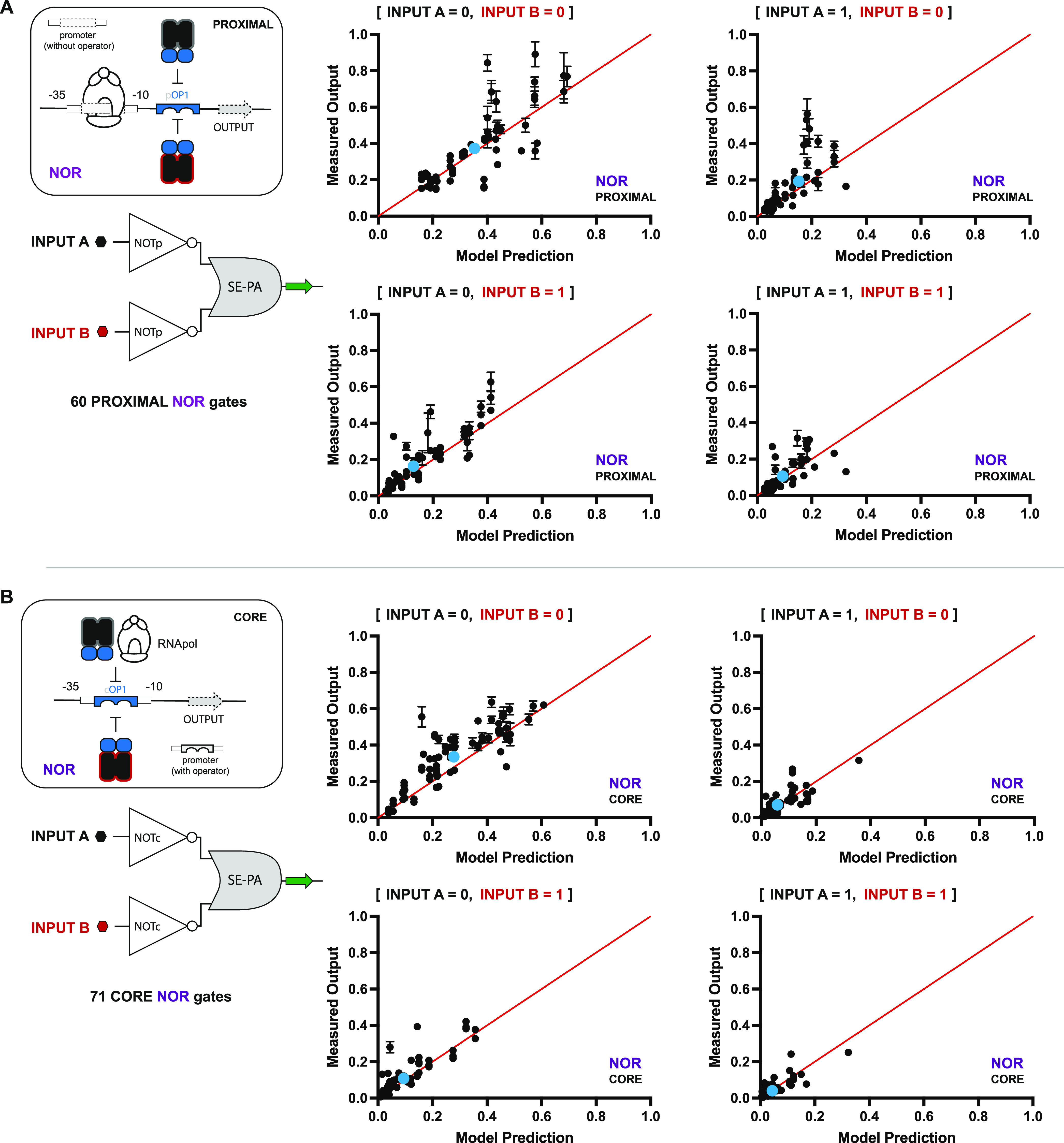
Results showing the correlation between the predicted and measured OUTPUT of 131 SE-PA NOR gates. Under-predictions and over-predictions fall above and below the theoretical value of 1 (red line), respectively. (A) Correlation results for 60 PROXIMAL SE-PA NOR gates across the 4 INPUT conditions. INPUTs A and B correspond to antirepressors X^A^ and Y^A^, respectively, and can be inferred from each NOT pair depicted in Figure 5A. (B) Correlation between the predicted and measured OUTPUT of 71 CORE SE-PA NOR gates. INPUTs A and B correspond to antirepressors X^A^ and Y^A^ and can be inferred from each NOT pair depicted in Figure 5B. Tracer data is given as sets of blue dots, illustrating the mean performance prediction across all systems.

Qualitatively, all of the predicted NOR gates were functional—*i.e.*, validated by the experiment (see Figure 6). Quantitatively, the model accurately predicted the experimental values in ∼89% of cases, with a mean error of 1.28 (see Supporting Figure S6B). Moreover, select nonoperational data affirmed our expectations—*i.e.*, on average, objective (qualitative) NOR gating was not observed or had poor performance (see Supporting Data Set 3). Congruent with the observation made for differences in performance at the PROXIMAL versus CORE positions for the AND gates, the tested NOR gates had similar differences in data spread between the two operator positions. Namely, PROXIMAL NOR gates (Figure 6A) had a greater degree of spread in the standard deviation per data point relative to CORE NOR gates (Figure 6B)—which we again attributed to a variable ‘5-UTR in the PROXIMAL operations.

### Building, Testing, and Modeling Nonimplication Logic Gates

**Figure 7:**
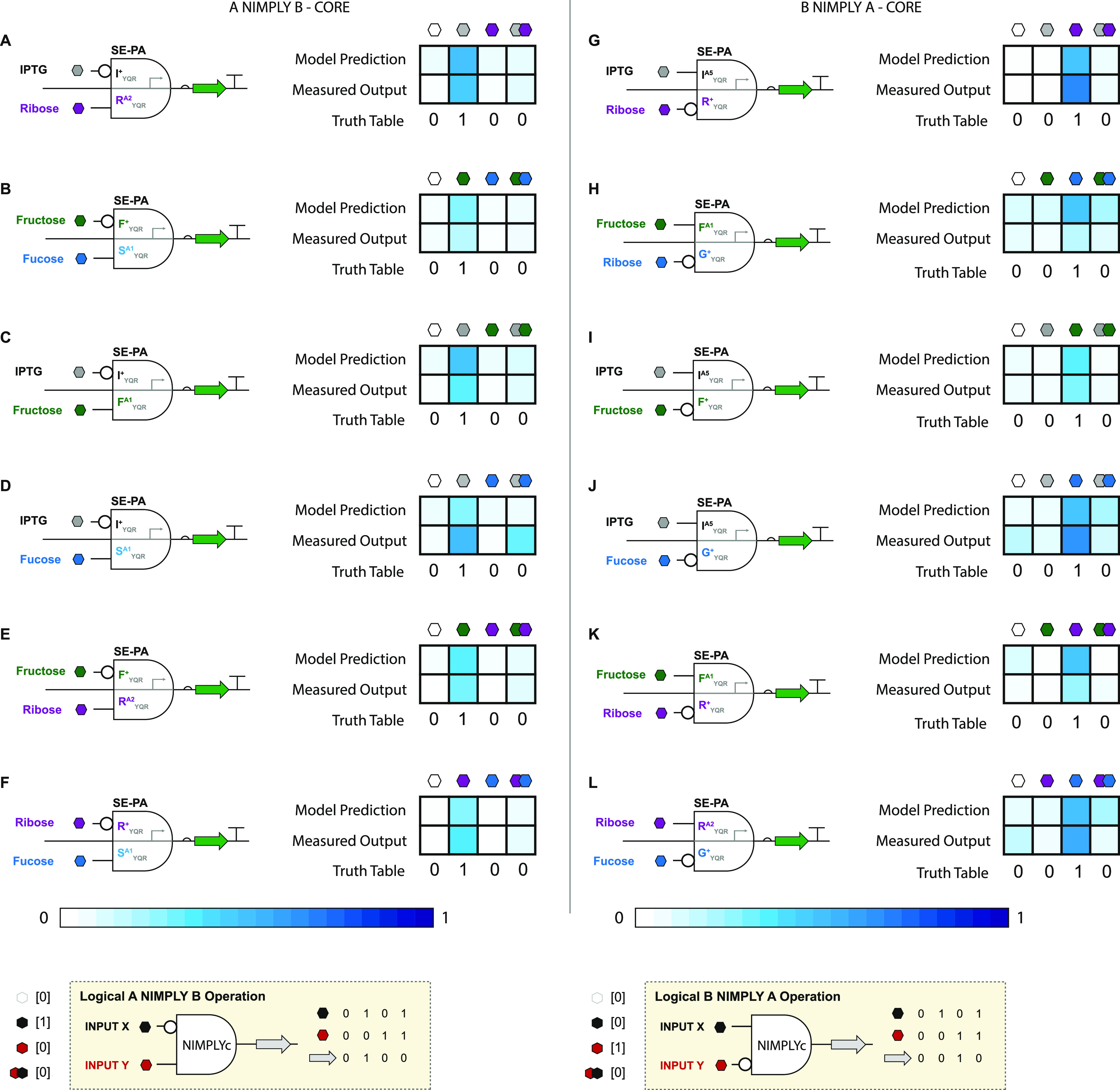
Results for 12 SE-PA NIMPLY logic gates at the CORE operator position. Signal INPUTs (IPTG, ribose, fucose, and fructose) were selected based on the ability to perform both the BUFFER and NOT logic (*i.e.*, induce repressors and antirepressors). This corresponds to 6 A and B INPUT pairs which cover the full combinatorial space for the NIMPLY logic. (A–F) A NIMPLY B logic employing a repressor (X_ADR_^+^), which responds to INPUT A, and an antirepressor (Y_ADR_^A^), which responds to INPUT B. (G–L) Complimentary A NIMPLY B logic utilizing an antirepressor (X_ADR_^A^) and repressor (Y_ADR_^+^).

In principle, we can direct (network) two transcription factors with divergent phenotypes (*i.e.*, repressor and antirepressor) to a shared (SE-PA) DNA operator. This class of simply mixed networks objectively results in an A NIMPLY B logical operation; see Supporting Figure S8. Likewise, using the complementary set of transcription factors (*i.e.*, R_YQR_^+^ and I_YQR_^A^), we can generate the complementary logical operation B NIMPLY A; see Supporting Figure S8. In the given illustrations, we qualitatively predicted that repressor I_YQR_^+^ can be paired with antirepressor R_YQR_^A^, and this operation will only produce an OUTPUT when the INPUT signal that corresponds to the repressor is present; see Figure 7 and Supporting Figure S9.

**Figure 8:**
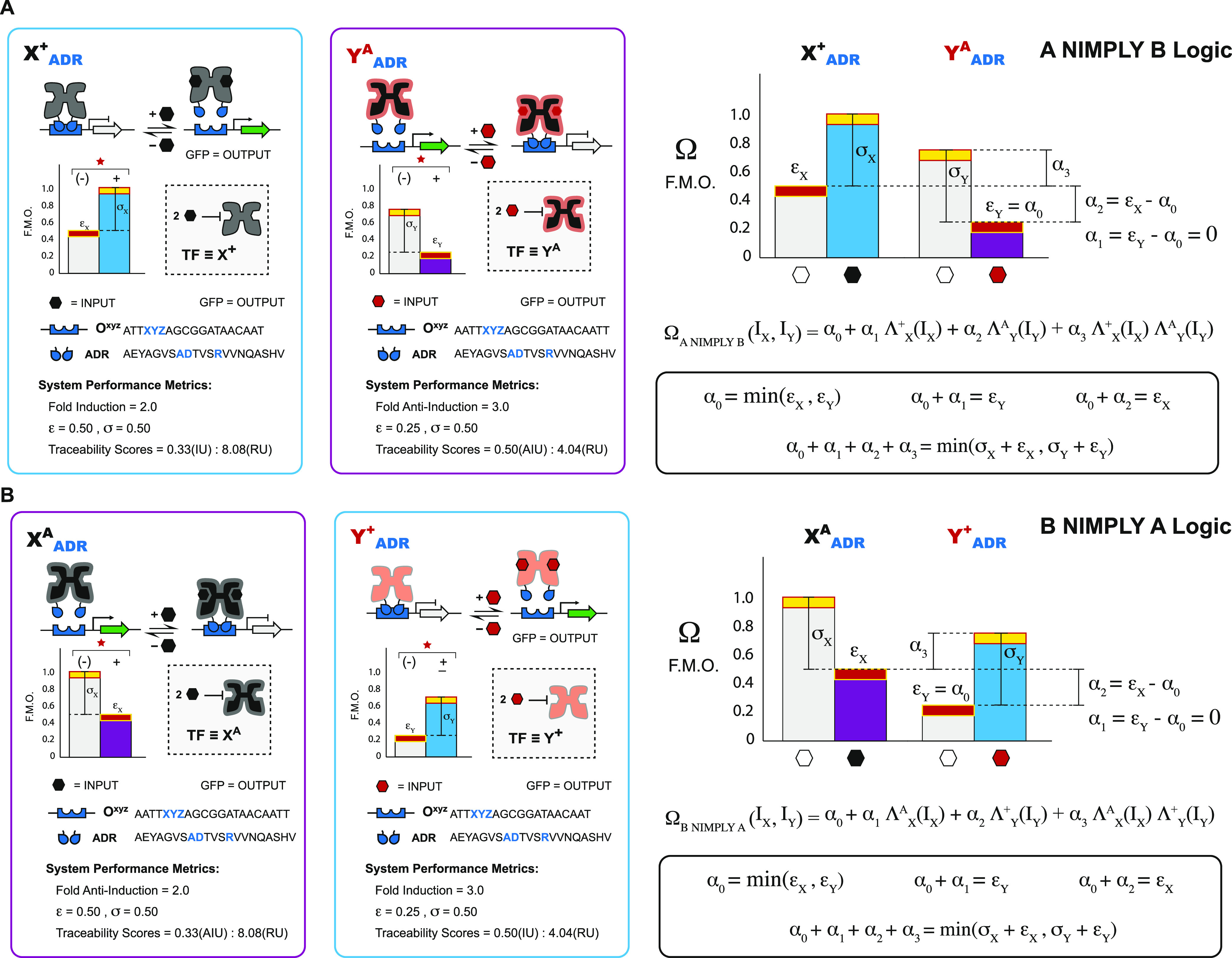
NIMPLY predictive models using BUFFER and NOT gate parameters. (A) A NIMPLY B gate logic is modeled using a quadratic function of I_X_ and I_Y_, which controls the repressor state function Λ_X_^+^ and antirepressor state function Λ_Y_^A^. Each term has a coefficient α_0_, α_1_, α_2_, or α_3_, which are estimated as functions of BUFFER and NOT gate parameters ε_X_, ε_Y_, σ_X_, and σ_Y_. Functions for parameters α_0_, α_1_, α_2_, and α_3_ are derived using a set of four assumptions corresponding to each INPUT condition. (B) B NIMPLY A gate logic is modeled analogous to the A NIMPLY B logic but with an antirepressor state function Λ_X_^A^ and repressor state function Λ_Y_^+^.

As with previous 2-INPUT operations, we can model nonimplication logic gates to better interpret quantitative performances. Namely, for 2-INPUT A NIMPLY B logic gates, we modified the model shown in eq 3 to include one repressor and one antirepressor state function pertaining to both BUFFER and NOT SISO logic. The model for A NIMPLY B is shown below:
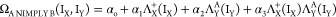
5where Ω_A NIMPLY B_ is the OUTPUT expression, Λ_X_^+^ is the Hill state function of repressor X^+^, Λ_Y_^A^ is the Hill state function of antirepressor Y^A^, I_X_ is the inducer state of X^+^ (either 0 or 1), I_Y_ is the inducer state of Y^A^ (either 0 or 1), and α_o_, α_1_, α_2_, and α_3_ are parameters determined by the set of four equations described previously; also see Figure 8A and Supporting Note 8.

The model for 2-INPUT B NIMPLY A logic gates follows that described above for A NIMPLY B gates, with the modification that TFs X and Y phenotypes are switched so that this system contains antirepressor X^A^ and repressor Y^+^. The model is, therefore
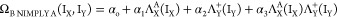
6where Ω_B NIMPLY A_ is the OUTPUT expression, Λ_X_^A^ is the Hill state function of antirepressor X^A^, Λ_Y_^+^ is the Hill state function of repressor Y^+^, I_X_ is the inducer state of X^A^ (either 0 or 1), I_Y_ is the inducer state of Y^+^ (either 0 or 1), and α_o_, α_1_, α_2_, and α_3_ are parameters determined by the set of four equations described; also see Figure 8B and Supporting Note 9.

Given that the total combinatorial space for said nonimplication logic gates is represented by 160 operations per operator position (*i.e.*, a total of 320 logical operations), we selected 12 exemplars (*i.e.*, 24 when considering CORE and PROXIMAL operator positions) to illustrate gate construction and to test our model’s accuracy given the corresponding SISO data (see Figure 7, Supporting Figure S9, and Supporting Data Set 2). Qualitatively, all of the tested nonimplication logic gates performed as expected. Moreover, the model accurately predicted the qualitative performance in all cases and quantitatively predicted the performance of said nonimplication gates in ∼86% of the tested cases.

## Discussion

As circuit complexity increases in synthetic biology, there is a growing need to predict the performance of a desired complex system (*i.e.*, qualitatively and quantitatively) prior to construction. Ideally, this would begin with modeled interaction data—which would involve comprehensive predictions of protein–ligand interactions, protein–DNA interactions, and allosteric communication in terms of binding energetics. While such predictive capabilities are not practical, the extrapolation of simple SISO data to predict the performance of MISO logical operations has shown great promise.^39^ Accordingly, in this work, we have taken the first steps toward predicting circuit performance for simple (single promoter) transcriptional programs that can potentially scale to more complex operations that involve feeding forward information. Moreover, we achieved the aforesaid using coarse-grained models. In principle, we could increase the granularity of the models *via* the inclusion of transition state data similar to Zong et al.^39^ The value added using a finer-grained approach could potentially allow for the inclusion of information regarding the sensitivity of a transcription factor to a cognate ligand—which could be important if knowing the minimal amount of ligand to achieve a setpoint is important. However, as demonstrated by Zong et al.,^39^ the use of a fine-grained approach did not result in predictive capability within the transition region, which can be attributed to the high degree of fluctuation in OUTPUT dictated by small changes in INPUT concentrations. In other words, the maintenance of a setpoint in the transition region for a given pair of SISO operations that form a MISO operation cannot be predicted with any degree of certainty.

While our ability to predict 2-INPUT circuit performance from SISO data was remarkably accurate, in some cases, we intimated that the properties of the circuit were responsible for increased variability in the performance of a given state of a circuit. Namely, we posited that the variable 5′-UTR in PROXIMAL circuits contributed to decreased accuracy in our predictions—as the CORE circuits had better correlations with said predictions (see Figures 4 and 6). To test this assertion, we inserted a genetic insulator upstream of the putative UTR in a subset of PROXIMAL AND circuits and NOR circuits—*i.e.*, gates with the largest prediction error for each 2-INPUT combinatorial pair—followed by a retest of circuit performance (see Supporting Data Set 4). With the addition of the genetic insulator, we observed an ∼3-fold and ∼6-fold improvement in the accuracy of the prediction of experimental results relative to the model for AND gates and NOR gates, respectively.

An important feature of transcriptional programming is the ability to network repressors and antirepressors to build multiple-INPUT operations that are compressed.^28,30^ In our current study, we accomplished this with the SE-PA architecture to form simple two-node networks of transcription factors binned *via* the alternate DNA binding function—*e.g.*, YQR|O^1^, HQN|O^ttg^, or GKR|O^gac^. This SE-PA network form resulted in seven orthogonal (binned) DNA binding networks, with interbin communication facilitated *via* the INPUT signals (see Supporting Note 10). Given seven nonsynonymous ADR and 5 RCD, each with the capacity to interact with one of five nonsynonymous INPUTs, resulted in a putative network space of 70 operations (*i.e.*, restricted to the given AND gates and NOR gates), with 10^3^ signal coupled operations. When considering mixed unit operations (*i.e.*, said A NIMPLY B gates and B NIMPLY A gates), the network space is represented by 350 putative operations, with signal coupling three times larger than AND gate (or NOR gate) coupled operations.

In the context of network development, the DNA binding network in transcriptional programming can be expanded *via* the SERI architecture. Namely, in a given SERI genetic architecture, two nonsynonymous DNA operators can be paired in tandem—*i.e.*, one located at the CORE position and the other at the PROXIMAL position (see Supporting Figure S4E). When extrapolated based on the engineered transcription factors and cognate DNA operators used in this study, the putative SERI networked DNA space results in 10^3^ operations, with a signal coupling on the order of 10^5^ (see Supporting Note 10).

**Figure 9:**
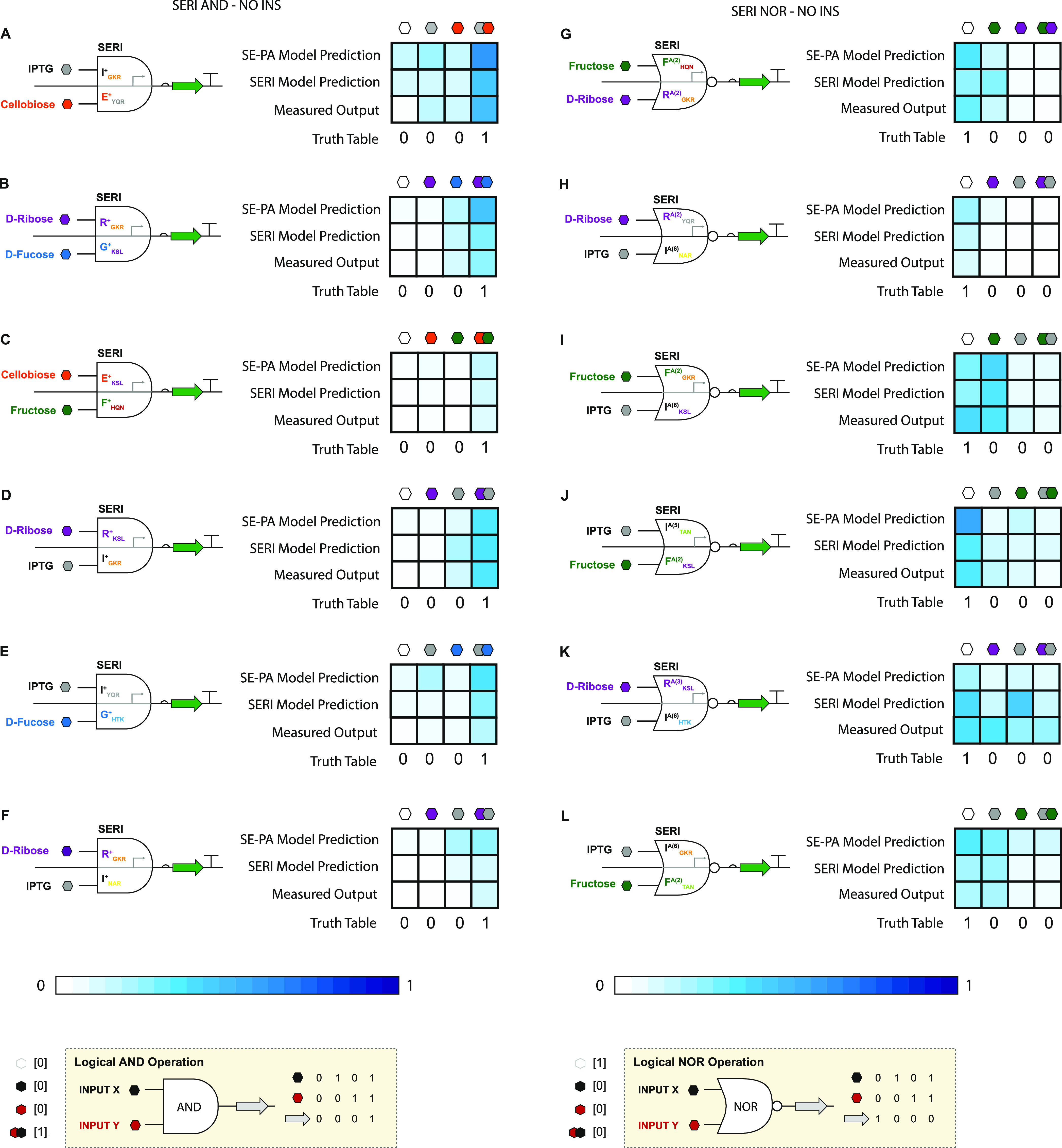
Results for 6 SERI AND operations and 6 SERI NOR operations. (A–F) AND logic gates employing a repressor (X_ADR_^+^) directed to a cognate PROXIMAL operator (top input) and a second repressor (Y_ADR_^+^) directed to a cognate CORE operator (bottom input). Results for OUTPUT prediction using SE-PA SISO parameters, prediction using SERI SISO parameters, and measured OUTPUT are shown on the right. (**G–L**) NOR logic gates employing antirepressors X_ADR_^A^ and Y_ADR_^A^.

To determine if SERI circuits are amenable to modeling (*i.e.*, MISO predictions from SISO data), we leveraged the workflows that we established for SE-PA circuits. Given the enormous combinatorial space for SERI circuits, we opted to demonstrate predictive capacity as a proof-of-concept using a small sample set—*i.e.*, six AND gates (see Figure 9A–F) + six NOR gates (see Figure 9G–L). Congruent with our previous workflows, first, we collected SISO data for each transcription factor—however, in this case, operating in the context of a given SERI operator–promoter (opposed to SE-PA). The rationale for recollecting SISO data is evidenced in the differences in performances between SE-PA and SERI 1-INPUT operations for equivalent transcription factors and cognate DNA interactions. In turn, we built, tested, and modeled the corresponding SERI AND gates (see Supporting Data Set 5). In all cases, the experimental data and model predictions were in good agreement. Likewise, the experimental data and corresponding model of NOR gates were in good agreement; however, many SERI NOR circuits had divergent performance relative to synonymous SE-PA circuits. For example, R_KSL_^A(3)^ and I_HTK_^A(6)^, in principle, should form an objective NOR gate based on the SE-PA SISO data. However, in the context of the SERI architecture, said operations are incompatible and, as predicted, result in a nonfunctional operation (see Figure 9K). Moreover, the inclusion of a genetic insulator does not improve circuit fidelity, indicating that properties and functions that precede translation (*e.g.*, changes in transcription factor DNA interactions and possibly changes in promoter strength) impact the performance of the SERI circuit; see Supporting Figure S10.

## Materials and Methods

### BUFFER and NOT Plasmids

Each SISO system is comprised of (1) a single transcription factor expressed on the pLacI plasmid (Novagen), which contains the p15a origin (copy number 20–30/cell), and (2) a super folder green fluorescent protein (GFP) reporter expressed on the pZS*22-sfGFP plasmid which contains the pSC101 origin (copy number 3–5/cell). Chloramphenicol and kanamycin resistance genes were used as selection markers for transcription factor and reporter plasmids, respectively. The transcription factor and reporter plasmids were obtained from previous works (Rondon et al., Groseclose et al.) and when necessary, ADR or operator variants were cloned using site-directed mutagenesis PCR (Phusion DNA Polymerase, NEB) with custom primers (Eurofins Genomics), followed by kinase, ligase, and *Dpn*I reactions (KLD enzyme mix, NEB). The reactions were transformed into chemically competent DH5α cells (huA2 Δ(argF-lacZ)U169 phoA glnV44 φ80Δ(lacZ)M15 gyrA96 recA1 relA1 endA1 thi-1 hsdR17; New England Biolabs) and plated on LB agar with an appropriate antibiotic. A transformant was cultured overnight in LB broth (Fisher BioReagents) and mini-prepped (Omega Bio-Tek) to yield each plasmid, and the sequence was confirmed with DNA sequencing (Eurofins Genomics).

### AND, NOR, and NIMPLY Transcription Factor Plasmids

Transcription factor plasmids used in MISO systems are identical to those in SISO systems, except they contain two independently driven transcription factor genes. AND, NOR, and NIMPLY transcription factor plasmids were cloned using a modular Golden Gate Assembly method. Transcription factor inserts were PCR amplified (Q5 DNA Polymerase, NEB) using BUFFER and NOT plasmids as templates, gel extracted (Qiagen), and desired pairs were matched and assembled with BsmBI-v2 and T4 DNA ligase (BsmBI-v2 Golden Gate Assembly Kit, NEB). The resulting plasmids were transformed and isolated according to the methods described above.

### Microwell Plate Assay

For each logic gate, the transcription factor plasmid contains a single repressor (BUFFER), single antirepressor (NOT), repressor pair (AND), antirepressor pair (NOR), or repressor/antirepressor pair (NIMPLY). The transcription factor and corresponding reporter plasmids were double-transformed into homemade chemically competent 3.32 *E. coli* cells (Genotype lacZ13(Oc), lacI22, LAM–, el4–, relA1, spoT1, and thiE1, Yale CGSC #5237) and transformants were precultured for 6 hours in LB media with chloramphenicol (25 μg/mL, VWR Life Sciences) and kanamycin (35 μg/mL, VWR Life Sciences) antibiotics. Precultures were then diluted in sextuplicate into glucose (100 mM, Fisher Scientific) M9 minimal media supplemented with 0.2% (w/v) casamino acids (VWR Life Sciences), 1 mM thiamine HCl (Alfa Aesar), antibiotics, and respective inducers, and grown in a flat bottom 96-well microplate (Costar) for 16 hours (37 °C, 300 rpm). Microwell plates were sealed with Breathe-Easy membranes (Diversified Biotech) to prevent evaporation. Inducer concentrations used are as follows: isopropyl-β-D-thiogalactoside (IPTG; 10 mM, reduced to 1 mM for IPTG-fucose gates), D-ribose (10 mM), cellobiose (10 mM), D-fucose (10 mM), fructose (10 mM), and adenine (1 mM). Optical density (OD_600_) and GFP fluorescence (λ_ex_ = 485 nm, λ_em_ = 510 nm) were measured with a Spectramax M2e plate reader (Molecular Devices).

### Data and Statistical Analysis

Raw OD_600_ and GFP fluorescence measurements were corrected by subtracting values of blank media from sample values, and fluorescence values were normalized to OD_600_ in Excel (Microsoft). Mean fluorescence and standard deviation were calculated across the six replicates (*n* = 6) and then normalized to a global maximum of 75,000 relative fluorescence units (RFU), generating a scale from 0 to 1. A two-tailed *t*-test was used to determine statistical significance between ON and OFF states for each BUFFER and NOT gate (significance level = 0.001). Gates with a *p*-value > 0.001 were regarded as not significant and classified as either nonfunctional (X^–^) or super-repressor (X^S^) phenotypes, depending on the expression level. Gates with a *p*-value < 0.001 were regarded as functional with either repressor (X^+^) or antirepressor (X^A^) phenotype classification. Cohen’s *d*-values were also calculated to determine the effect size between the two means. For AND, NOR, and NIMPLY gates, a one-way ANOVA test was performed across the four inducer conditions, followed by a Tukey–Kramer test to determine which treatments were statistically different from each other in each gate. We used a *p*-value cutoff of < 0.01 for significance (see Supporting Data Set 2–5).

### AND, NOR, and NIMPLY Gate Prediction

Compatibility tests and OUTPUT predictions for AND, NOR, and NIMPLY gates were performed in Excel using the appropriate models and experimental BUFFER and NOT data (see Supplemental Data). First, σ and ε values were calculated using normalized ON and OFF state OUTPUTs. Compatibility tests were performed with a true/false assessment of each inequality (see Supporting Data Set 2). Model parameters α_0_, α_1_, α_2_, and α_3_ were then evaluated using σ and ε values and plugged into the respective model equation for each 2-INPUT gate. Prediction values were calculated for each inducer condition and plotted against experimental data using GraphPad (Prism) for correlation analysis. The prediction error was calculated for each INPUT condition across all gates as the ratio of the measured OUTPUT to the predicted OUTPUT.

### Insulated SE-PA and SERI Logic Gates

For each proximal AND and NOR RCD pair, the ADR variant with the largest mean prediction error (determined as the magnitude of fold change, averaged across all four INPUT conditions) was selected for the insulated genetic architecture case study. Insulated reporters were cloned using site-directed mutagenesis PCR (using a template O^agg^ core O^ttg^ proximal RiboJ10 GFP reporter provided by Groseclose et al.). This reaction was performed with a Q5 High-Fidelity DNA Polymerase (NEB), the product was verified with gel electrophoresis, and the amplicon was circularized using kinase, ligase, and Dpn1 enzymes (KLD). The reactions were transformed into chemically competent DH5α cells and plated on LB agar supplemented with kanamycin. Transformants were isolated, cultured overnight, and mini-prepped to yield each plasmid and the sequence was confirmed with DNA sequencing. The transcription factor and insulated reporter plasmids were double-transformed and both SISO (BUFFER or NOT) and MISO (AND or NOR) operations were constructed and assayed, as described previously. For SERI gates, core operators were inserted with site-directed mutagenesis PCR (using proximal SE-PA reporters as templates) according to the KLD method described above, and both SISO and MISO operations were constructed and assayed according to the procedure above.

## Supporting Information Available

Expanded models and discussion for AND, NOR, A NIMPLY B, and B NIMPLY A; PROXIMAL/CORE BUFFER and NOT gate performance cards; compatible and incompatible AND/NOR gate components; histograms of the prediction error; PROXIMAL and CORE SE-PA NIMPLY logic; results for insulated SERI AND gates and NOR gates (PDF)
Metrological analysis on and modeling of said transcription factors (XLSX)
AND gates were functional with qualitative (objective) performances congruent with the intended logical operation (XLSX)
On average, objective (qualitative) NOR gating was not observed or had poor performance (XLSX)
Gates with the largest prediction error for each 2-INPUT combinatorial pair, followed by a retest of circuit performance (XLSX)
In turn, we built, tested, and modeled the corresponding SERI AND gates (XLSX)
