# Supplementary material for: Performance Prediction of Fundamental Transcriptional Programs
Source: ACS Synth Biol. 2023 Mar 20;12(4):1094–108. doi: 10.1021/acssynbio.2c00593 (PMC10127286; doi:10.1021/acssynbio.2c00593)
Supplement: Supplementary file 1 — sb2c00593_si_001.pdf [file sb2c00593_si_001.pdf]

Supporting Information

Performance Prediction of Fundamental Transcriptional Programs

Prasaad T. Milner[1], Ziqiao Zhang[2], Zachary D. Herde[1], Namratha R. Vedire[3], Fumin Zhang[2], Matthew J. Realff[1], and Corey J. Wilson[1†]

[1]Georgia Institute of Technology, School of Chemical & Biomolecular Engineering
[2]Georgia Institute of Technology, School of Electrical and Computer Engineering
[3]Georgia Institute of Technology, School of Computer Science

[†]To whom correspondence should be addressed: Corey J. Wilson, Georgia Institute of Technology, School of Chemical & Biomolecular Engineering, 311 Ferst Drive, Atlanta, GA 30332-0100.
E-Mail: corey.wilson@chbe.gatech.edu

Table of Contents

Supplementary Note 1. Anti-repression versus Inversion.

Supplementary Note 2. Design Workflow for 2-INPUT Operations.

Supplementary Note 3. Engineered Transcription Factors *via* Modular Design.

Supplementary Note 4. Transcriptional Programming.

Supplementary Note 5. Metrics for Engineered X^{+}_{ADR} Repressors and X^{A}_{ADR} Anti-repressors.

Supplementary Note 6. AND Logic Model.

Supplementary Note 7. NOR Logic Model.

Supplementary Note 8. A NIMPLY B Logic Model.

Supplementary Note 9. B NIMPLY A Logic Model.

Supplementary Note 10. Determination of Possible Combinations for Logic Gates.

Supplementary Figure S1. PROXIMAL BUFFER and NOT Gate Performance Cards.

Supplementary Figure S2. CORE BUFFER and NOT Gate Performance Cards.

Supplementary Figure S3. Operational and Non-operational SISO Logic Gates.

Supplementary Figure S4. Genetic Architectures.

Supplementary Figure S5. Compatible and Incompatible AND Gate Components.

Supplementary Figure S6. Histograms of Prediction Error.

Supplementary Figure S7. Compatible and Incompatible NOR Gate Components.

Supplementary Figure S8. PROXIMAL and CORE SE-PA NIMPLY Logic.

Supplementary Figure S9. Results for PROXIMAL SE-PA NIMPLY Logic.

Supplementary Figure S10. Results for Insulated SERI AND Gates and NOR Gates.

Supplementary Note 1. Anti-repression versus Inversion. Inversion is a process in which a single repressor is expressed on one layer and is directed to interact with a cognate DNA element to reject an OUTPUT located on a second layer, and can be regarded as a NOT operation – notably, Cello circuits are constructed *via* the said inversion process [1]. In contrast, anti-repressors reduce the NOT operation to a single layer and single promoter and the reduction in components (*e.g.*, promoters) is defined as circuit compression, see **Figure 1B** and **Supplementary Figure S3B**.

Supplementary Note 2. Design Workflow for 2-INPUT Operations. To construct single layer 2-INPUT operations from 1-INPUT operations requires the use of engineered transcription factors and engineered cognate genetic architectures, see **Figure 1**. Engineered transcription factors are developed *via* modular design (see **Supplementary Note 3**) which enables the development of synonymous DNA binding functions for two transcription factors that process two different INPUTs. In turn, coupled DNA functions between two engineered transcription factors can be directed *via* a SE-PA or SERI genetic architecture (see **Supplementary Figure S4**) to facilitate the construction of a 2-INPUT operation.

Supplementary Note 3. Engineered Transcription Factors *via* Modular Design. The design template LacI is part of a large family of proteins that share a topology and putative mechanism of action [2]. The LacI/GalR protein family is made up of over 1,000 homologues. Moreover, the LacI/GalR transcription regulatory proteins mediate responses to a wide range of environmental and metabolic changes. Structurally, the general LacI/GalR topology can be defined by two fundamental domains – *i.e.*, (i) a regulatory core domain, and (ii) a DNA binding domain. Accordingly, we can regard this collection of paralogues as a putative design space – when carefully decomposed – positing that said functional domains can be mixed and matched to form new allosteric transcription factors.

Supplementary Note 4. Transcriptional Programming. Transcriptional programming is predicated on a definitive bottom-up combinational rule set. Single-input single-output operations (BUFFER and NOT) represent the fundamental binaries, that can be systematically combined to create all proper two-input single-output operations. Complex circuit development *via* transcriptional programming (*e.g.*, OR, NAND, A IMPLY B, B IMPLY A, XOR, and XNOR) involve feeding forward information [3] – which is beyond the scope of the current manuscript.

Supplementary Note 5:

Metrics for Engineered X^{+}_{ADR} Repressors. The Fraction of Maximum Output (F.M.O.) ≡ [GFP/OD600] / [Max LacSTOP value**], where (i) F.M.O. Repression is the system minus ligand, and (ii) F.M.O. Induction is the system plus ligand. Repression Strength ≡ F.M.O. normalized output minus ligand, and Fold Induction ≡ FI, such that:

$$\mathrm{FI} = \frac{(\mathrm{F.M.O.\ Induction})}{(\mathrm{F.M.O.\ Repression})} \qquad \textbf{(1)}$$

Part 1 of the traceability score is given in terms of the induced state (where IU = Induction Units) such that the IU traceability scores were calculated as follows:

$$\mathrm{IU\ Traceability\ Score} = \frac{\mathrm{FI}(\mathrm{X}^{+}_{\mathrm{ADR}}|\mathrm{OP})}{\mathrm{FI}(\mathrm{I}^{+}_{\mathrm{YQR}}|\mathrm{O^1}_{\mathrm{prox}})} \qquad \textbf{(2)}$$

$$\mathrm{IU\ Reference\ Score} = \frac{\mathrm{FI}(\mathrm{I}^{+}_{\mathrm{YQR}}|\mathrm{O^1}_{\mathrm{prox}})}{\mathrm{FI}(\mathrm{I}^{+}_{\mathrm{YQR}}|\mathrm{O^1}_{\mathrm{prox}})} = 1 \qquad \textbf{(3)}$$

Part 2 of the traceability score is given in terms of the repressed state (where RU = Repression Units) such that the RU traceability scores were calculated as follows:

$$\text{RU Traceability Score} = \frac{\text{F.M.O. Repression } (X^{+}_{ADR}|OP)}{\text{F.M.O. Repression } (I^{+}_{YQR}|{O^{1}}_{prox})} \quad \textbf{(4)}$$

$$\text{RU Reference Score} = \frac{\text{F.M.O. Repression } (I^{+}_{YQR}|{O^{1}}_{prox})}{\text{F.M.O. Repression } (I^{+}_{YQR}|{O^{1}}_{prox})} = 1 \quad \textbf{(5)}$$

**Max LacSTOP value = 75,000 relative fluorescence units (rfu), OD_{600} normalized

Metrics for Engineered X^{A}_{ADR} Anti-repressors. The Fraction of Maximum Output (F.M.O.) ≡ [GFP/OD_{600}] / [Max LacSTOP value**], where (i) F.M.O. anti-repression is the system minus ligand, and (ii) F.M.O. anti-induction is the system plus ligand. Fold Anti-induction ≡ FAI, such that:

$$\text{FAI} = \frac{(\text{F.M.O. Anti} - \text{repression})}{(\text{F.M.O. Anti} - \text{induction})} \quad \textbf{(6)}$$

Part 1 of the traceability score is given in terms of the anti-repressed state (where AIU = Anti-Induction Units) such that the AIU traceability scores were calculated as follows:

$$\text{AIU Traceability Score} = \frac{\text{FAI}(X^{A}_{ADR}|OP)}{\text{FI}(I^{+}_{YQR}|{O^{1}}_{prox})} \quad \textbf{(7)}$$

$$\text{AIU Reference Score} = \frac{\text{FI}(I^{+}_{YQR}|{O^{1}}_{prox})}{\text{FI}(I^{+}_{YQR}|{O^{1}}_{prox})} = 1 \quad \textbf{(8)}$$

Part 2 of the traceability score is given in terms of the repressed state (where RU = Repression Units) such that the RU traceability scores for an anti-repressor were calculated as follows:

$$\text{RU Traceability Score} = \frac{\text{F.M.O. Anti} - \text{repression } (X^{A}_{ADR}|OP)}{\text{F.M.O. Repression } (I^{+}_{YQR}|{O^{1}}_{prox})} \quad \textbf{(9)}$$

$$\text{RU Reference Score} = \frac{\text{F.M.O. Repression } (I^{+}_{YQR}|{O^{1}}_{prox})}{\text{F.M.O. Repression } (I^{+}_{YQR}|{O^{1}}_{prox})} = 1 \quad \textbf{(10)}$$

**Max LacSTOP value = 75,000 relative fluorescence units (rfu), OD_{600} normalized

Supplementary Note 6:

AND logic model. To better interpret and predict the qualitative performance of our 2-INPUT AND logic gates, we constructed a course-grained model – defined as follows

$$\Omega_{AND}(I_X,I_Y) = \alpha_o + \alpha_1\Lambda^+_X(I_x) + \alpha_2\Lambda^+_Y(I_Y) + \alpha_3\Lambda^+_X(I_X)\,\Lambda^+_Y(I_Y) \quad \textbf{(11)}$$

Where Ω_{AND} is the OUTPUT expression, Λ^+_X is the Hill state function of repressor X^+, Λ^+_Y is the Hill state function of repressor Y^+, I_X is the inducer state of X^+ (either 0 or 1), I_Y is the inducer state of Y^+ (either 0 or 1), and α_o, α_1, α_2, and α_3 are parameters determined by a set of four equations:

$$\alpha_o = \min(\varepsilon_X, \varepsilon_Y) \quad \textbf{(12)}$$
$$\alpha_o + \alpha_1 = \varepsilon_Y \quad \textbf{(13)}$$
$$\alpha_o + \alpha_2 = \varepsilon_X \quad \textbf{(14)}$$
$$\alpha_o + \alpha_1 + \alpha_2 + \alpha_3 = \min(\sigma_X + \varepsilon_X, \sigma_Y + \varepsilon_Y) \quad \textbf{(15)}$$

Qualitatively, α_o is the minimum OUTPUT of the gate (or overall leakiness, *i.e.*, ε_X or ε_Y), α_1 is the OUTPUT increase (from the baseline α_o) in response to I_X, α_2 is the OUTPUT increase (from the baseline α_o) in response to I_Y, and α_3 is the OUTPUT increase from the maximum OFF state to the ON state.

Equations 12, 13, 14, and **15** are derived from solving **Equation 11** using three distinct assumptions. First, we assume that when *neither* I_X or I_Y is present, the TF with the lowest SISO OFF state OUTPUT controls Ω_{AND}. This is represented as

$$\Omega_{AND}(0,0) = \min(\Omega^+_{BUFR,X}(0), \Omega^+_{BUFR,Y}(0))$$
$$\alpha_o + \alpha_1(0) + \alpha_2(0) + \alpha_3(0)(0) = \min(\sigma_X(0)+ \varepsilon_X, \sigma_Y(0) + \varepsilon_Y)$$
$$\alpha_o = \min(\varepsilon_X, \varepsilon_Y)$$

Second, we assume that when *either* I_X or I_Y is present, the TF in the OFF-state controls Ω_{AND}.

$$\Omega_{AND}(1,0) = \Omega^+_{BUFR,Y}(0)$$
$$\alpha_o + \alpha_1(1) + \alpha_2(0) + \alpha_3(1)(0) = \sigma_Y(0) + \varepsilon_Y$$
$$\alpha_o + \alpha_1 = \varepsilon_Y$$

$$\Omega_{AND}(0,1) = \Omega^+_{BUFR,X}(0)$$
$$\alpha_o + \alpha_1(0) + \alpha_2(1) + \alpha_3(0)(1) = \sigma_X(0) + \varepsilon_X$$
$$\alpha_o + \alpha_2 = \varepsilon_X$$

Finally, we assume that when *both* I_X and I_Y are present, Ω_{AND} is given by the TF with the lowest ON state OUTPUT.

$$\Omega_{AND}(1,1) = \min(\Omega^+_{BUFR,X}(1), \Omega^+_{BUFR,Y}(1))$$
$$\alpha_o + \alpha_1(1) + \alpha_2(1) + \alpha_3(1)(1) = \min(\sigma_X(1)+ \varepsilon_X, \sigma_Y(1) + \varepsilon_Y)$$
$$\alpha_o + \alpha_1 + \alpha_2 + \alpha_3 = \min(\sigma_X + \varepsilon_X, \sigma_Y + \varepsilon_Y)$$

Summary of Outcomes: The model could accurately predict the qualitative performance of all measured values in the context of the AND model in ~88% of cases – *i.e.*, only ~12% of the values had a 2-fold or greater difference relative to the predicted value. An error in AND condition 1 (-,-) correlates to the OFF states of both TF_X and TF_Y, an error in AND condition 2 (-,+) correlates to the OFF state of TF_Y, an error

in AND condition 3 (+,-) correlates to the OFF state of TF_X, and error in AND condition 4 (+,+) correlates to the ON states of both TF_X and TF_Y, also see **Figure 3A**.

Supplementary Note 7:

NOR logic model. For 2-INPUT NOR logic gates, we modified the model shown in Equation 3 to include anti-repressor state functions pertaining to NOT SISO logic. The model for NOR is shown below:

$$\Omega_{NOR}(I_X,I_Y) = \alpha_o + \alpha_1\Lambda^A{}_X(I_x) + \alpha_2\Lambda^A{}_Y(I_Y) + \alpha_3\Lambda^A{}_X(I_X)\,\Lambda^A{}_Y(I_Y) \quad \textbf{(16)}$$

Where Ω_{NOR} is the OUTPUT expression, $\Lambda^A{}_X$ is the Hill state function of anti-repressor X^A, $\Lambda^A{}_Y$ is the Hill state function of anti-repressor Y^A, I_X is the inducer state of X^A (either 0 or 1), I_Y is the inducer state of Y^A (either 0 or 1), and α_o, α_1, α_2, and α_3 are parameters determined by the set of four equations described previously:

$$\alpha_o = \min(\varepsilon_X, \varepsilon_Y) \quad \textbf{(17)}$$
$$\alpha_o + \alpha_1 = \varepsilon_Y \quad \textbf{(18)}$$
$$\alpha_o + \alpha_2 = \varepsilon_X \quad \textbf{(19)}$$
$$\alpha_o + \alpha_1 + \alpha_2 + \alpha_3 = \min(\sigma_X + \varepsilon_X, \sigma_Y + \varepsilon_Y) \quad \textbf{(20)}$$

Equations 17, 18, 19, and **20** are derived again from solving **Equation 16** using the same assumptions described in the AND model, but with antithetical input conditions (due to the antithetical phenotype of anti-repressors from repressors). First, we assume that when *both* I_X and I_Y are present, the TF with the lowest SISO OFF state OUTPUT controls Ω_{NOR}. This is represented as

$$\Omega_{NOR}(1,1) = \min(\Omega^A{}_{NOT,X}(1), \Omega^A{}_{NOT,Y}(1))$$
$$\alpha_o + \alpha_1(0) + \alpha_2(0) + \alpha_3(0)(0) = \min(\sigma_X(0) + \varepsilon_X, \sigma_Y(0) + \varepsilon_Y)$$
$$\alpha_o = \min(\varepsilon_X, \varepsilon_Y)$$

Second, we assume that when *either* I_X or I_Y is present, the TF in the OFF-state controls Ω_{NOR}.

$$\Omega_{NOR}(1,0) = \Omega^+{}_{NOT,X}(1)$$
$$\alpha_o + \alpha_1(0) + \alpha_2(1) + \alpha_3(0)(1) = \sigma_X(0) + \varepsilon_X$$
$$\alpha_o + \alpha_2 = \varepsilon_X$$

$$\Omega_{NOR}(0,1) = \Omega^+{}_{NOT,Y}(1)$$
$$\alpha_o + \alpha_1(1) + \alpha_2(0) + \alpha_3(1)(0) = \sigma_Y(0) + \varepsilon_Y$$
$$\alpha_o + \alpha_1 = \varepsilon_Y$$

Finally, we assume that when *neither* I_X or I_Y are present, Ω_{NOR} is given by the TF with the lowest ON state OUTPUT.

$$\Omega_{NOR}(0,0) = \min(\Omega^A{}_{NOT,X}(0), \Omega^A{}_{NOT,Y}(0))$$
$$\alpha_o + \alpha_1(1) + \alpha_2(1) + \alpha_3(1)(1) = \min(\sigma_X(1) + \varepsilon_X, \sigma_Y(1) + \varepsilon_Y)$$
$$\alpha_o + \alpha_1 + \alpha_2 + \alpha_3 = \min(\sigma_X + \varepsilon_X, \sigma_Y + \varepsilon_Y)$$

Supplementary Note 8:

A NIMPLY B logic model. For 2-INPUT B NIMPLY A logic gates, we modified the model shown in Equation 3 to include one repressor and one anti-repressor state function pertaining to both BUFFER and NOT SISO logic. The model for X NIMPLY Y is shown below:

$$\Omega_{X\ NIMPLY\ Y}(I_X,I_Y) = \alpha_o + \alpha_1\Lambda^{+}{}_X(I_x) + \alpha_2\Lambda^{A}{}_Y(I_Y) + \alpha_3\Lambda^{+}{}_X(I_X)\ \Lambda^{A}{}_Y(I_Y) \quad \textbf{(21)}$$

Where $\Omega_{A\ NIMPLY\ B}$ is the OUTPUT expression, $\Lambda^{+}{}_X$ is the Hill state function of repressor X^+, $\Lambda^{A}{}_Y$ is the Hill state function of anti-repressor Y^A, I_X is the inducer state of X^+ (either 0 or 1), I_Y is the inducer state of Y^A (either 0 or 1), and α_o, α_1, α_2, and α_3 are parameters determined by the set of four equations described previously:

$$\alpha_o = \min(\varepsilon_X, \varepsilon_Y) \quad \textbf{(22)}$$
$$\alpha_o + \alpha_1 = \varepsilon_Y \quad \textbf{(23)}$$
$$\alpha_o + \alpha_2 = \varepsilon_X \quad \textbf{(24)}$$
$$\alpha_o + \alpha_1 + \alpha_2 + \alpha_3 = \min(\sigma_X + \varepsilon_X, \sigma_Y + \varepsilon_Y) \quad \textbf{(25)}$$

Equations 22, 23, 24, and **25** are derived again from solving **Equation 21** using the same assumptions described previously, but with input conditions reflecting the phenotypes of each TF. First, we assume that when *only* I_Y is present, the TF with the lowest SISO OFF state OUTPUT controls $\Omega_{A\ NIMPLY\ B}$. This is represented as

$$\Omega_{A\ NIMPLY\ B}(0,1) = \min(\Omega^{+}{}_{BUFR,X}(0), \Omega^{A}{}_{NOT,Y}(1))$$
$$\alpha_o + \alpha_1(0) + \alpha_2(0) + \alpha_3(0)(0) = \min(\sigma_X(0) + \varepsilon_X, \sigma_Y(0) + \varepsilon_Y)$$
$$\alpha_o = \min(\varepsilon_X, \varepsilon_Y)$$

Second, we assume that when *neither* I_X or I_Y is present, the repressor X^+ controls $\Omega_{X\ NIMPLY\ Y}$.

$$\Omega_{A\ NIMPLY\ B}(0,0) = \Omega^{+}{}_{BUFR,X}(0)$$
$$\alpha_o + \alpha_1(0) + \alpha_2(1) + \alpha_3(0)(1) = \sigma_X(0) + \varepsilon_X$$
$$\alpha_o + \alpha_2 = \varepsilon_X$$

Similarly, when *both* I_X and I_Y are present, the anti-repressor Y^A controls $\Omega_{X\ NIMPLY\ Y}$.

$$\Omega_{A\ NIMPLY\ B}(1,1) = \Omega^{A}{}_{NOT,Y}(0)$$
$$\alpha_o + \alpha_1(1) + \alpha_2(0) + \alpha_3(1)(0) = \sigma_Y(0) + \varepsilon_Y$$
$$\alpha_o + \alpha_1 = \varepsilon_Y$$

Finally, we assume that when *only* I_X is present, $\Omega_{A\ NIMPLY\ B}$ is given by the TF with the lowest ON state OUTPUT.

$$\Omega_{A\ NIMPLY\ B}(1,0) = \min(\Omega^{+}{}_{BUFR,X}(1), \Omega^{+}{}_{BUFR,Y}(0))$$
$$\alpha_o + \alpha_1(1) + \alpha_2(1) + \alpha_3(1)(1) = \min(\sigma_X(1) + \varepsilon_X, \sigma_Y(1) + \varepsilon_Y)$$
$$\alpha_o + \alpha_1 + \alpha_2 + \alpha_3 = \min(\sigma_X + \varepsilon_X, \sigma_Y + \varepsilon_Y)$$

Supplementary Note 9:

B NIMPLY A logic model. The model for 2-INPUT B NIMPLY A logic gates follows that described above for A NIMPLY B gates, with the modification that TFs X and Y phenotypes are switched, so that this system contains an anti-repressor X^A and repressor Y^+. The model is therefore:

$$\Omega_{B\ NIMPLY\ A}(I_X,I_Y) = \alpha_o + \alpha_1\Lambda^A{}_X(I_x) + \alpha_2\Lambda^+{}_Y(I_Y) + \alpha_3\Lambda^A{}_X(I_X)\ \Lambda^+{}_Y(I_Y) \quad \textbf{(26)}$$

Where $\Omega_{B\ NIMPLY\ A}$ is the OUTPUT expression, $\Lambda^A{}_X$ is the Hill state function of anti-repressor X^A, $\Lambda^+{}_Y$ is the Hill state function of repressor Y^+, I_X is the inducer state of X^A (either 0 or 1), I_Y is the inducer state of Y^+ (either 0 or 1), and α_o, α_1, α_2, and α_3 are parameters determined by the set of four equations described previously:

$$\alpha_o = \min(\varepsilon_X, \varepsilon_Y) \quad \textbf{(27)}$$
$$\alpha_o + \alpha_1 = \varepsilon_Y \quad \textbf{(28)}$$
$$\alpha_o + \alpha_2 = \varepsilon_X \quad \textbf{(29)}$$
$$\alpha_o + \alpha_1 + \alpha_2 + \alpha_3 = \min(\sigma_X + \varepsilon_X, \sigma_Y + \varepsilon_Y) \quad \textbf{(30)}$$

Equations 27, 28, 29, and **30** are derived again from solving **Equation 26** using the same assumptions described previously, but with input conditions reflecting the phenotypes of each TF. First, we assume that when *only* I_X is present, the TF with the lowest SISO OFF state OUTPUT controls $\Omega_{B\ NIMPLY\ A}$. This is represented as

$$\Omega_{B\ NIMPLY\ A}(1,0) = \min(\Omega^A{}_{NOT,X}(1), \Omega^+{}_{BUFR,Y}(0))$$
$$\alpha_o + \alpha_1(0) + \alpha_2(0) + \alpha_3(0)(0) = \min(\sigma_X(0)+ \varepsilon_X, \sigma_Y(0) + \varepsilon_Y)$$
$$\alpha_o = \min(\varepsilon_X, \varepsilon_Y)$$

Second, we assume that when *neither* I_X or I_Y is present, the repressor Y^+ controls $\Omega_{B\ NIMPLY\ A}$.

$$\Omega_{B\ NIMPLY\ A}(0,0) = \Omega^+{}_{BUFR,Y}(0)$$
$$\alpha_o + \alpha_1(1) + \alpha_2(0) + \alpha_3(1)(0) = \sigma_Y(0) + \varepsilon_Y$$
$$\alpha_o + \alpha_1 = \varepsilon_Y$$

Similarly, when *both* I_X and I_Y are present, the anti-repressor X^A controls $\Omega_{B\ NIMPLY\ A}$.

$$\Omega_{B\ NIMPLY\ A}(1,1)= \Omega^A{}_{NOT,X}(0)$$
$$\alpha_o + \alpha_1(0) + \alpha_2(1) + \alpha_3(0)(1) = \sigma_X(0) + \varepsilon_X$$
$$\alpha_o + \alpha_2 = \varepsilon_X$$

Finally, we assume that when *only* I_Y is present, $\Omega_{B\ NIMPLY\ A}$ is given by the TF with the lowest ON state OUTPUT.

$$\Omega_{B\ NIMPLY\ A}(0,1) = \min(\Omega^A{}_{NOT,X}(0), \Omega^+{}_{BUFR,Y}(1))$$
$$\alpha_o + \alpha_1(1) + \alpha_2(1) + \alpha_3(1)(1) = \min(\sigma_X(1)+ \varepsilon_X, \sigma_Y(1) + \varepsilon_Y)$$
$$\alpha_o + \alpha_1 + \alpha_2 + \alpha_3 = \min(\sigma_X + \varepsilon_X, \sigma_Y + \varepsilon_Y)$$

Supplementary Note 10: The number of possible SE-PA logical BUFFER or NOT SISO operations can be calculated from simple combinations of the selection of an ADR (1 of 5 either repressor [AND] or anti-repressor [NOT]) and DBD (1 of 8). This combination can be placed in either the PROXIMAL or CORE position leading to: ${}^{5}_{1}C \times {}^{8}_{1}C = 40$ SISO operations per position or 80 in total for either the BUFFER or NOT. It should be noted that two of the ADRs O^1 and O^{sym} recognize the same DNA binding domain and so are synonymous, which leads to an effective design space of 70 SISO operations.

For the SE-PA operation of AND, we need to select two ADR (2 of 5) but only one DBD to which each ADR will be coupled. Again, we can place this SE-PA AND operator in either the PROXIMAL or CORE position: ${}^{5}_{2}C \times {}^{8}_{1}C \times 2 = 160$ possible SE-PA AND gates, or 140 non-synonymous combinations. A similar argument holds for the NOR gates where we again select the same numbers of components but from the set of DBDs with anti-repressor behavior, leading to 160 possible NOR gates.

For the SE-PA operation of NIMPLY, we need to select 1 ADR from the set of repressors and 1 ADR from the set of anti-repressors, and one DBD. In general, this would lead to ${}^{5}_{1}C \times {}^{5}_{1}C \times {}^{7}_{1}C = 175$ possible non-synonymous NIMPLY designs per position for a total of 350 possible designs. However, four of the five signals are the same for both the repressor and anti-repressor (Cellobiose and Adenine being the two different ones respectively). This means that except for these two, when the signal is selected for the first operator the second one can only be selected from the remaining four. This leads to (5+4x4)x8 or (5+4x4)x7 NIMPLY designs for each position, 168 or 147 respectively.

For the SERI architecture for the two-input one output AND gate we must select two non-synonymous ADRs and these are placed uniquely in the PROXIMAL and CORE positions and we can select two DBD's to be coupled to these ADRs: ${}^{5}_{2}C \times {}^{7}_{2}C = 210$. For the NIMPLY logical operation the combinations are similar with one repressor and one anti-repressor being selected from each set and each one coupled to an ADR ${}^{5}_{1}C \times {}^{5}_{1}C \times {}^{7}_{2}C = 575$. Both of these designs for SERI architecture can be doubled in number because the ordering of the two ADR's in the PROXIMAL and CORE positions can be switched. However, for this specific system, the same argument about the signal overlap applies. Hence the number of unique combinations of the DBD's is reduced to 21 leading to $21 \times {}^{7}_{2}C = 441$ logical operations that can be doubled in number by changing the order of the ADR's in the PROXIMAL and CORE positions.

Genetic Architecture	AND Operations	NOR Operations	A NIMPLY B Operations	B NIMPLY A Operations
**SE-PA**	${}^{5}_{2}C \times {}^{7}_{1}C = 70$	${}^{5}_{2}C \times {}^{7}_{1}C = 70$	${}^{6}_{2}C \times {}^{7}_{1}C = 105$	${}^{6}_{2}C \times {}^{7}_{1}C = 105$
**SERI**	${}^{5}_{2}C \times {}^{7}_{2}C = 210$	${}^{5}_{2}C \times {}^{7}_{2}C = 210$	${}^{6}_{2}C \times {}^{7}_{2}C = 315$	${}^{6}_{2}C \times {}^{7}_{2}C = 315$

Genetic Architecture	Signal coupled AND Operations	Signal coupled NOR Operations	Signal coupled A NIMPLY B Operations	Signal coupled B NIMPLY A Operations
**SE-PA**	${}^{5}_{1}C \times {}^{(7x4)}_{2}C = 1890$	${}^{5}_{1}C \times {}^{(7x4)}_{2}C = 1890$	${}^{6}_{1}C \times {}^{(7x5)}_{2}C = 3570$	${}^{6}_{1}C \times {}^{(7x5)}_{2}C = 3570$
**SERI**	${}^{5}_{1}C \times {}^{(21x4)}_{2}C = 17{,}430$	${}^{5}_{1}C \times {}^{(21x4)}_{2}C = 17{,}430$	${}^{6}_{1}C \times {}^{(21x5)}_{2}C = 32{,}760$	${}^{6}_{1}C \times {}^{(21x5)}_{2}C = 32{,}760$

Supplementary Figure S1 – Part 1

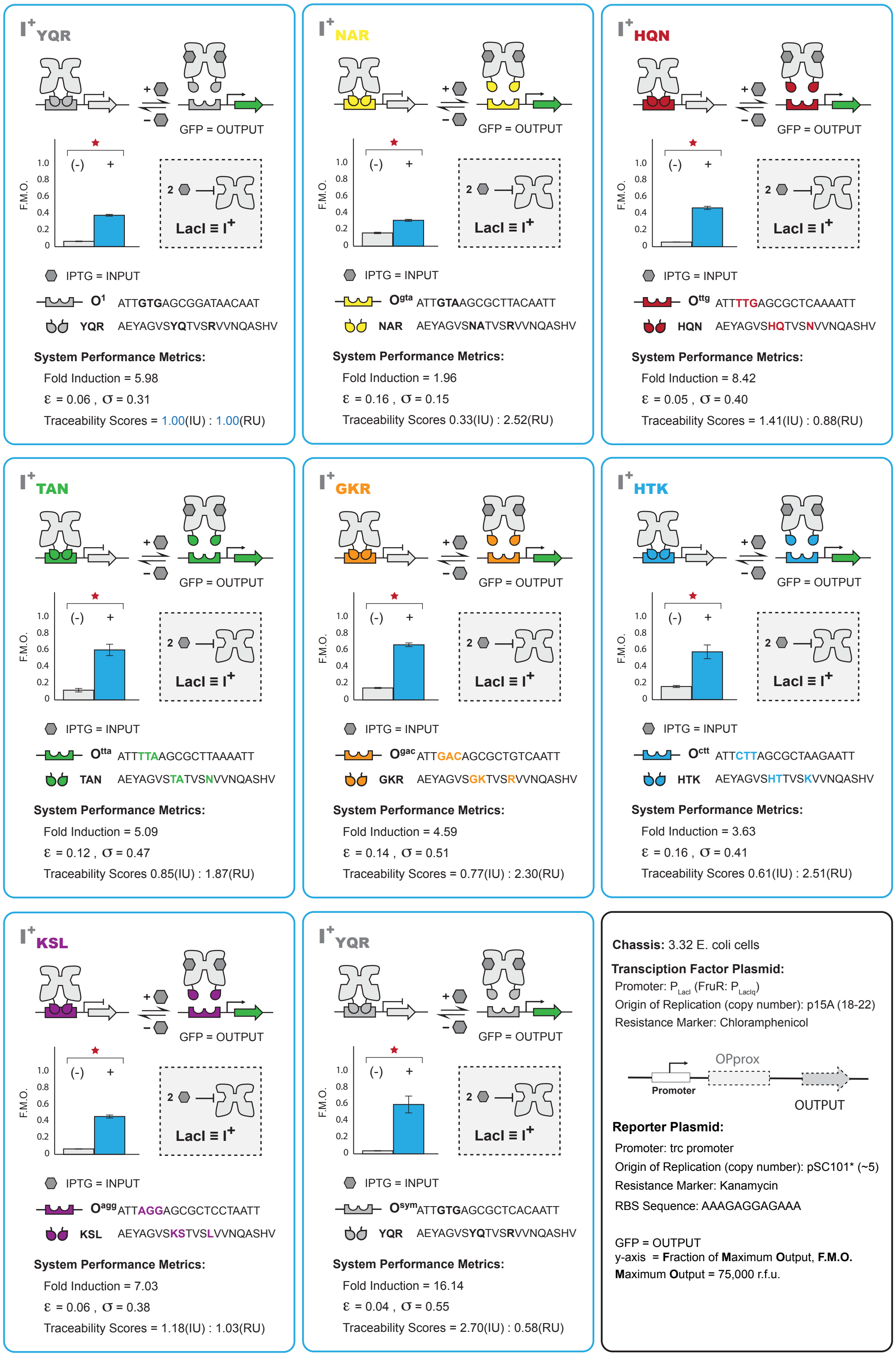

Supplementary Figure S1 – Part 2

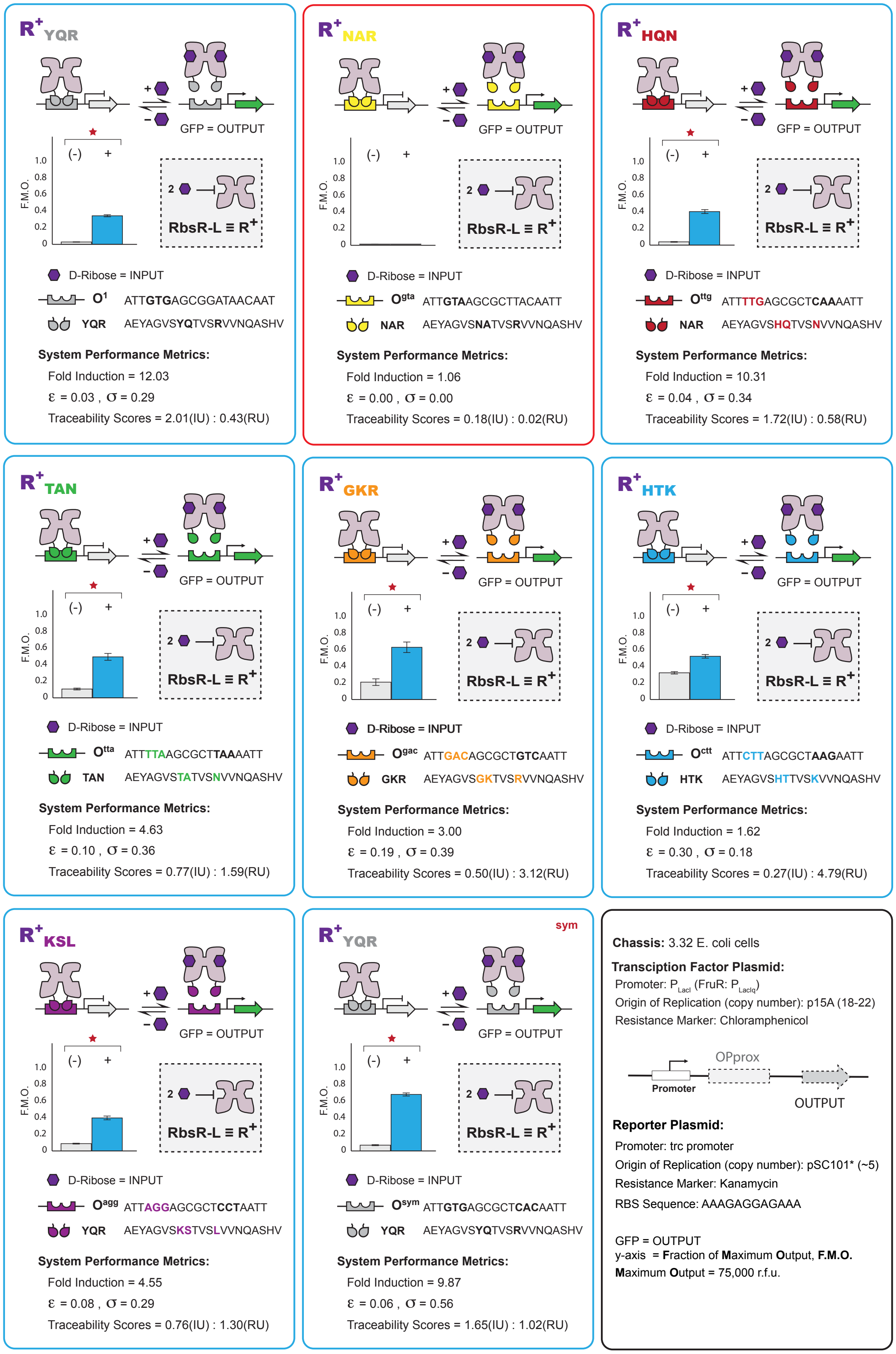

Supplementary Figure S1 – Part 3

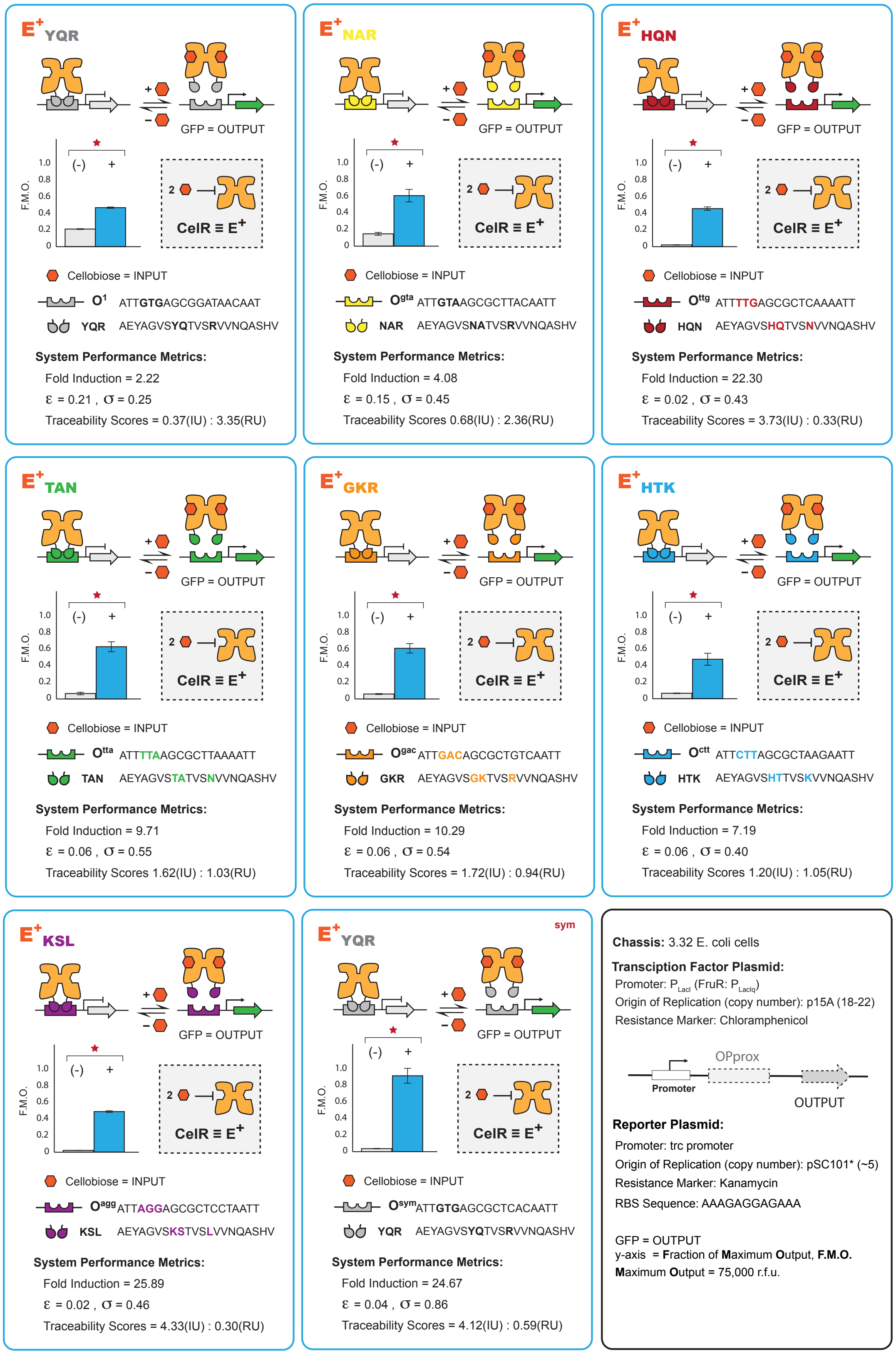

Supplementary Figure S1 – Part 4

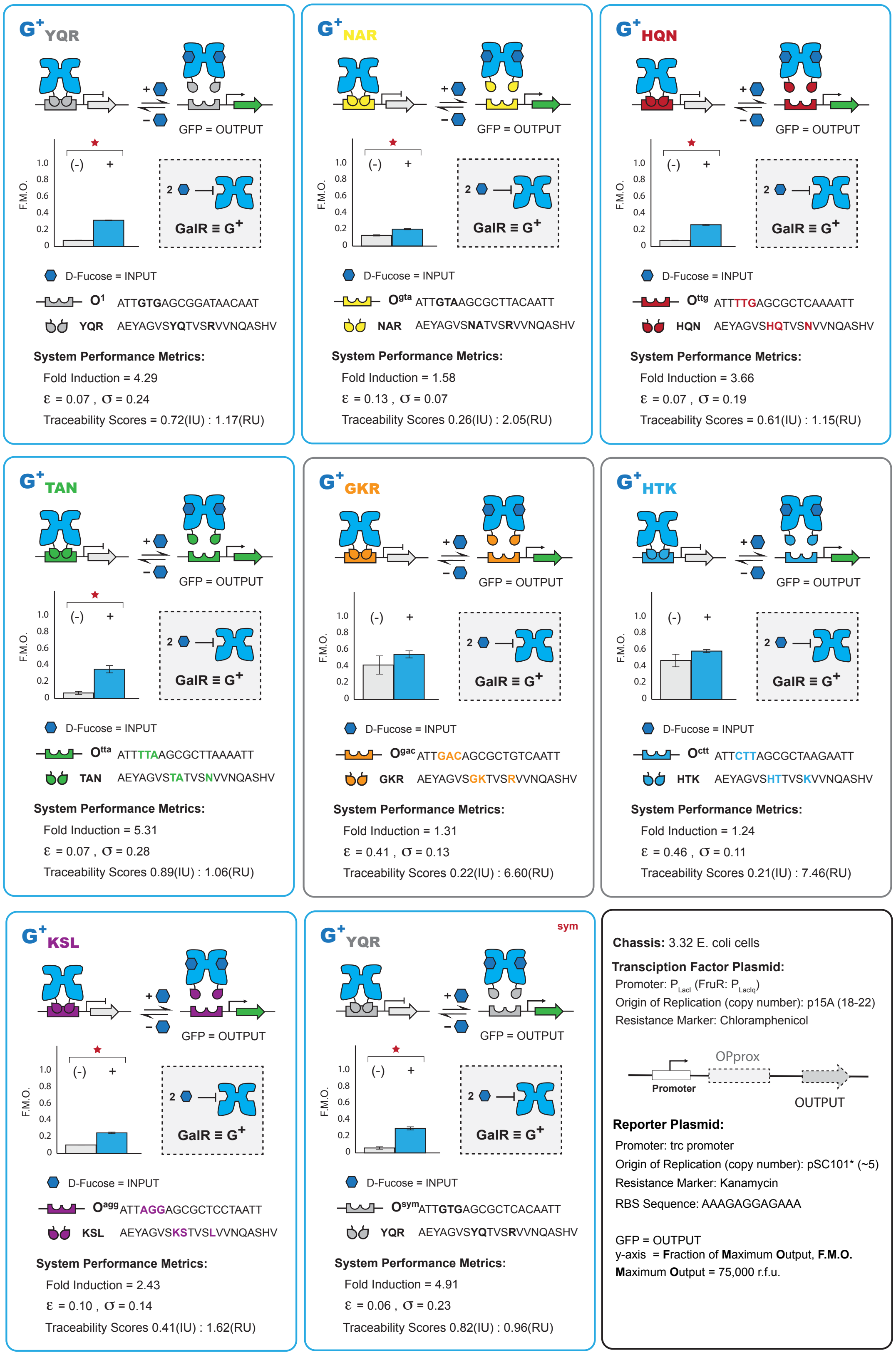

Supplementary Figure S1 – Part 5

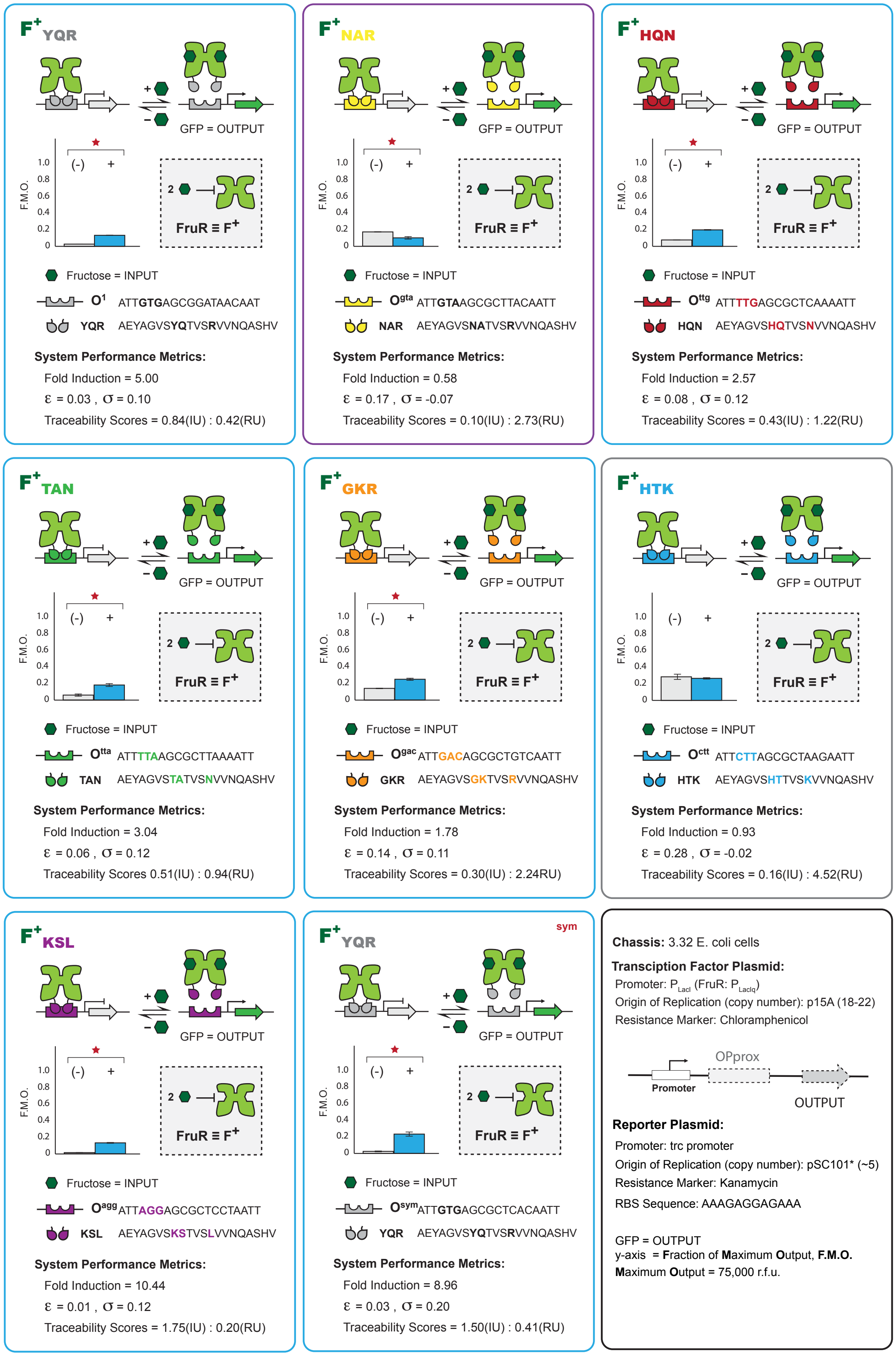

Supplementary Figure S1 – Part 6

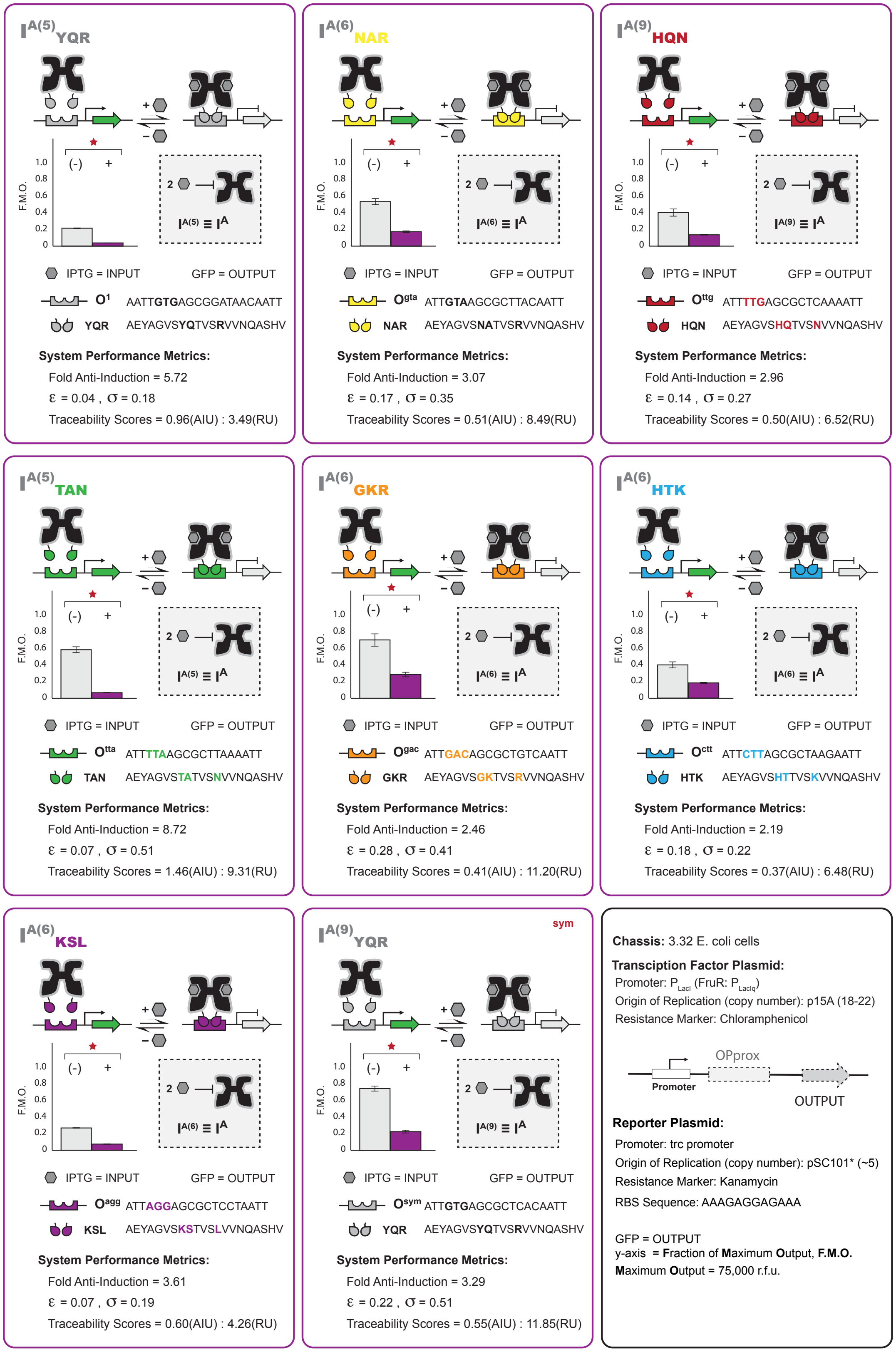

Supplementary Figure S1 – Part 7

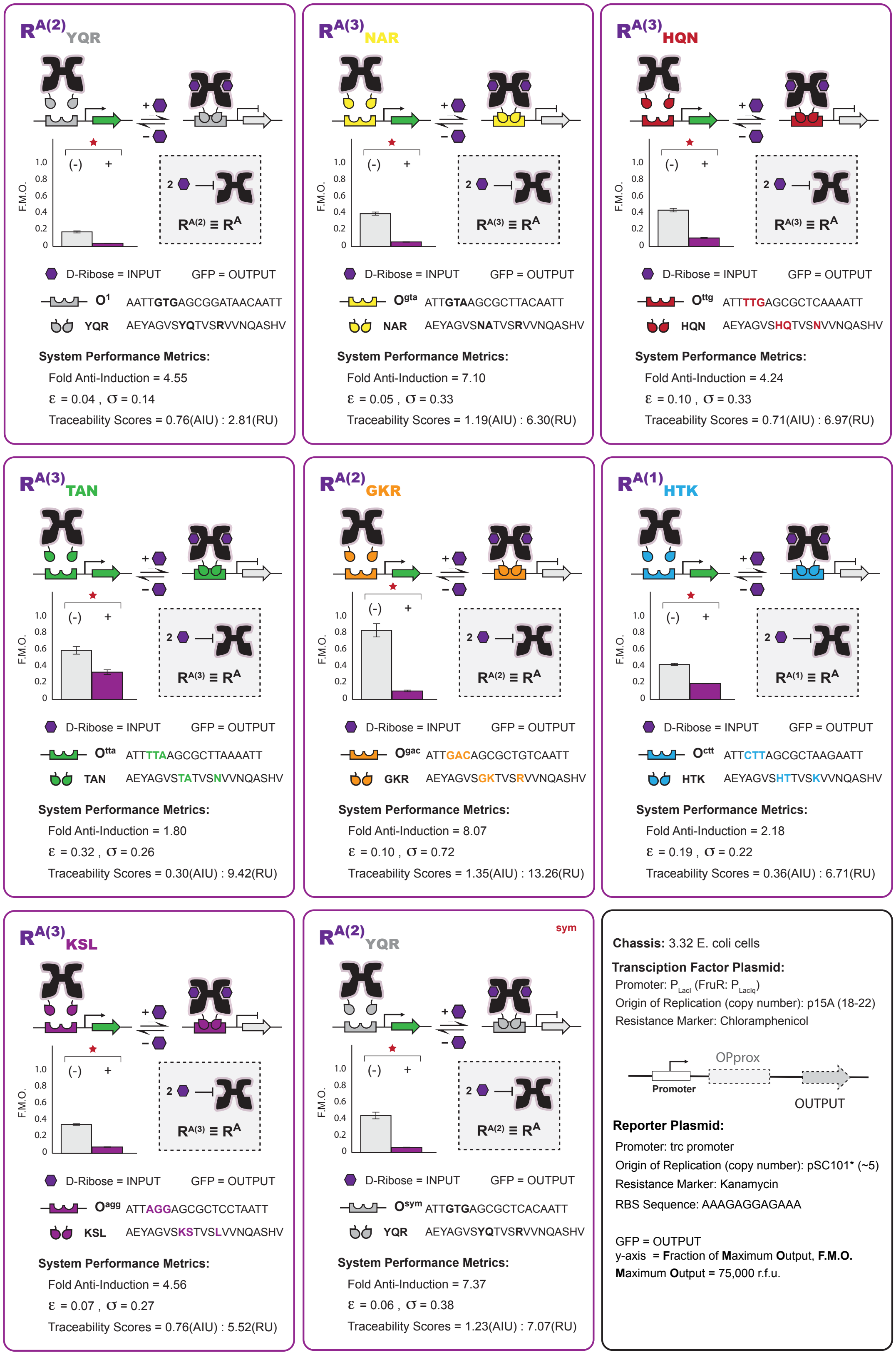

Supplementary Figure S1 – Part 8

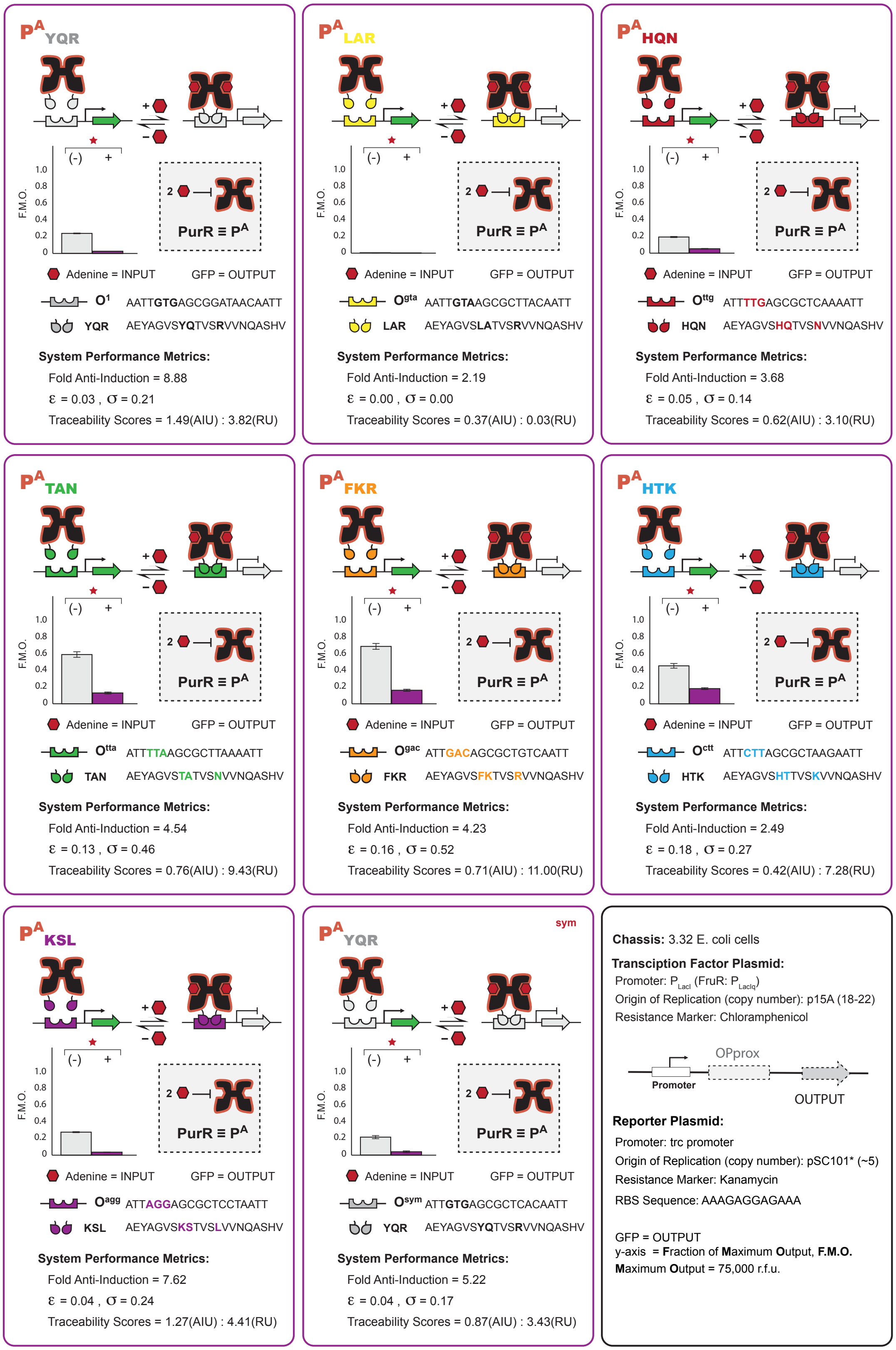

Supplementary Figure S1 – Part 9

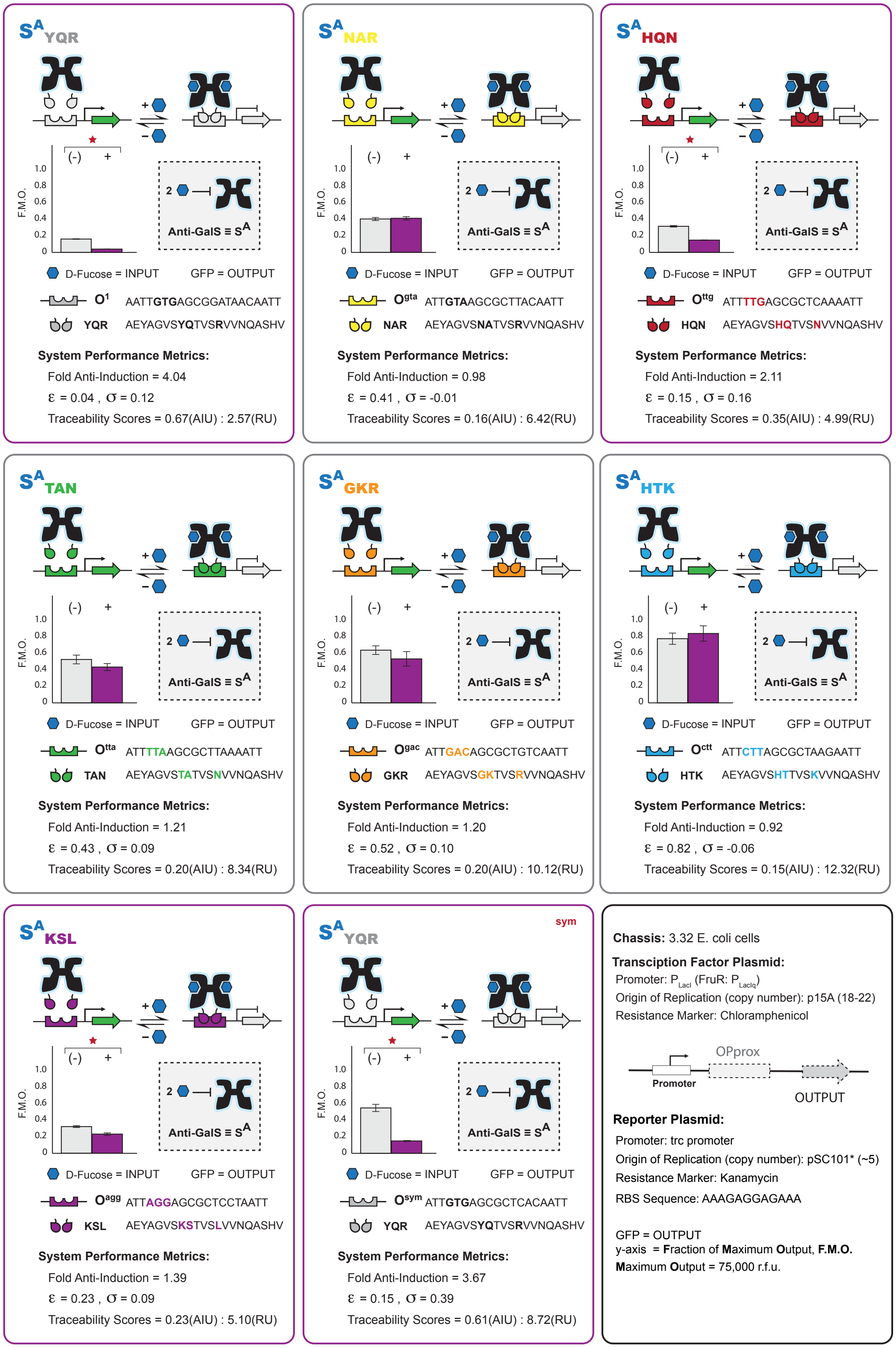

Supplementary Figure S1 – Part 10

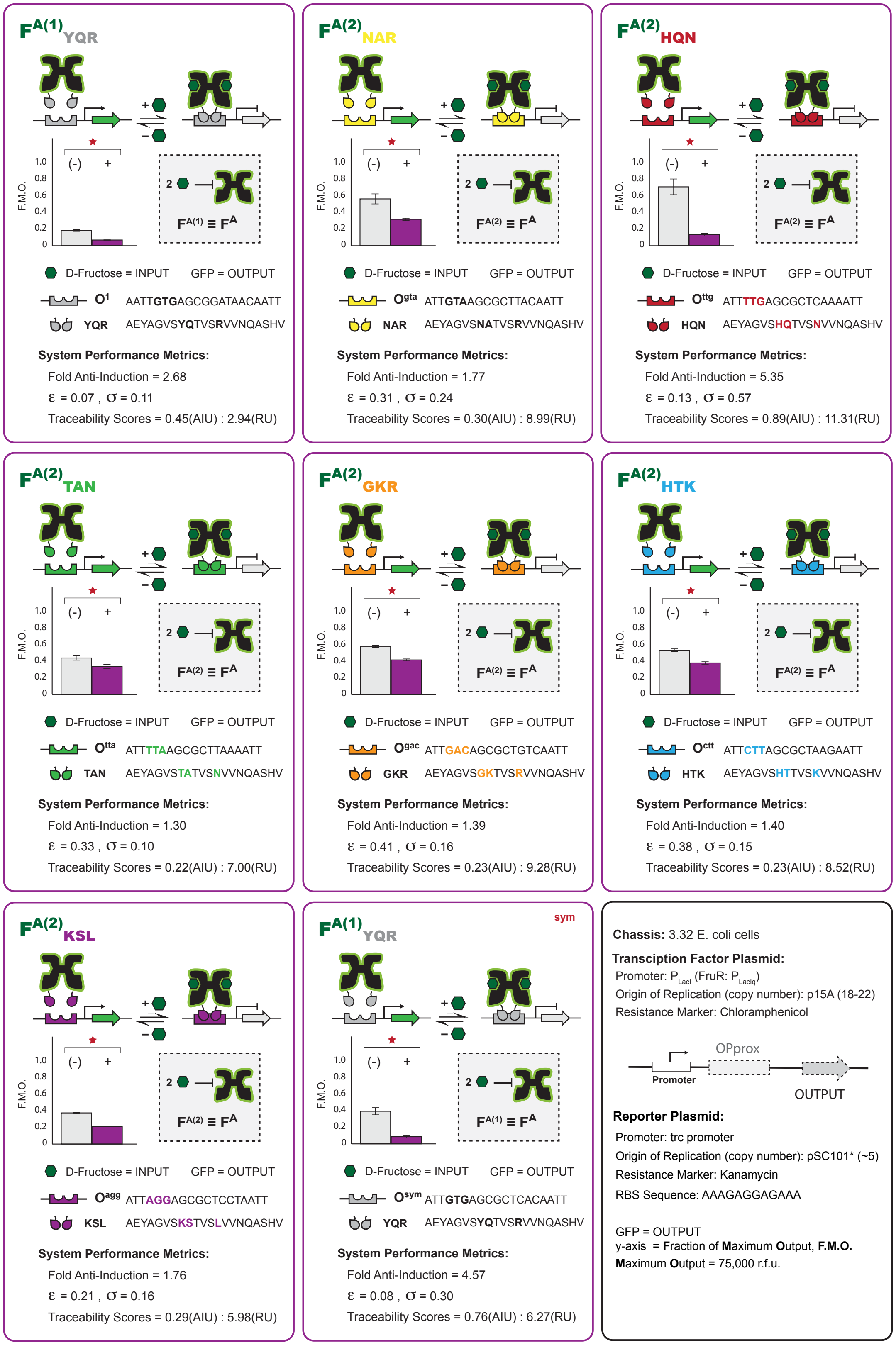

Supplementary Figure S1: PROXIMAL BUFFER gate and NOT gate performance cards. Each card displays experimental ON and OFF state OUTPUT values, INPUT signal type, DNA operator (ADR) type, and system performance metrics. Card outline color depicts the phenotype of each operation, consistent with **Figure S3**. (**S1 – Part 1**) LacI (I^{+}_{ADR}) performance cards, (**S1 – Part 2**) RbsR (R^{+}_{ADR}) performance cards, (**S1 – Part 3**) CelR (E^{+}_{ADR}) performance cards, (**S1 – Part 4**) GalR (G^{+}_{ADR}) performance cards, and (**S1 – Part 5**) FruR (F^{+}_{ADR}) performance cards. PROXIMAL NOT gate performance cards. Each card is analogous to those in Parts 1-5 but include respective metrics for NOT gates. (**S1 – Part 6**) Anti-LacI (I^{A}_{ADR}) performance cards, (**S1 – Part 7**) Anti-RbsR (R^{A}_{ADR}) performance cards, (**S1 – Part 8**) PurR (P^{A}_{ADR}) performance cards, (**S1 – Part 9**) Anti-GalS (S^{A}_{ADR}) performance cards, and (**S1 – Part 10**) Anti-FruR (F^{A}_{ADR}) performance cards.

Supplementary Figure S2 – Part 1

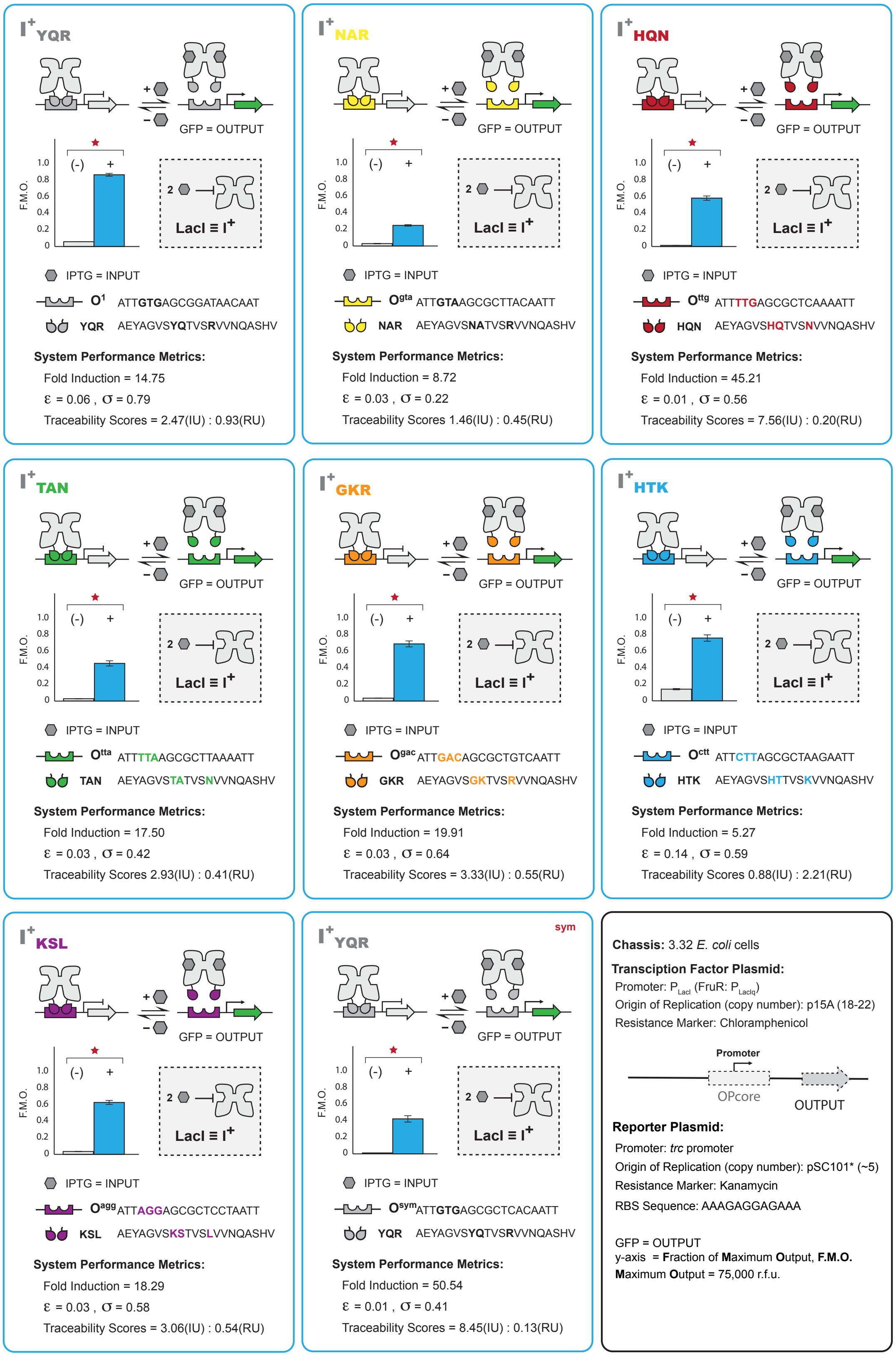

Supplementary Figure S2 – Part 2

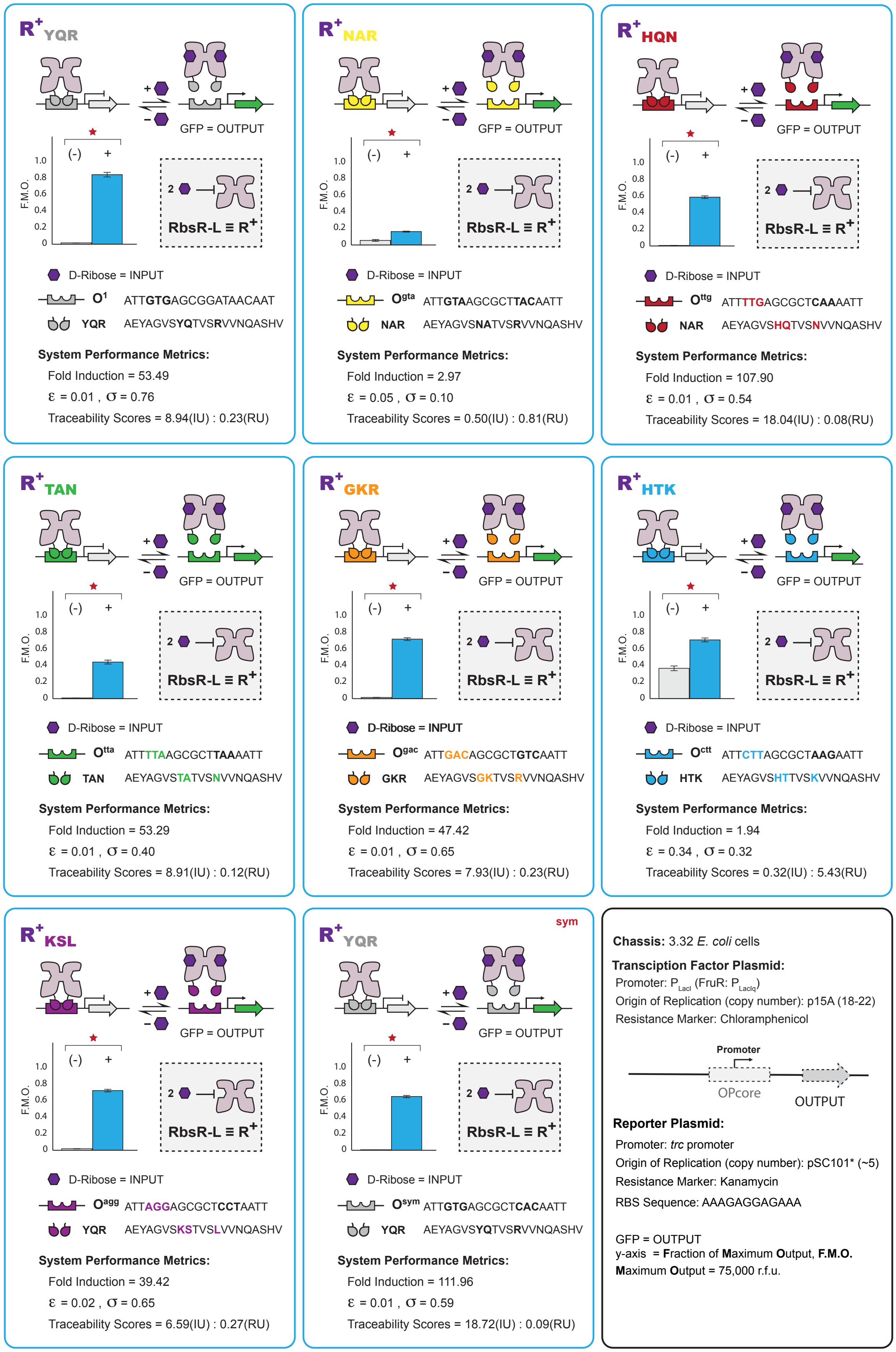

Supplementary Figure S2 – Part 3

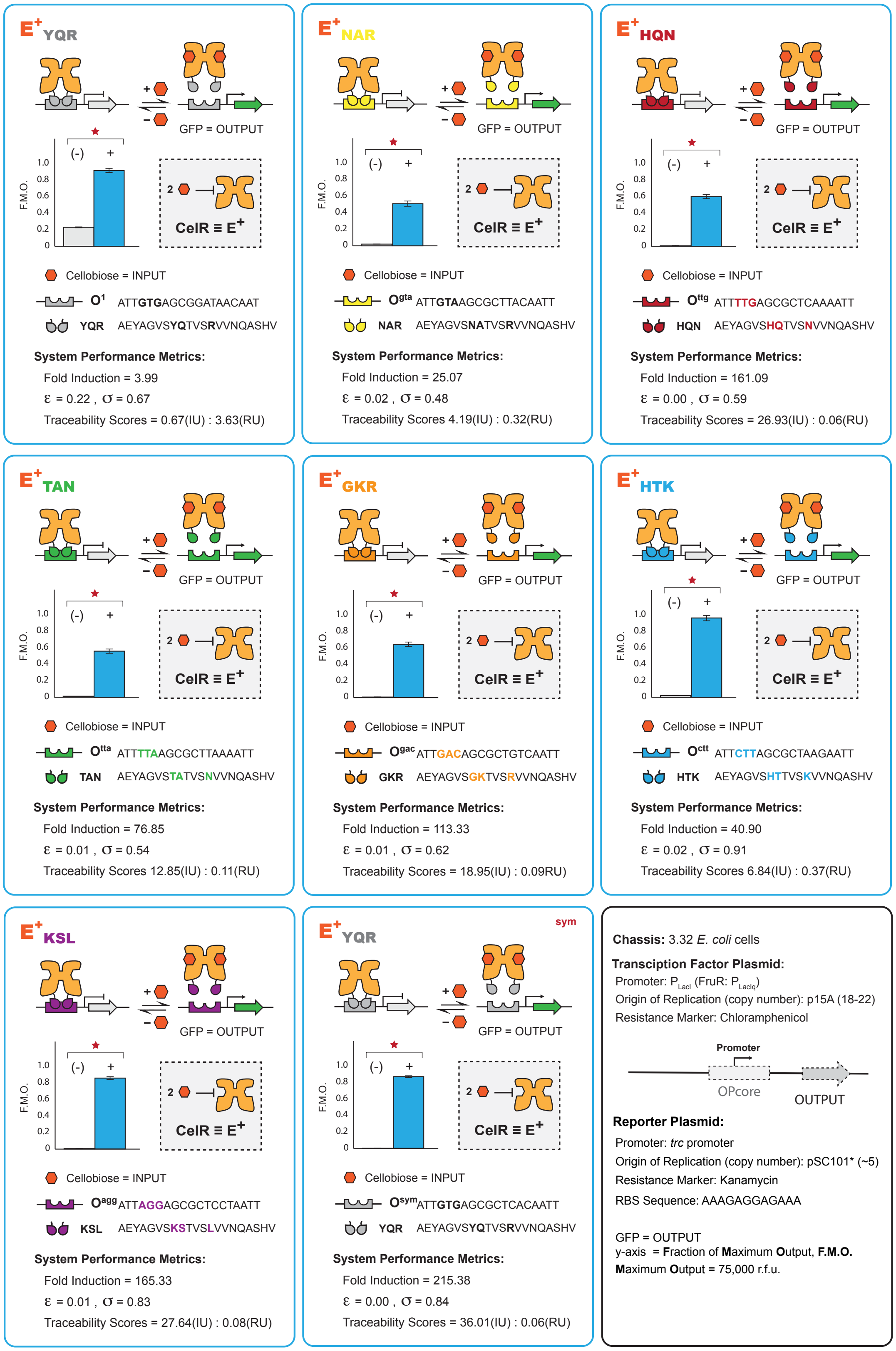

Supplementary Figure S2 – Part 4

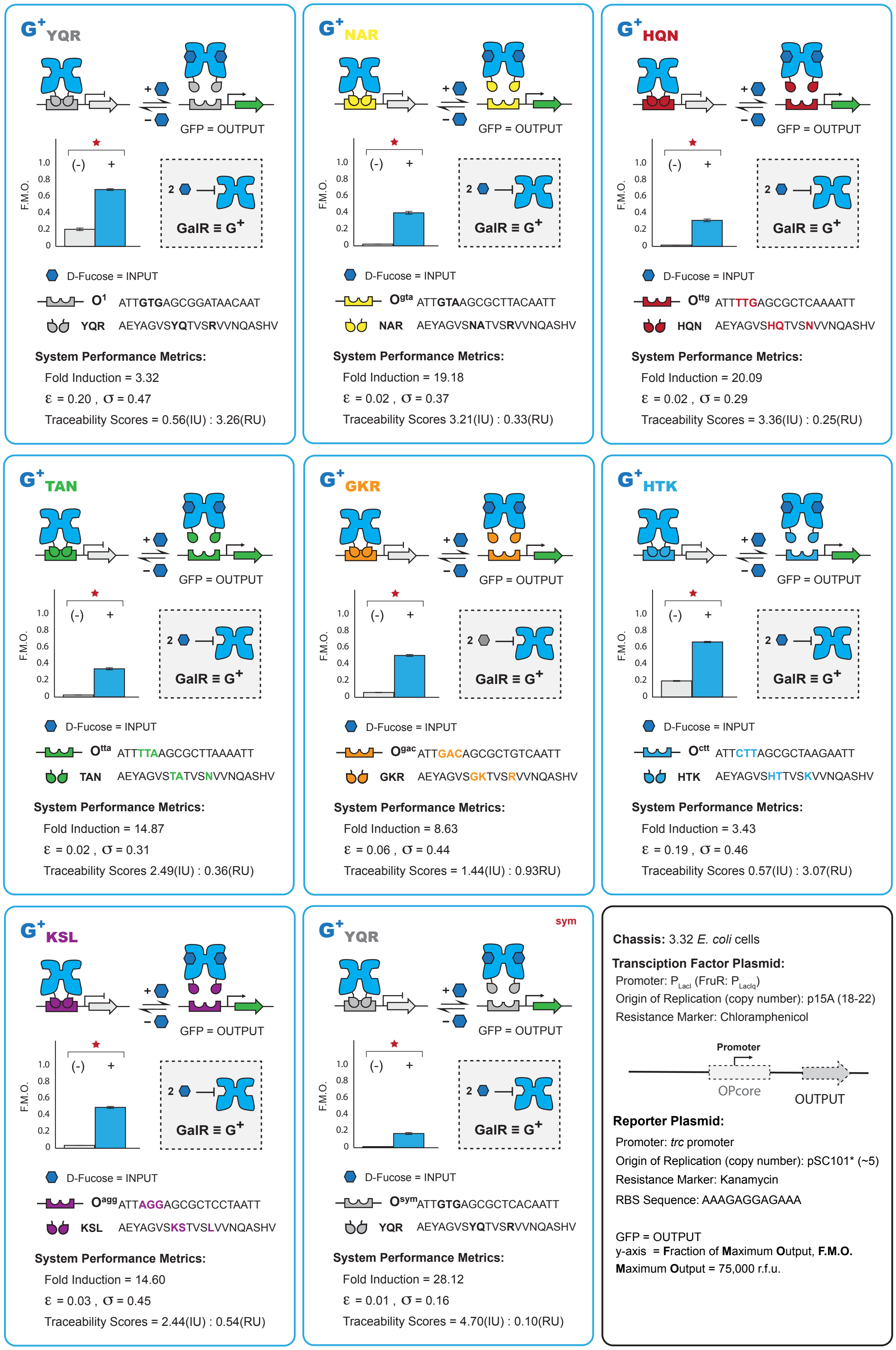

Supplementary Figure S2 – Part 5

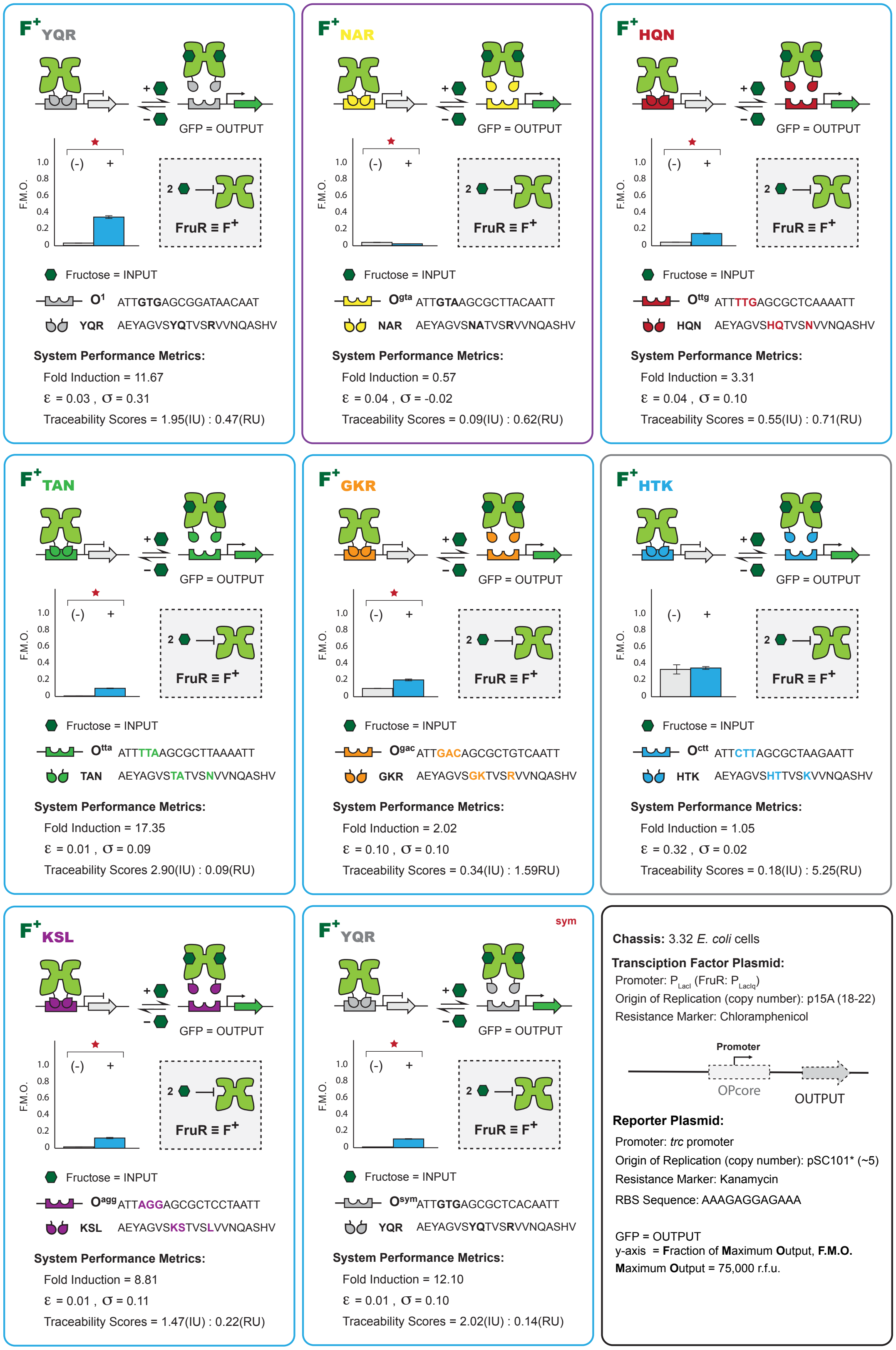

Supplementary Figure S2 – Part 6

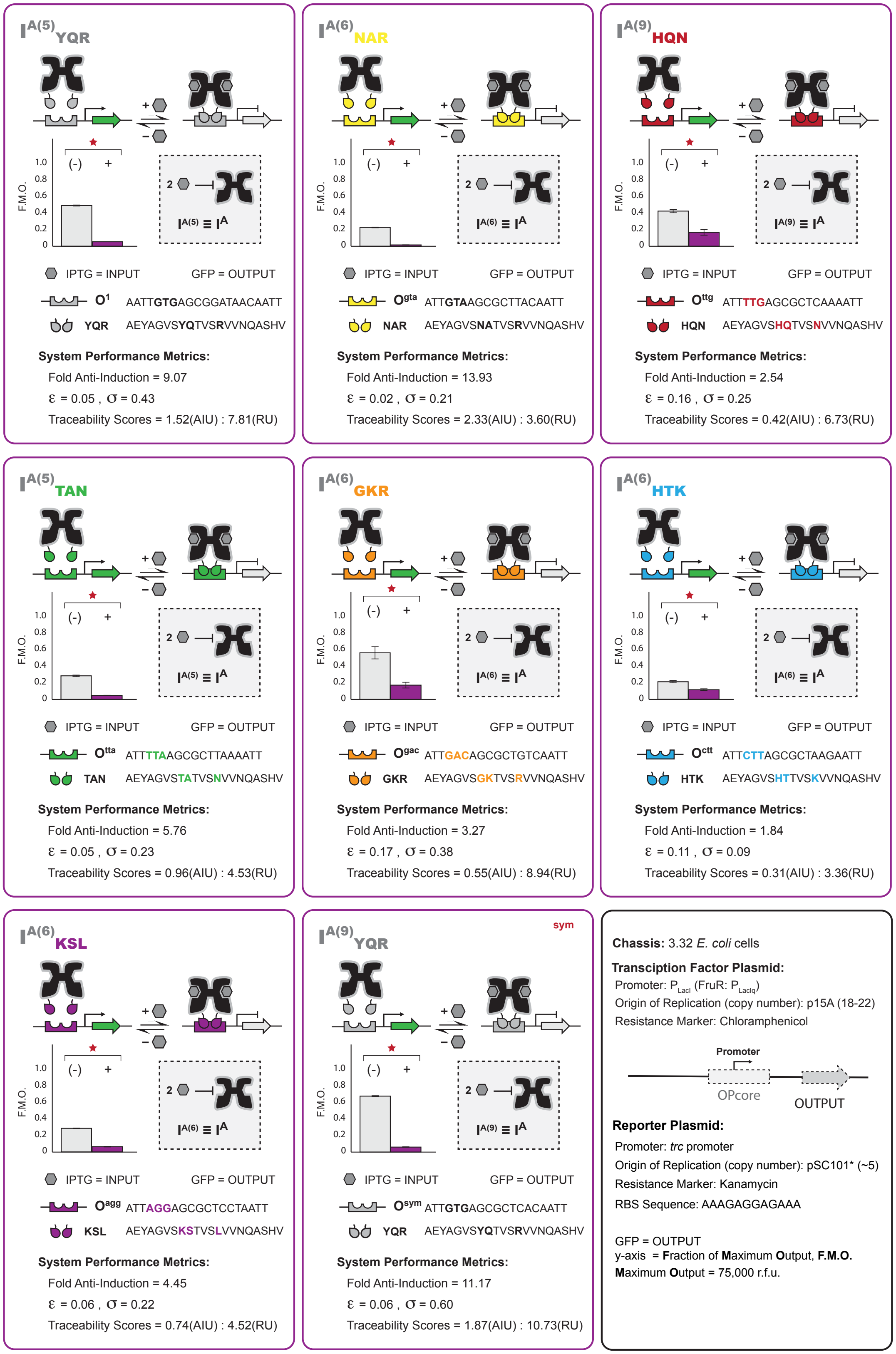

Supplementary Figure S2 – Part 7

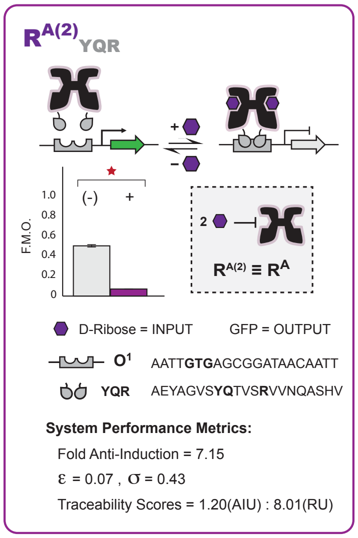

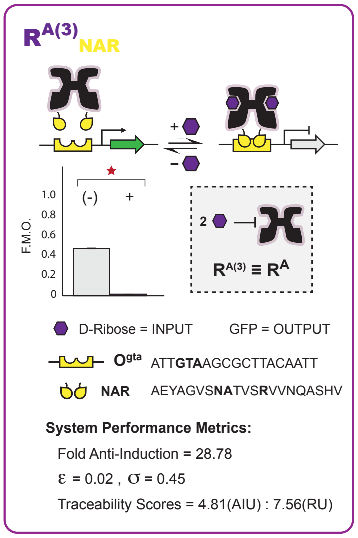

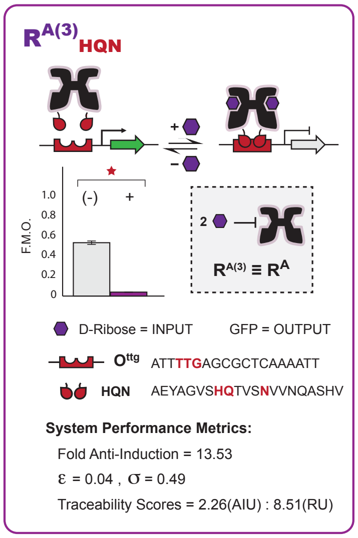

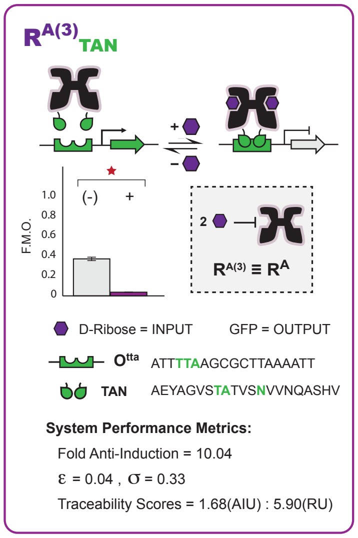

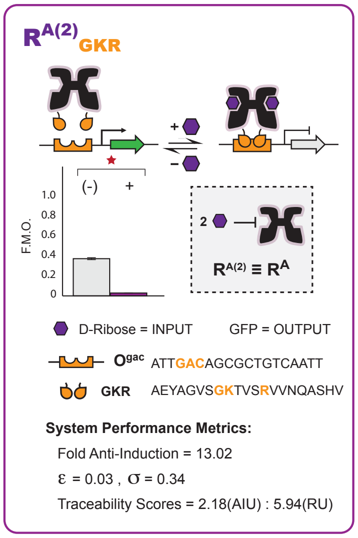

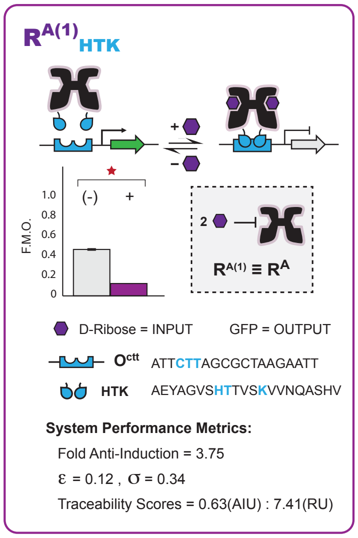

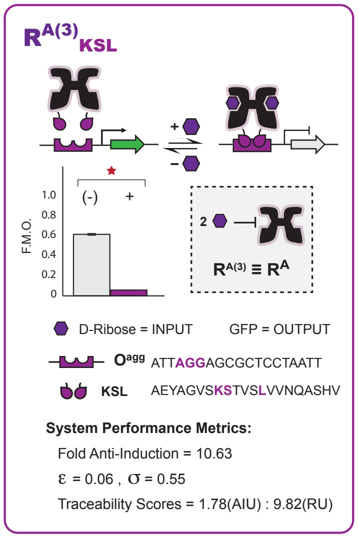

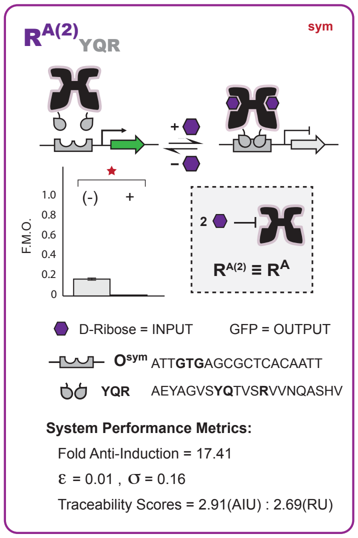

Chassis: 3.32 *E. coli* cells

Transciption Factor Plasmid:

Promoter: P_{LacI} (FruR: P_{LacIq})

Origin of Replication (copy number): p15A (18-22)

Resistance Marker: Chloramphenicol

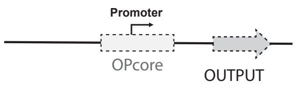

Reporter Plasmid:

Promoter: *trc* promoter

Origin of Replication (copy number): pSC101* (~5)

Resistance Marker: Kanamycin

RBS Sequence: AAAGAGGAGAAA

GFP = OUTPUT

y-axis = **F**raction of **M**aximum **O**utput, **F.M.O.**

Maximum **O**utput = 75,000 r.f.u.

Supplementary Figure S2 – Part 8

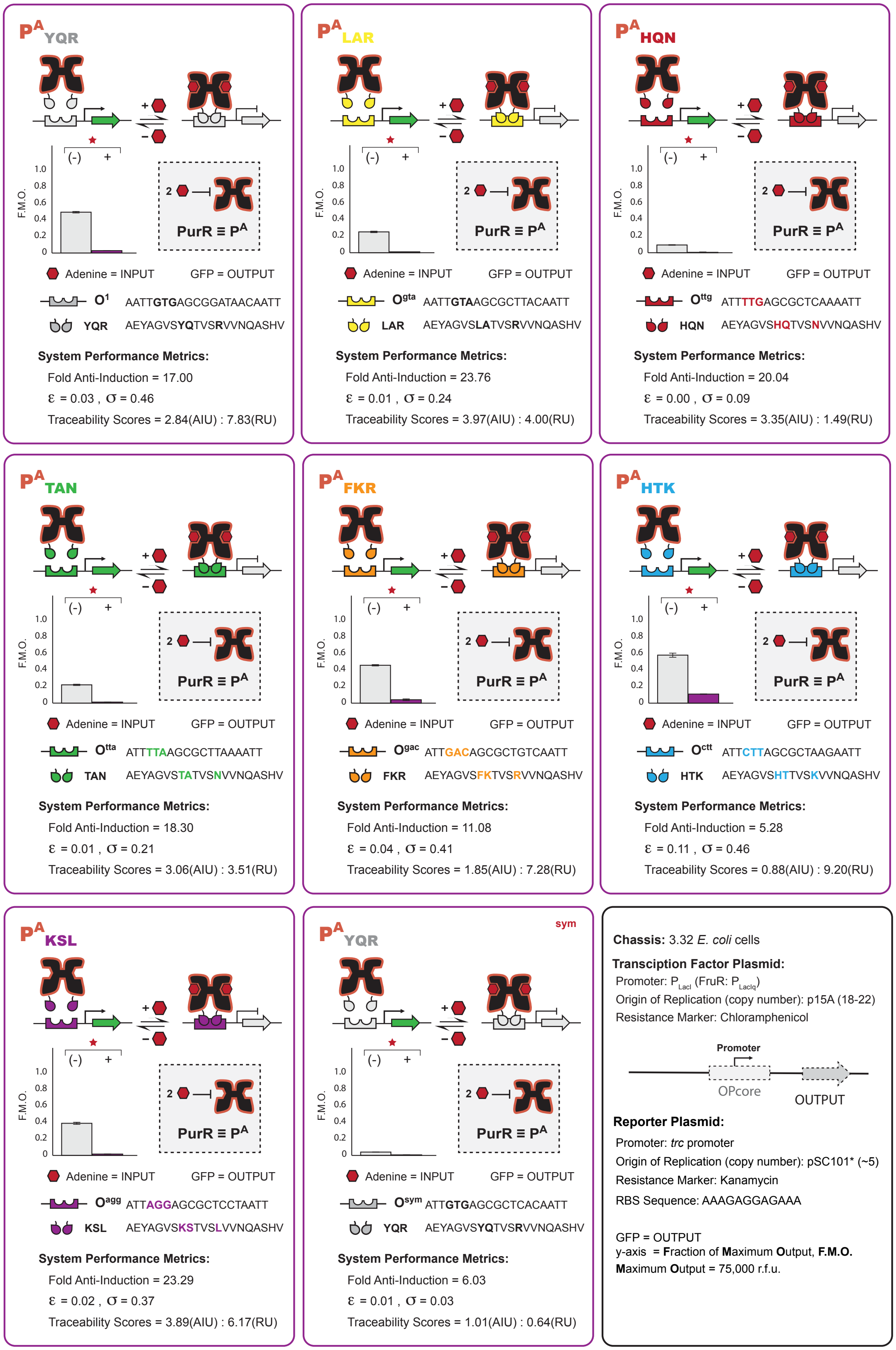

Supplementary Figure S2 – Part 9

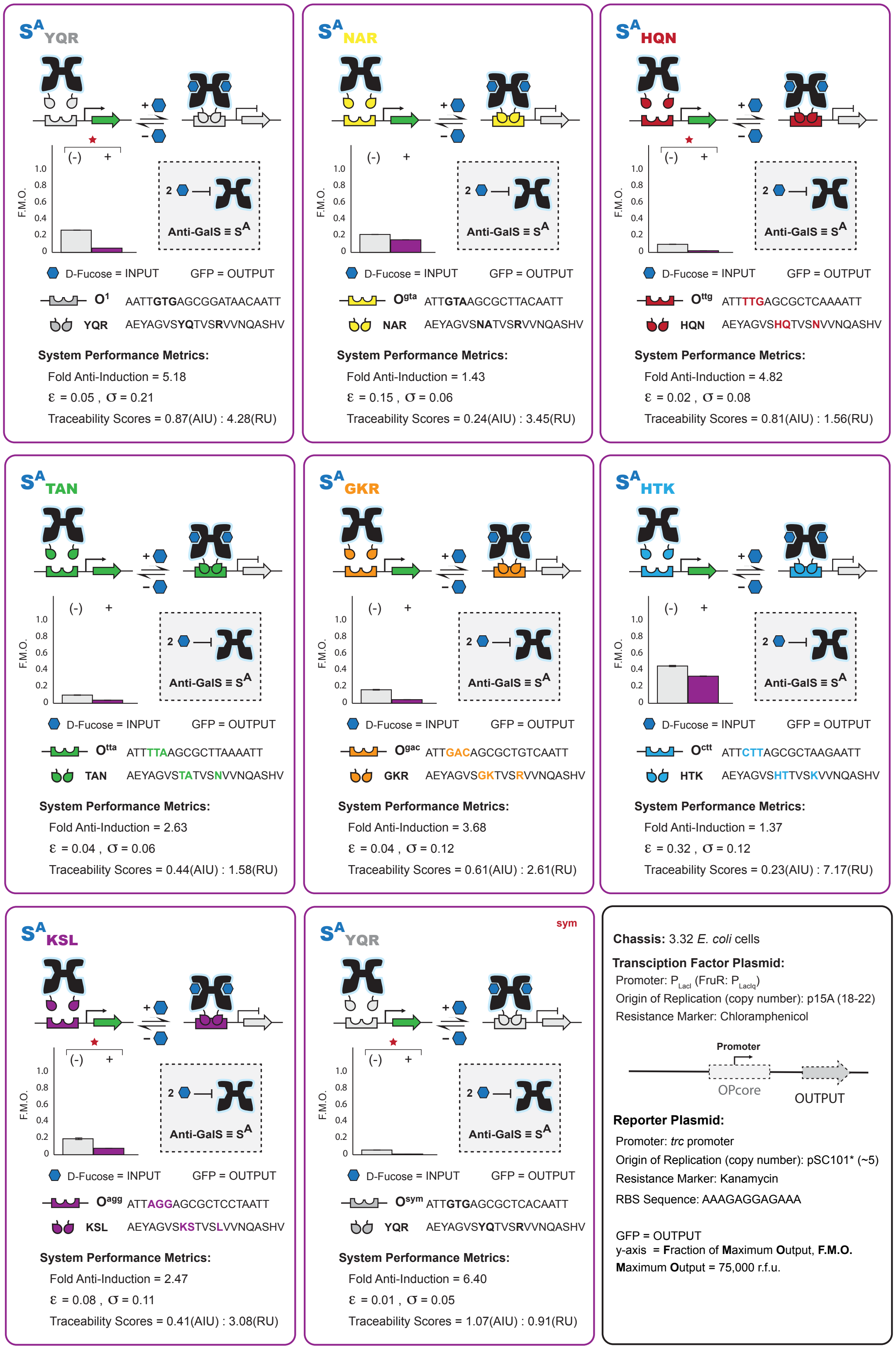

Supplementary Figure S2 – Part 10

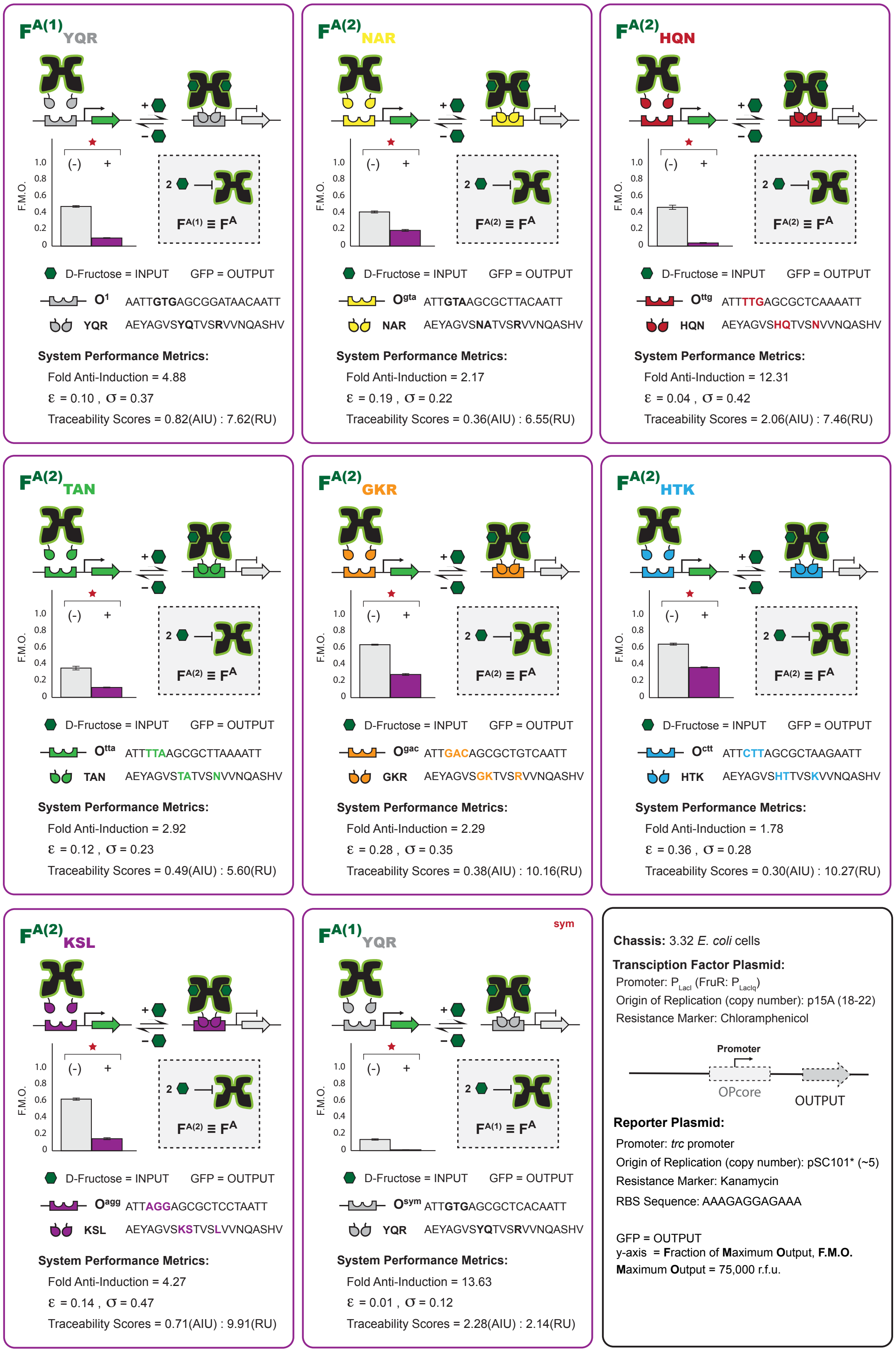

Supplementary Figure S2: CORE BUFFER gate and NOT gate performance cards. Each card displays experimental ON and OFF state OUTPUT values, INPUT signal type, DNA operator (ADR) type, and system performance metrics. Card outline color depicts the phenotype of each operation, consistent with **Figure S3**. (**S2 – Part 1**) LacI (I^{+}_{ADR}) performance cards, (**S2 – Part 2**) RbsR (R^{+}_{ADR}) performance cards, (**S2 – Part 3**) CelR (E^{+}_{ADR}) performance cards, (**S2 – Part 4**) GalR (G^{+}_{ADR}) performance cards, and (**S2 – Part 5**) FruR (F^{+}_{ADR}) performance cards. CORE NOT gate performance cards. Each card is analogous to those in Parts 1-5 but include respective metrics for NOT gates. (**S2 – Part 6**) Anti-LacI (I^{A}_{ADR}) performance cards, (**S2 – Part 7**) Anti-RbsR (R^{A}_{ADR}) performance cards, (**S2 – Part 8**) PurR (P^{A}_{ADR}) performance cards, (**S2 – Part 9**) Anti-GalS (S^{A}_{ADR}) performance cards, and (**S2 – Part 10**) Anti-FruR (F^{A}_{ADR}) performance cards.

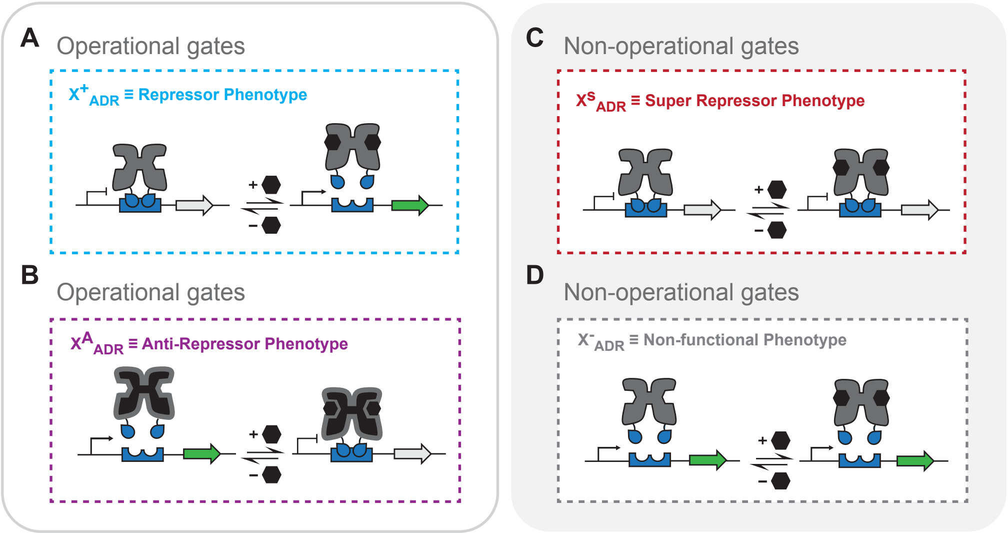

Supplementary Figure S3: Operational and non-operational SISO logic gates. Operational gates consist of either (**A**) repressor (X^{+}_{ADR}) – *i.e.*, BUFFER logic – or (**B**) anti-repressor (X^{A}_{ADR}) – *i.e.*, NOT logic – phenotypes. Classification of non-operational gates as either (**C**) super-repressor (X^{S}_{ADR}) or (**D**) non-functional (X^{-}_{ADR}) phenotypes.

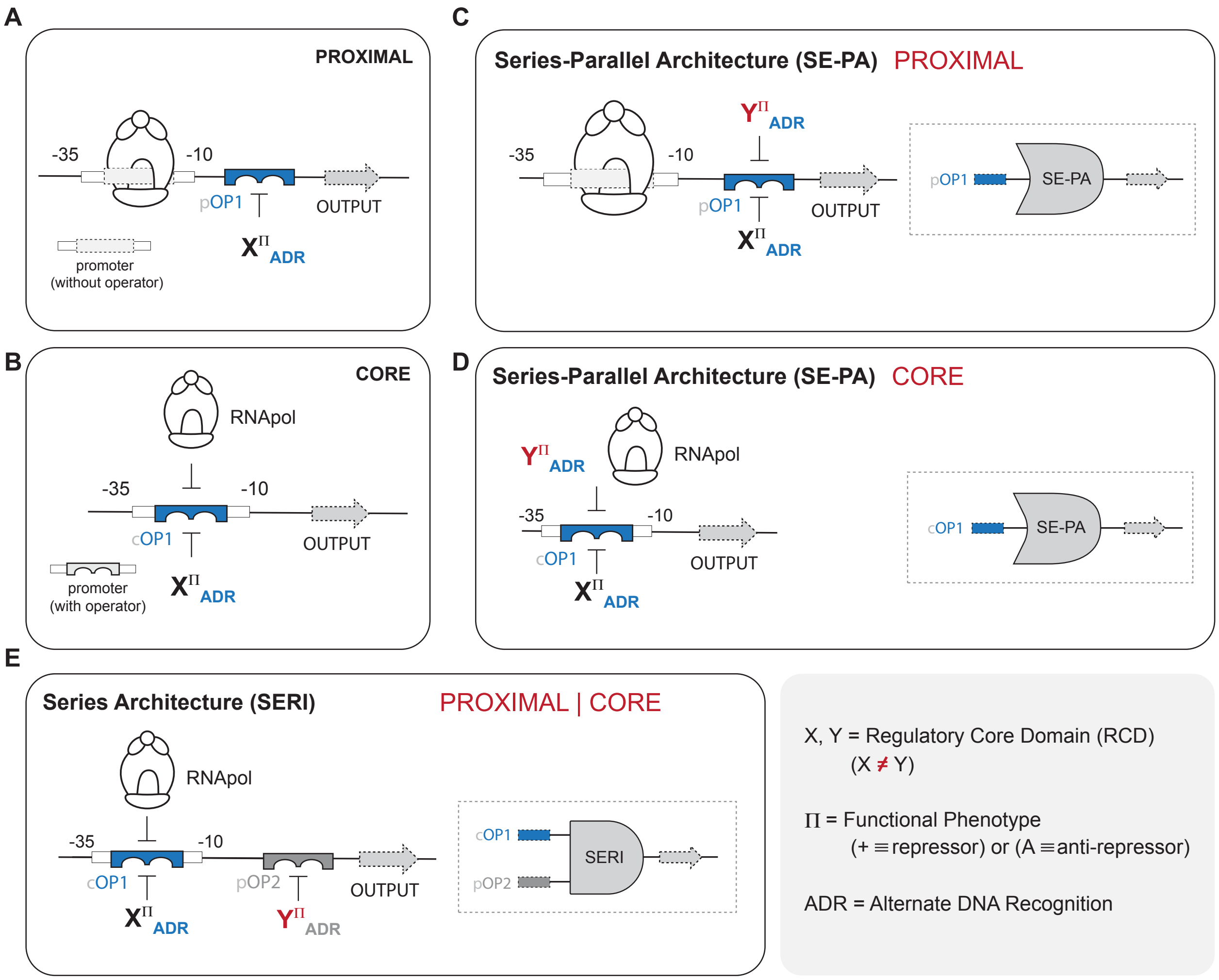

Supplementary Figure S4: Genetic architectures. (**A**) PROXIMAL architecture with an operator position downstream of the promoter. Transcription factor blocks RNA polymerase from transcribing DNA to regulate expression. (**B**) CORE architecture featuring an operator intercalated between the -35 and -10 hexamers of the synthetic *trc* promoter in *E. coli*. Transcription factor competes with RNA polymerase for binding DNA to regulate output expression. (**C-E**) Two-input architectures. (**C**) PROXIMAL SE-PA architecture as shown in (**A**) with two transcription factors directed to the operator. (**D**) CORE SE-PA architecture as shown in (**B**) with two transcription factors directed to the operator. (**E**) SERI architecture featuring a CORE operator and a second (non-synonymous) PROXIMAL operator.

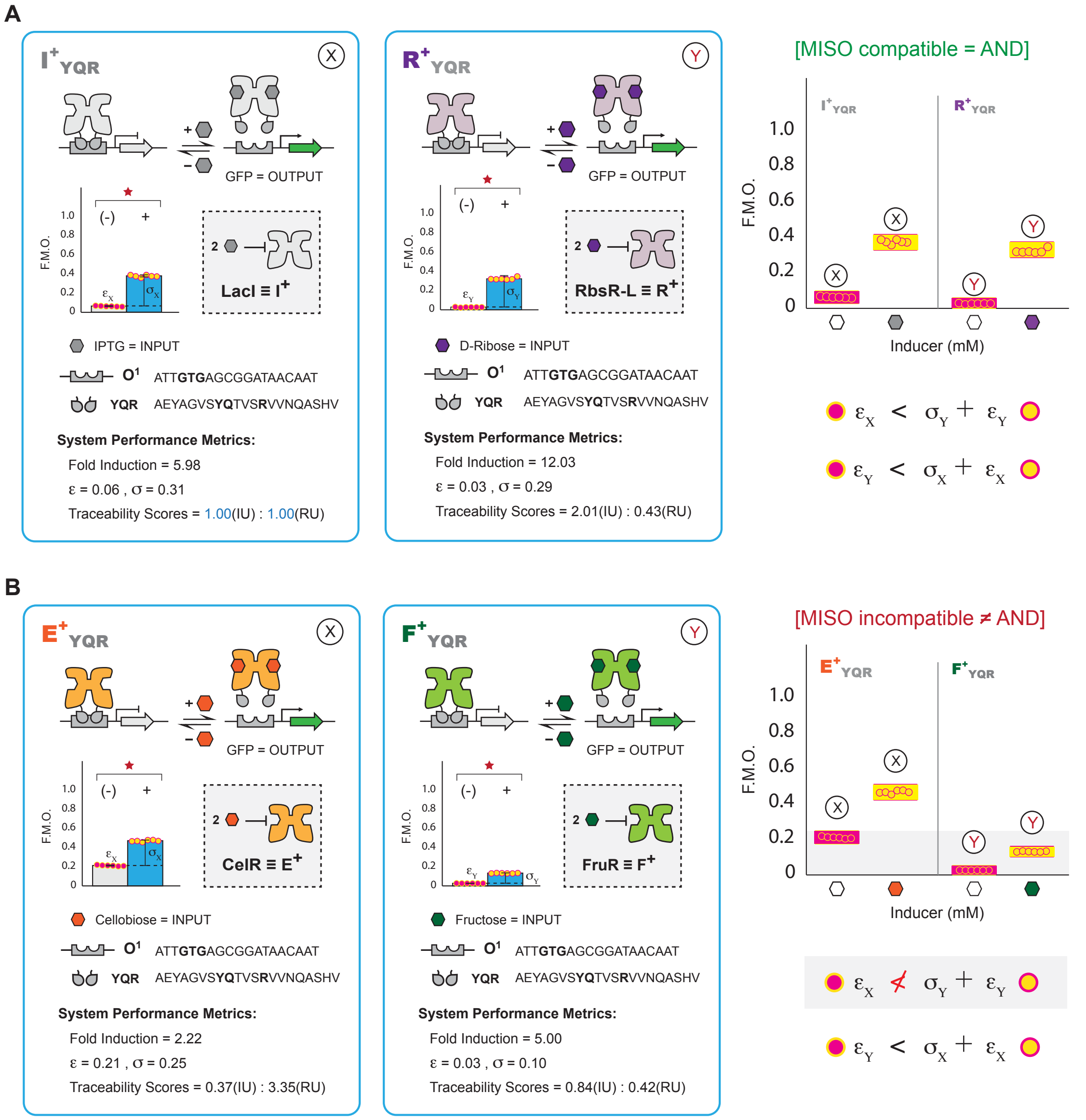

Supplementary Figure S5: Compatible and incompatible AND gate components. (**A**) Two compatible BUFFER operations constitute an AND gate when the OFF-state OUTPUT of either repressor is lower than the ON-state OUTPUT of the other. (**B**) A pair of BUFFER operations is incompatible when the inequalities shown in (**A**) are not met. Incompatible pairs are unlikely to produce a functional AND gate in that relative ON-state OUTPUT cannot be achieved across four input conditions.

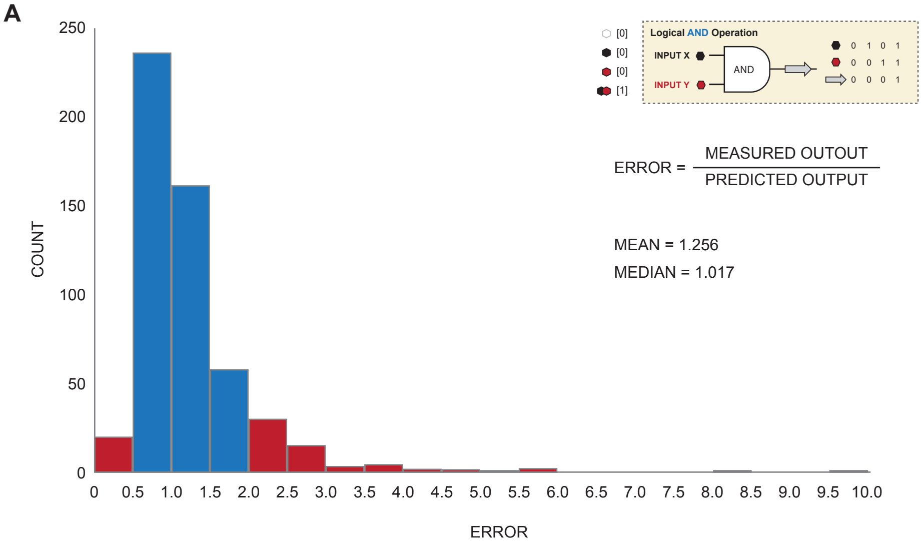

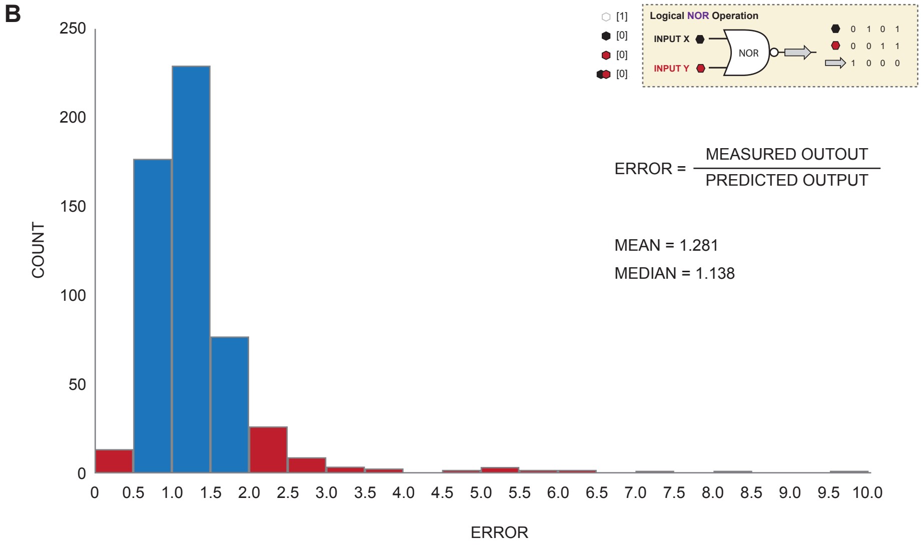

Supplementary Figure S6: Histograms of prediction error. (**A**) Error, defined as the ratio of measured to predicted OUTPUT, for all 133 SE-PA AND gates across all four INPUT conditions (error is equivalent to values given in plots illustrated in main text **Figure 4**). Values below 1 are overpredictions, and values above 1 are underpredictions. Blue bars indicate < 2-fold error and red bars indicate > 2-fold error in either direction. (**B**) Histograms of prediction error for 131 NOR gates given in main text **Figure 6**.

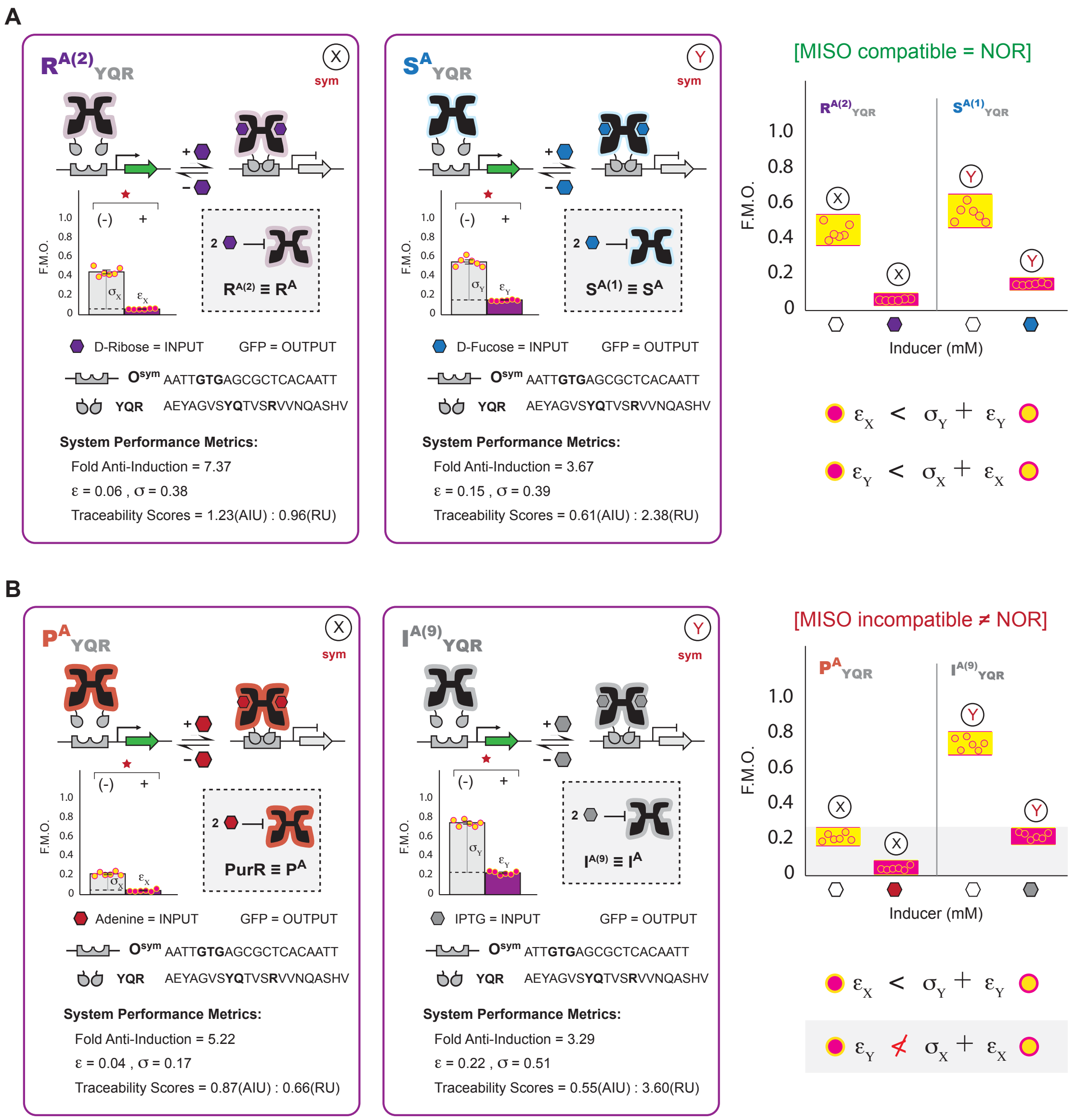

Supplementary Figure S7: Compatible and incompatible NOR gate components. (**A**) Two compatible NOT operations constitute a NOR gate when the OFF-state OUTPUT of either anti-repressor is lower than the ON-state OUTPUT of the other anti-repressor. (**B**) A pair of NOT operations is incompatible when the inequalities shown in (**A**) are not met. Incompatible pairs are unlikely to produce a functional NOR gate because relative ON-state OUTPUT cannot be achieved across four input conditions.

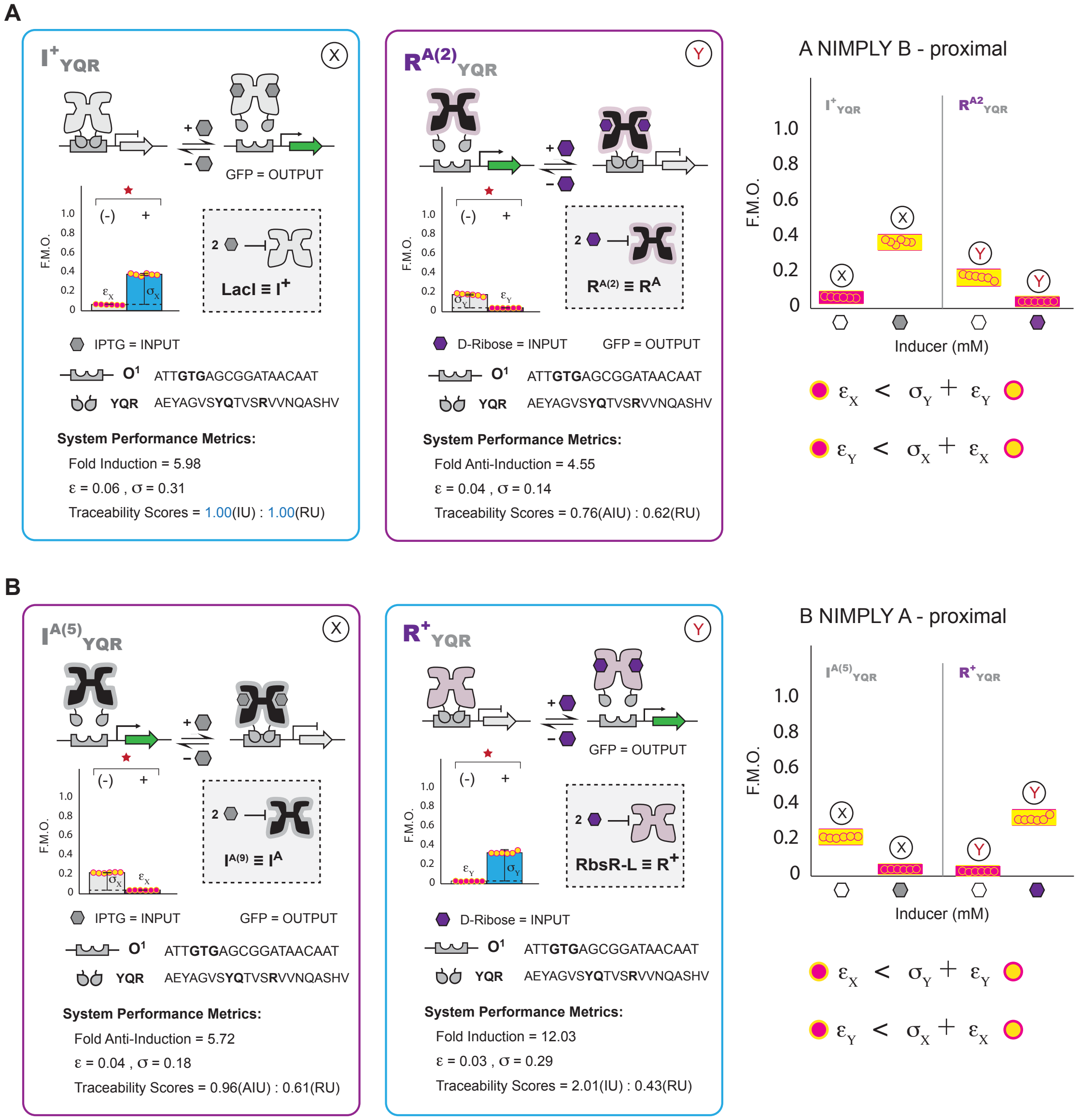

Supplementary Figure S8: (**A-B**) PROXIMAL SE-PA NIMPLY logic. (**A**) An example A NIMPLY B logic gate comprised of an I^{+}_{YQR} BUFFER and R^{A2}_{YQR} NOT operation both directed to the O^{1} PROXIMAL SE-PA genetic architecture. (**B**) The complementary B NIMPLY A operation utilizing an I^{A5}_{YQR} NOT and R^{+}_{YQR} BUFFER operation.

C

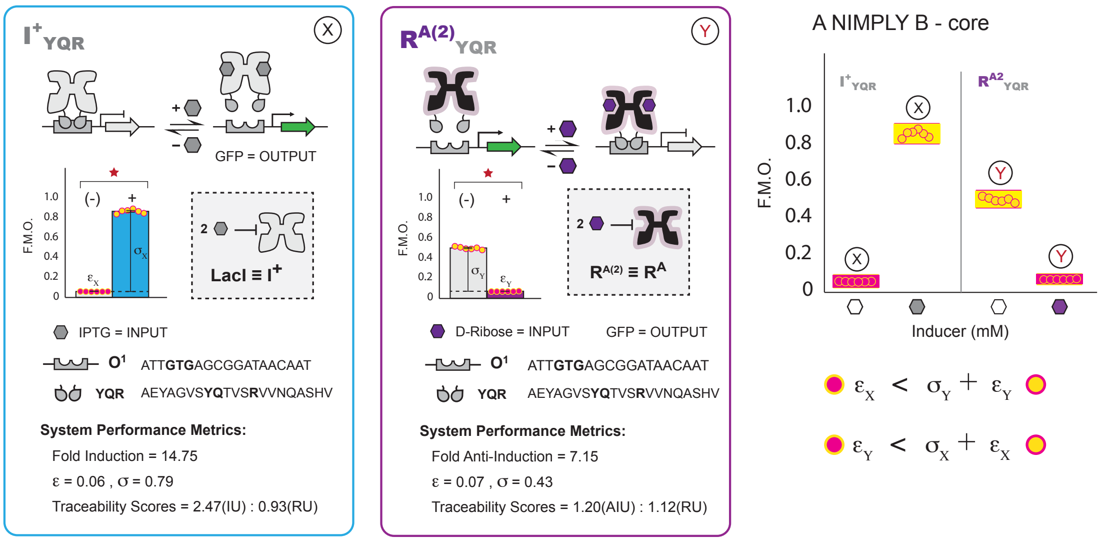

D

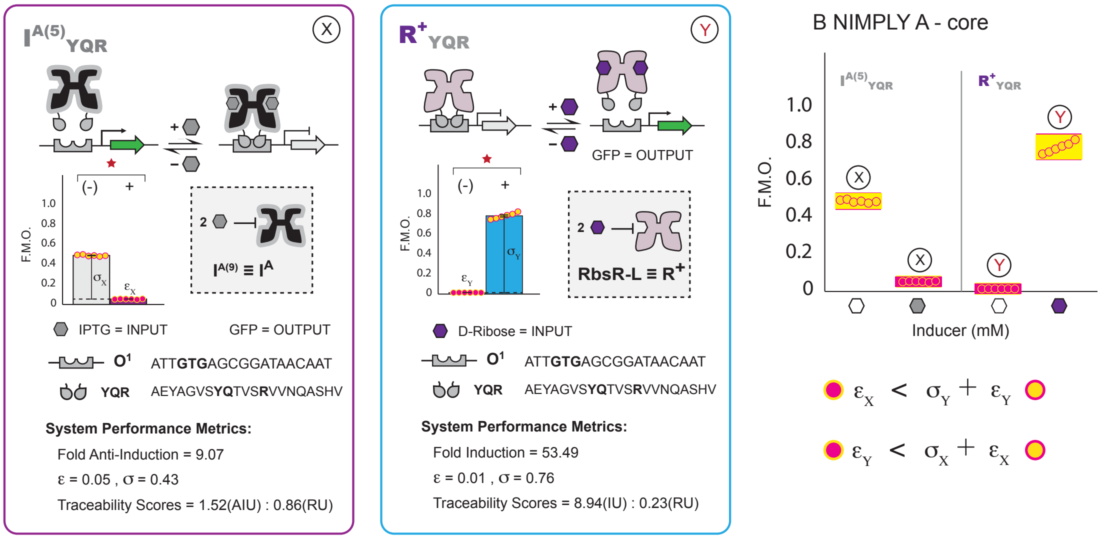

Supplementary Figure S8: (**C-D**) CORE SE-PA NIMPLY logic. (**C**) The example A NIMPLY B logic gate shown in (**A**) at the CORE operator position. (**D**) The complimentary B NIMPLY A operation shown in (**B**) also directed to the CORE position. All NIMPLY gates respond to the same two INPUTs, however, variations of transcription factor phenotypes and DNA operator position yield differences in performance.

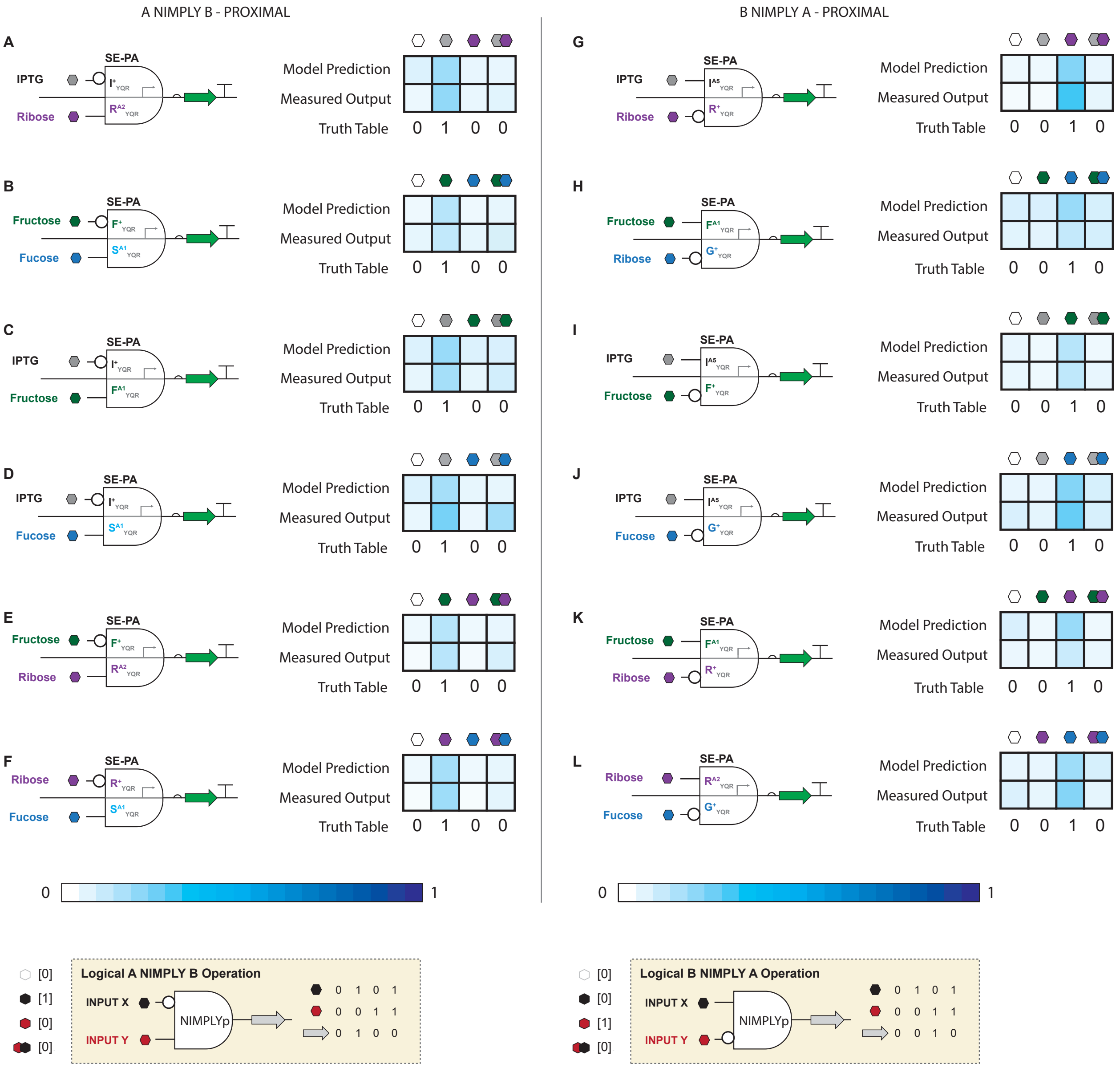

Supplementary Figure S9: PROXIMAL SE-PA NIMPLY logic. Analogous to **Figure 7** but at the proximal operator position. (**A-F**) X NIMPLY Y logic employing a repressor (X^{+}_{ADR}) which responds to INPUT A and anti-repressor (Y^{A}_{ADR}) which responds to INPUT B. (**G-L**) Complimentary B NIMPLY A logic utilizing an anti-repressor (X^{A}_{ADR}) and repressor (Y^{+}_{ADR}).

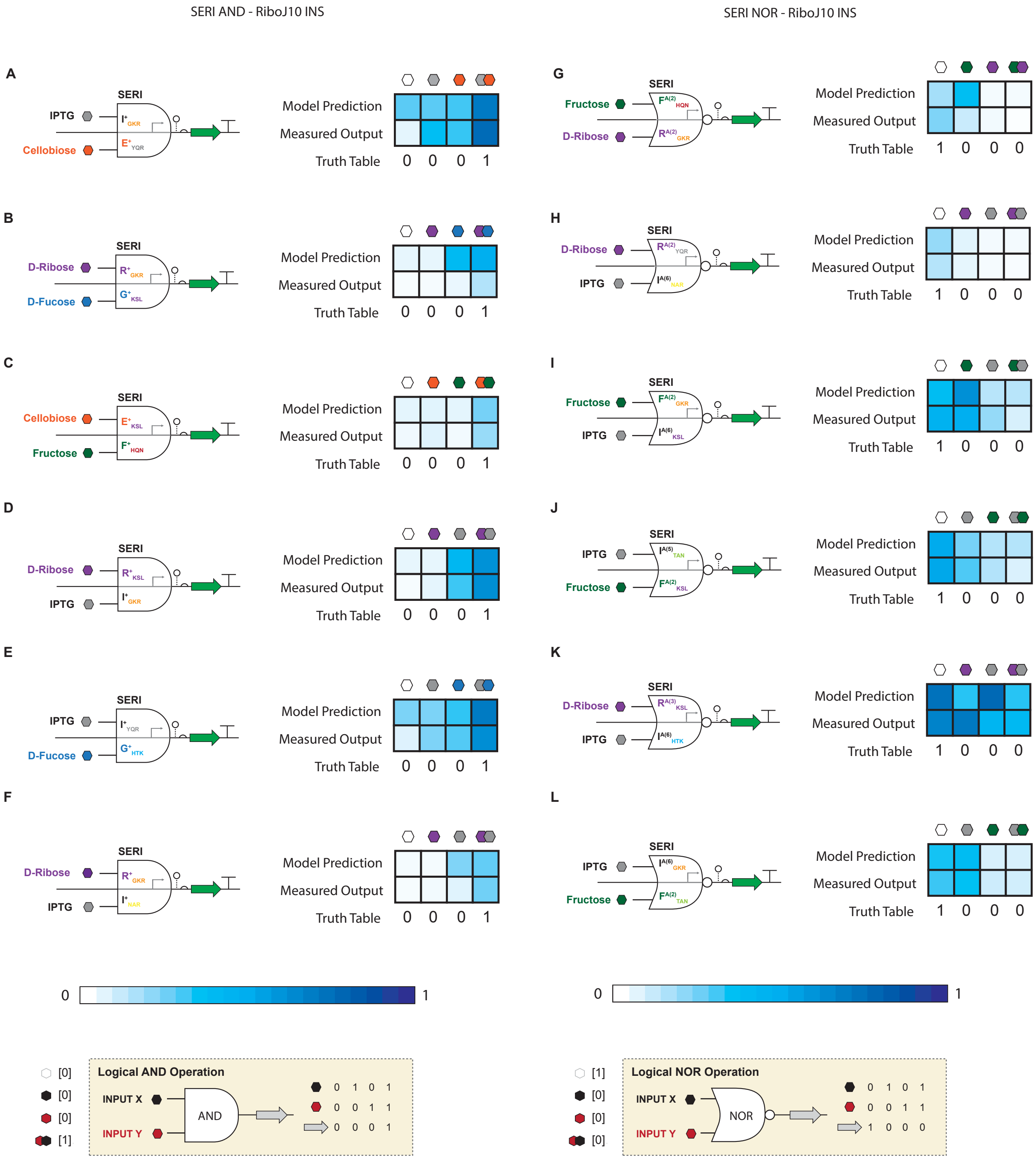

Supplementary Figure S10: Results for 6 insulated SERI AND operations and 6 insulated SERI NOR operations (analogous gates to those in **Figure 9**, with the addition of the genetic insulator RiboJ10). (**A-F**) AND logic gates employing a repressor (X^{+}_{ADR}) directed to a cognate PROXIMAL operator (upper input), and second repressor (Y^{+}_{ADR}) directed to a cognate CORE operator (lower input). Results for OUTPUT prediction using SERI SISO parameters and measured OUTPUT are shown on the right. (**G-L**) Insulated NOR logic gates employing anti-repressors X^{A}_{ADR} and Y^{A}_{ADR} *via* the SERI genetic architecture.

(1) Nielsen, A. A.; Der, B. S.; Shin, J.; Vaidyanathan, P.; Paralanov, V.; Strychalski, E. A.; Ross, D.; Densmore, D.; Voigt, C. A. Genetic circuit design automation. *Science* **2016**, *352* (6281), aac7341.
(2) Swint-Kruse, L.; Matthews, K. S., Allostery in the LacI/GalR family: variations on a theme. *Curr Opin Microbiol* **2009,** *12* (2), 129-137.
(3) Huang, B. D.; Groseclose, T. M.; Wilson, C. J. Transcriptional programming in a Bacteroides consortium. *Nat. Commun.* **2022**, *13* (1), 3901.
